# Bridging the Gap Between Validation and Implementation of Non-Animal Veterinary Vaccine Potency Testing Methods

**DOI:** 10.3390/ani1040414

**Published:** 2011-11-29

**Authors:** Samantha Dozier, Jeffrey Brown, Alistair Currie

**Affiliations:** 1 People for the Ethical Treatment of Animals, 501 Front Street, Norfolk, VA 23510, USA; E-Mail: samanthad@peta.org; 2 People for the Ethical Treatment of Animals Foundation, P.O. Box 36678, London, SE1 1YE, UK; E-Mail: alistairc@peta.org.uk

**Keywords:** non-animal, vaccine, safety, potency, regulatory testing, laboratory animals, implementation

## Abstract

**Simple Summary:**

Many vaccines are tested for quality in experiments that require the use of large numbers of animals in procedures that often cause significant pain and distress. Newer technologies have fostered the development of vaccine quality control tests that reduce or eliminate the use of animals, but the availability of these newer methods has not guaranteed their acceptance by regulators or use by manufacturers. We discuss a strategic approach that has been used to assess and ultimately increase the use of non-animal vaccine quality tests in the U.S. and U.K.

**Abstract:**

In recent years, technologically advanced high-throughput techniques have been developed that replace, reduce or refine animal use in vaccine quality control tests. Following validation, these tests are slowly being accepted for use by international regulatory authorities. Because regulatory acceptance itself has not guaranteed that approved humane methods are adopted by manufacturers, various organizations have sought to foster the preferential use of validated non-animal methods by interfacing with industry and regulatory authorities. After noticing this gap between regulation and uptake by industry, we began developing a paradigm that seeks to narrow the gap and quicken implementation of new replacement, refinement or reduction guidance. A systematic analysis of our experience in promoting the transparent implementation of validated non-animal vaccine potency assays has led to the refinement of our paradigmatic process, presented here, by which interested parties can assess the local regulatory acceptance of methods that reduce animal use and integrate them into quality control testing protocols, or ensure the elimination of peripheral barriers to their use, particularly for potency and other tests carried out on production batches.

## 1. Introduction

Vaccines—preparations, generally isolated from living organisms, used to elicit an immune response—are considered essential tools for preventing infectious disease and have been incorporated into global, national and regional initiatives to prevent and manage illness in humans and animals [1-3]. Historically, human and animal vaccines have been developed in close proximity, using similar methods of production and evaluation [4]. Vaccines have been effective components of efforts designed to control a range of animal diseases, including rabies, foot and mouth disease, parvovirus, feline herpesvirus, feline calicivirus, and canine hepatitis [5].

Although many of the most successful disease prevention and mitigation strategies in the last century have incorporated vaccination campaigns, vaccine production and quality assurance processes account for the deaths of millions of animals each year due to the testing methods that have been developed to ensure that final products conform to specific quality parameters [6].

### 1.1. History of Vaccine Development and Challenge Tests

The vaccination process was popularly demonstrated using immunogenic material from cows by Edward Jenner in 1796, but humans were among the first subjects used to experimentally identify the principles of tests that would eventually become established as the primary approach to assessing human and animal vaccine efficacy [7]. After inoculating eight-year-old James Phipps with material from a human cowpox blister, Jenner exposed him—and later other individuals who had received similar inoculations—to smallpox, demonstrating the protective capacity of inoculations derived from pathogenic agents [8]. By 1853, Louis Willems had provided a similar demonstration of protection from contagious bovine pleuropneumonia by inoculating animals with serous fluid from the lungs of infected cows [4]. This process, in which vaccination was demonstrated to provide protection against disease through the intentional exposure to a virulent pathogen, is fundamentally similar to the challenge (or ‘vaccination-challenge’) process that has become standardized in a suite of tests carried out in animals and used to establish the safety and efficacy of many of the animal and human vaccines developed well into the twentieth century [9].

### 1.2. Emergence of Regulated Vaccine Testing

As the value of vaccination gained credibility, medical and veterinary practitioners recognized the need to systematize the production and testing of vaccines in order to establish criteria indicative of safe and effective products. Regulation of vaccines was essentially absent until early in the twentieth century, at which point a series of adverse events caused by contaminated vaccines and other problems with human and animal immunological products prompted government authorities to develop strategies for assessing and controlling vaccines [10,11]. The U.S. Congress passed the first piece of legislation mandating the control of vaccine quality in 1902, later known as the Biologics Control Act, and further strengthened the role of government regulation of vaccines in the United States Public Service Act of 1944, ultimately granting this authority to the Center for Biologics Evaluation and Research (CBER) of the U.S. Food and Drug Administration (FDA). The Virus-Serum-Toxin Act of 1913 mandated similar controls over veterinary vaccines, which are now enforced by the Center for Veterinary Biologics (CVB) within the U.S. Department of Agriculture's (USDA) Animal and Plant Health Inspection Service (APHIS). With these pieces of legislation, federal agencies were charged with ensuring vaccine safety through licensure processes that required scientific assessment of vaccine products before they could be sold [9].

Vaccines' biological origin confers complexity and heterogeneity that, by definition, means that batches of the same product can vary qualitatively over time or between manufacturers [12,13]. Qualitative parameters—specified for U.S. veterinary markets within regulatory guidance as safety, purity, potency and efficacy as defined in standard requirements—are measured for each batch in order to determine the acceptability of the final product [13,14]. Potency, for example, is defined in Title 9 in the U.S. Code of Federal Regulations as a measure of the relative strength of a biological product as compared to a reference preparation that must be established for each batch of vaccine [15].

Batch potency testing regulations that aim to control this particular quality parameter have changed little since their introduction for many currently licensed vaccine products and continue to rely on the extensive use of live animals and challenge tests [12,16,17]. Potency regulations specify tests or types of tests that are considered acceptable for use by manufacturers in order to satisfy production standards for this aspect of vaccine quality. In the U.S., E.U. and elsewhere, batch potency testing regulations are addressed in monographs and guidelines issued by international and national regulatory authorities and pharmacopoeias (e.g., European Directorate for the Quality of Medicines and HealthCare and the European Pharmacopoeia; USDA), international organizations (e.g., World Health Organization; World Organisation for Animal Health), and other national and international research and evaluation bodies (e.g., International Cooperation on Harmonisation of Technical Requirements for Registration of Veterinary Medicinal Products, VICH; Interagency Coordinating Committee on the Validation of Alternative Methods, ICCVAM; European Centre for the Validation of Alternative Methods, ECVAM) that are also responsible for the development and review of techniques addressed in regulatory guidance [1].

The tests required or recommended in these texts are varied. Potency tests for many types of vaccines are carried out *in vitro* and do not require animals, but inactivated vaccines can require that large numbers of animals be used in challenge tests for this purpose. Of approximately ten million laboratory animals used each year in the production of vaccines and other biologics, eighty percent are used for routine quality control and batch testing of finished products [6]. In addition to the sheer numbers of animals used, many of the challenge tests in use today for older vaccines are “fraught with variability” and may not offer reliable precision or predictiveness [9]. Animal test methods for some vaccine potency assays do not uniformly agree on the potency ranking of the same product because of this inherent variability [18].

### 1.3. Non-Animal Quality Tests and the Slow Path to Their Implementation

The capacity to integrate new technologies into existing guidance has led to the development of increasing numbers of quality testing methods that reduce or eliminate reliance on animal-based tests. Many animal replacement and reduction strategies in potency testing incorporate either direct antigen quantitation in vaccine batches or quantitation of the immune response in immunized animals, measurements that have been made possible through the development of diverse *in vitro* techniques including enzyme-linked immunosorbent assays (ELISA), toxin-binding assays and other serological and analytical methods for the detection of specific antigens or antibodies [13]. Where cost comparisons are available, *in vitro* techniques are generally less costly than *in vivo* methods owing to the significant expense associated with purchasing, housing and managing animals in the laboratory [19,20].

In theory, regulations and guidance are updated based on technological advancements and greater insights into the biological basis of vaccine safety and efficacy that, by extension, lead to the increased use of humane methodologies. In practice, however, non-animal replacements for *in vivo* assays do not penetrate regulatory guidelines quickly or easily and their validation does not necessarily ensure their use as replacements for animal-based methods. Commentators have implicated a number of possible reasons for this lagging pace of alternative method use [21-23]. Regulators are seen as reluctant to implement non-animal methods because of the “serious responsibility regulators bear for the safety of the products they allow onto the market” and as a consequence tend to favor the *in vivo* methods that have historically been used to evaluate and minimize risk [21]. Manufacturers have indicated that comfort with existing animal tests—or difficulty meeting the requirements for use of refinement or replacement techniques—is at least partially responsible for incomplete implementation of replacement or refinement assays [21,24,25]. Furthermore, private industry is perceived as unwilling to share data on the development or use of non-animal methods or refinements that might otherwise hasten regulatory comfort with these more humane approaches [21]. Considering the globalized market for veterinary biologics, a lack of both data sharing and international harmonization among regulators compounds the approval process for manufacturers that must duplicate testing in order to satisfy regional marketing requirements [13,21]. For veterinary vaccines, considerable differences in the acceptance of alternative methods between the U.S. and E.U. ensure that traditional *in vivo* tests remain in use [21]. The net result of these obstacles is that implementation of approved non-animal or refined methods as improved adjuncts to or as complete replacements for animal methods has been slow and irregular [1,13,17]. Examples presented below highlight instances of slow uptake of new methods that reduce or eliminate the use of animals and also bring to light the need for oversight to ensure compliance and the removal of barriers such as application fees and guidance for obsolete methods in order to reduce animal testing.

While novel vaccine production strategies are emerging that emphasize consistency of production rather than compulsory animal-based quality testing of each batch of final product, the problem remains that currently available non-animal approaches have not reached a point of wide use by vaccine manufacturers and acceptance by regulatory bodies. This lagging pace of implementation has become a focal point for stakeholders involved in all aspects of vaccine development and testing, including academic researchers, vaccine industries, animal protection organizations and regulatory authorities [13,26]. As interest and involvement in vaccine testing extends beyond the laboratories and regulatory offices involved in the development and validation of replacement and refinement techniques into the realm of public interest, we have worked on a case-by-case basis as specific techniques have been validated to provide an additional impetus for the broader application of strategies that reduce the burden of animals in experimentation.

## 2. Paradigm

To systematically address the slow pace of broadening the use of available non-animal techniques in veterinary vaccine potency testing, we developed a qualitative approach that defines and addresses barriers within industry and government as quickly as possible using publicly available information. From our interactions with regulatory authorities and veterinary vaccine manufacturers, as well as with organizations that interact with and have access to privileged or confidential business information (CBI) from these groups, we identified key steps that have resolved or partially addressed the underutilization of available non-animal methods by promoting and verifying their uptake.

We have synthesized this process into a generalized ‘bridging paradigm’ composed of a set of practices that can be followed as a model in order to identify barriers to implementation, to establish necessary next steps to ensure usage, to describe critical data needs and, ultimately, to confirm the use of available non-animal vaccine potency assays and the retirement of traditional animal-based methods. Such an approach bridges the gap between the validation of non-animal methods and their adoption in place of animal tests in regulatory and industrial settings while emphasizing the need for public accountability and transparency in this context and, especially, in instances where implementation has been incomplete or otherwise complicated by ineffective policies and practices.

**Figure 1:**
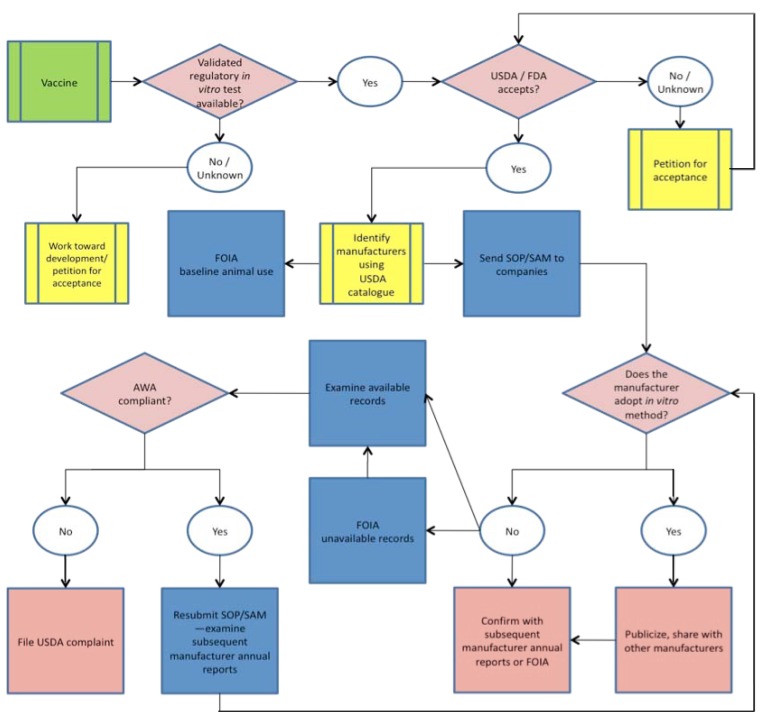
Generalized bridging paradigm for information-gathering and action.

This paradigm can be visualized as an information collection and dissemination matrix that is customized to the needs of each vaccine for which a non-animal potency test exists (Figure 1). In each case, information collection and confirmation of regulatory acceptance of non-animal approaches are necessary prerequisites for identifying the essential next steps in the process. In some cases, this approach identifies instances of possible non-compliance with the Animal Welfare Act or other regulations. In all cases, validation data and standard operating procedures (SOP) or supplemental assay methods (SAM) for non-animal methods are supplied to regulators and manufacturers, followed by efforts to confirm acceptance by regulators and implementation in industry. Considering the case-by-case basis of this approach, we have opted to reduce our specific descriptions of the processes underlying these refinement or replacement assays as this information can be found in referenced material. Detailed descriptions of this process are provided in the context of our experiences with specific veterinary vaccines below.

## 3. Cases

Each case, expanded upon below, illustrates how the bridging paradigm is used to increase the use of available, non-animal-based methods or to decrease the use of older *in vivo* methods that have been functionally replaced but have not yet been deleted by regulatory agencies or abandoned by industry in favor of an improved method. While there are numerous reasons for a lack of complete implementation of newly validated or approved methods, we focus on only a subset in order to illustrate the capacity of our paradigm to identify and resolve implementation issues. Each case study highlights unique situational obstacles that have been or are in the process of being overcome.

### 3.1. Erysipelas Vaccine

In some applications, our approach has successfully prompted the withdrawal of tests using animals from codified requirements and official methods of analysis. This was our experience with efforts to promote non-animal *Erysipelothrix rhusiopathiae* vaccine batch potency tests.

**Figure 2:**
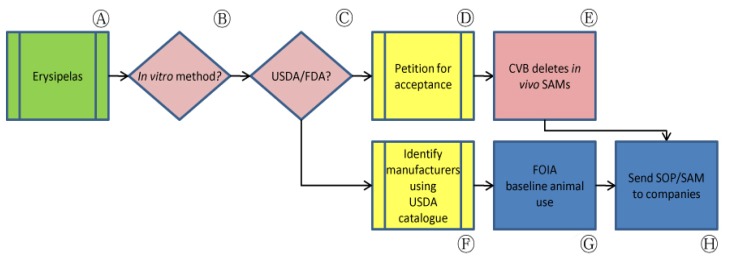
Application of the bridging paradigm to erysipelas vaccine potency testing in the United States.

The U.S. standard requirements for *E. rhusiopathiae* biological products, under the aegis of USDA's APHIS, have remained virtually unchanged for almost thirty years. These standards require challenge procedures (Figure 2, Ⓐ) that are codified in Title 9 of the Code of Federal Regulations (CFR) sections 113.67 and 113.119 and expanded or modified in CVB SAM 605, 606, 611, 612 and 613 (Table 1). Procedures presented in these standard requirements, by definition, often involve hundreds of animals and can cause severe suffering in the animals used. In accordance with 9 CFR §113.67, for example, evidence of a satisfactory challenge in unvaccinated pigs includes “acute illness with hyperemia of the abdomen and ears, possibly terminating in sudden death; moribundity, with or without metastatic skin lesions; depression with anorexia, stiffness and/or joint involvement; or any combination of these symptoms and lesions.” A similar challenge assay described in SAM 611 requires 160 mice and may require repetition in order to satisfy the required statistical limits.

In contrast, two newer *in vitro* potency tests allowed by USDA avoid the use of animals entirely (Figure 2, Ⓑ). As novel methods are developed that satisfy regulatory data requirements without reliance on older methods established in the CFR, CVB publishes these methods as SAMs and defines the CFR-based standard testing requirements that the newer method can replace. SAM 612, for example, outlines potency evaluation of live vaccines based on the growth of colony-forming units in culture in place of the challenge test described in 9 CFR §113.67. For bacterins, SAM 613 allows the use of an *in vitro* ELISA-based antigen quantitation method. Nevertheless, USDA approval of these non-animal approaches had not been accompanied by a concurrent deletion of USDA's animal based standard requirements (Figure 2, Ⓒ).

In order to increase the availability of non-animal potency methods in the U.S., we petitioned for USDA acceptance of an additional internationally available serological assay while concurrently requesting the deletion of *in vivo* assays that would be replaced by this measure, considering that the *in vitro* SAMs had already been approved for this purpose (Figure 2, Ⓒ). In 2002, the European Center for the Validation of Alternative Methods (ECVAM) Scientific Advisory Committee (ESAC) endorsed the use of a serological ELISA method as a validated procedure for measuring the potency of inactivated swine erysipelas vaccines. This method was subsequently added to the monographic *E. rhusiopathiae* testing requirements of the European Pharmacopoeia (Ph. Eur.). Although serological assays require animals, they nevertheless reduce the number of animals used while concurrently lessening the pain and suffering associated with *in vivo* challenge assays.

We submitted a request to USDA's Center for Veterinary Biologics (CVB) for the Agency's opinion on the acceptability of data from the *in vitro* potency assay in place of the *in vivo* assay, noting that the Ph. Eur. directly states that a vaccination-challenge test for each batch of inactivated *E. rhusiopathiae* vaccine is not necessary [27]. In response, CVB permanently deleted *in vivo* SAMs 605 and 606 (Figure 2, Ⓔ) [25,28]. Although CVB maintained in its response that the serological potency assay allowed by Ph. Eur. was not currently compatible for use with U.S. vaccines, the Center noted that it had initiated work to reduce the number of animals required to qualify reference bacterins that enable the use of *in vitro* SAM 613. While the *in vivo* SAM 611 remains authorized, CVB noted that, because “nearly all new methods submitted by firms are *in vitro* assays, it is expected that (the remaining *in vivo* assay) will be obsolete in the near future” [25].

During our correspondence with CVB, we sought publicly available records for companies involved in the manufacture of *E. rhusiopathiae* vaccines licensed in the U.S. to search for evidence of implementation of the available *in vitro* methods. After consulting USDA's Product Code Book, we collected Annual Reports of Facility (APHIS Form 7023) documenting animal use by species for six facilities licensed to market erysipelas vaccines (Figure 2, Ⓕ). These reports are often publicly available at the APHIS website, although their availability is inconsistent and in some cases do not provided the information needed on specific animal tests. Animal use from the mouse vaccination-challenge assay described in SAM 611, for example, is functionally untraceable through Annual Reports of Facility as mice are not regulated species under the Animal Welfare Act (AWA).

We supplemented this information with other publicly accessible information obtained from manufacturers' outlines of production for their products. We submitted requests under the Freedom of Information Act (FOIA) for lot release protocols for these products in order to establish baseline animal use for specific purposes and species for comparison between dates prior to and following changes in USDA policy regarding available non-animal potency tests (Figure 2, Ⓖ). The FOIA request process is not simple and frequently takes months or years from date of request submission to date of information receipt. Additionally, not all manufacturers respond to USDA's requests for this information during the recommended timeframe. While information obtained through this process is not always clear, we were nevertheless able to use the resulting records to identify companies that have recorded the use of animal-based potency assays. In some cases, the most current lot release protocol may be several years old at the time of receipt. We are currently analyzing information that we have received through the FOIA request process, and are preparing to contact these companies with the information at hand to clarify their potency testing processes and additionally to disseminate information on CVB-accepted non-animal methods (Figure 2, Ⓗ). As this process continues, we aim to track data from accumulated Annual Reports of Facility and outlines of production to identify changes in animal use that may indicate increased use of non-animal potency tests.

### 3.2. Leptospirosis Vaccine

Routine assessment of other veterinary vaccine potency tests using this paradigm has uncovered potential Animal Welfare Act (AWA) violations related to requirements that manufacturers document an annual search for available non-animal assays that may replace the need for animal-based potency tests. These experiences, as with *Leptospira interrogans* vaccine manufacturers, can lead to formal complaints submitted to regulatory authorities to highlight opportunities to adopt humane techniques.

**Figure 3:**
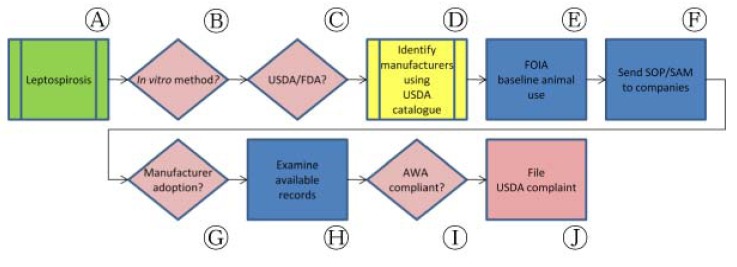
Application of bridging paradigm to leptospirosis vaccine potency testing in the United States.

Much like *E. rhusiopathiae* biological products, the USDA standard requirements for measuring potency of *Leptospira interrogans* bacterins—of which there are multiple serovars—have changed little in more than thirty years (Figure 3, Ⓐ). As described in 9 CFR §113.101, §113.102 and §113.104, these tests require that at least forty hamsters be injected with virulent *L. interrogans* cultures in challenge tests. As an alternative to the challenge approach, CVB has approved the use of non-animal procedures to calculate potency of four serovars relative to qualified reference bacterins (Figure 3, Ⓑ). In SAMs 624, 625, 626 and 627, CVB outlines the use of ELISA-based potency testing methods for serovars *pomona, canicola, grippotyphosa* and *icterohaemorrhagiae*, respectively (Figure 3, Ⓒ).

Confirming implementation of methods described in these SAMs in industry has been difficult. In 2010, we submitted FOIA requests for the lot release protocols for applicable *L. interrogans* bacterin products from U.S. manufacturers in addition to reviewing publicly available USDA Annual Reports of Facility (Figure 3, Ⓓ, Ⓔ). We wrote to manufacturers on repeated occasions between 2007 and 2011 announcing the availability of these methods, but received only one direct response—from a firm that had been directly involved in the development of these assays—confirming a move toward the use of ELISA in place of hamsters (Figure 3, Ⓕ, Ⓖ).

Review of these public documents revealed limitations of the information collected by regulatory authorities (Figure 3, Ⓗ). In one case, we identified a possible AWA violation related to the Institutional Animal Care and Use Committee (IACUC) review and approval of an animal method despite the availability of SAMs 624—627 (Figure 3, Ⓘ). In this example, a manufacturer's Annual Reports of Facility revealed that the IACUC had repeatedly resubmitted outdated information regarding the availability of a non-animal replacement for the use of animals—in direct conflict with AWA's requirement that an IACUC demonstrate a “reasonable and good faith effort” to search for alternatives [29]. Repeated attempts to contact the manufacturer regarding this issue failed and we ultimately submitted a USDA complaint in order to have the matter investigated by APHIS officials (Figure 3, Ⓙ). Results of this complaint demonstrated that the company in question had failed to qualify its vaccine master seed cultures, a required step in the process of securing CVB approval to use the *in vitro* methods outlined in SAMs 624—627 [30]. USDA noted that the company intends to resubmit new master seed cultures in the near future, at which point similar record examination will be necessary.

### 3.3. Non-Tetanus Clostridial Vaccines and Ascites

**Figure 4:**
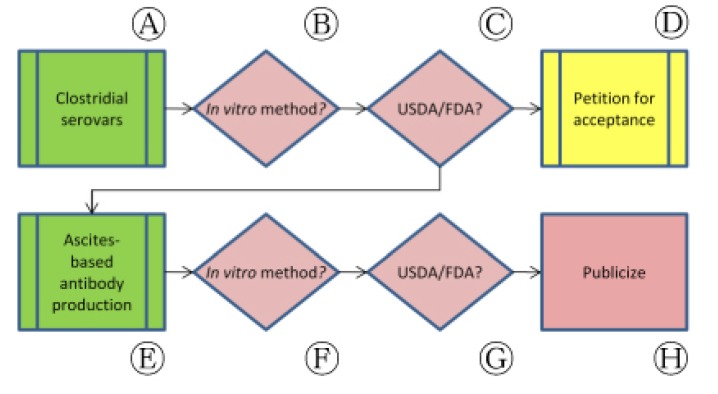
Application of bridging paradigm to clostridial vaccine potency testing and monoclonal antibody production for *in vitro* potency assays in the United States.

Correspondence with regulatory authorities in this bridging approach also identified unanticipated opportunities to address cases of animal use in vaccine-related processes other than potency testing that may also be replaceable. As we sought clarification on clostridial vaccine potency testing (Figure 4, Ⓐ), for example, the use of animal-based techniques used to produce *in vitro* test reagents was identified as an equally pressing issue.

Following collaborative studies published in 2003, monographs for two clostridial vaccines (*Clostridium novyi* and *C. perfringens*) were updated in Ph. Eur. to allow the use of non-animal antigen quantitation in place of *in vivo* potency tests (Figure 4, Ⓑ). We wrote to CVB in October 2009 to ask for the Center's position on the use of similar *in vitro* approaches for vaccines representing all clostridial serovars (Figure 4, Ⓒ). CVB responded in December 2009 by informing us that the Center had begun laying the groundwork for *in vitro* testing of clostridial antigens and, as a part of that effort, had begun drafting a SAM (220) for one serovar, *C. chauveoi* [31]. In this response, however, CVB also noted that an existing SAM describing an *in vivo* potency assay cannot be declared obsolete as long as a licensed product is still being manufactured and tested using animal-based methods. No timeline for the release of the draft SAM 220 has been provided, but CVB has noted that the draft is undergoing review and will be posted online once this process is complete (Figure 4, Ⓓ) [32].

At CVB's disclosure that the Center was working toward an ELISA-based *C. chauveoi* potency test, we sought information on how animals will be used in development of the non-animal replacement for the *in vivo* potency assay (Figure 4, Ⓔ). The use of *in vitro* assays, including ELISA-based methods, can be accompanied by the use of animals in order to develop reagents that are essential to the test process. ELISA, as an example, requires the use of monoclonal antibodies that recognize a specific antigen. In Veterinary Services memo 800.97, CVB outlines the availability and procurement procedures for test reagents and other components that are used in test systems set forth in standards requirements or SAMs. While none of the available antibody preparations used in measuring *C. chauvoei* potency were indicated as having been prepared *in vivo*, 13 of 68 reagents listed were described as having been produced in ascites.

Our concern upon learning of the use of *in vivo* ascites-based antibodies was due to the established animal welfare issues surrounding this method and the fact that there are valid methods for antibody production that avoid animal use (Figure 4, Ⓕ) [33]. The ascites method of antibody production uses mice to generate and produce monoclonal antibodies (mAbs) in a lengthy and painful multistep process.

Due to the pain and stress caused by this method, the use of *in vivo* ascites mAb amplification has been banned or restricted in Australia, Germany, Switzerland, the Netherlands, and the United Kingdom and is discouraged by the U.S. National Institutes of Health and USDA. We sought clarification regarding ascites antibody use in a March 2010 letter to CVB. With reference to VS memo 800.97, we inquired whether only these 13 preparations were sourced from ascites and if, in the future, CVB intended to replace the use of ascites with non-animal bioreactor-based systems or other processes. In a June 2010 response, CVB confirmed that the majority of its mAb stocks are produced in bioreactors and most of its ascites-produced mAbs are from existing stockpiles (Figure 4, Ⓖ) [32]. Once these stocks have been depleted, CVB has noted that they will most likely reproduce stocks using bioreactor technologies, although some may continue to be produced using ascites for a time if they are difficult to transition into bioreactor production regimes (Figure 4, Ⓗ).

### 3.4. Newcastle Disease Virus Vaccine

**Figure 5:**
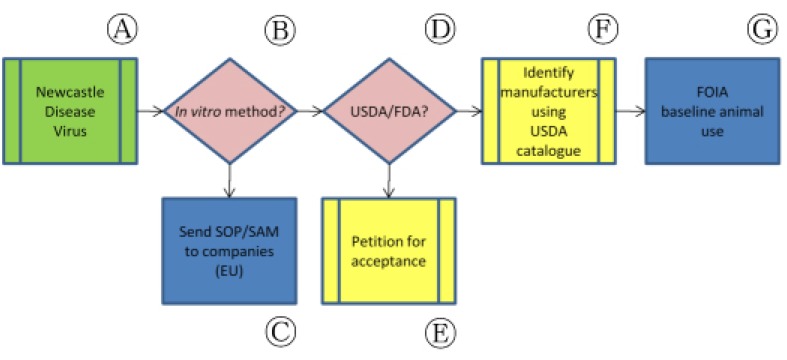
Application of bridging paradigm to NDV vaccine potency testing in the United States.

In some instances, internationally validated non-animal methods do not translate easily among regulatory authorities. As an illustration, Newcastle Disease Virus (NDV) vaccines can be tested *in vitro* in the E.U. but the vaccination-challenge assay in chickens remains the codified potency test in the U.S (Figure 5, Ⓐ).

Following the successful completion of a large-scale collaborative validation study in 2004, Ph. Eur. elected to include a non-animal ELISA-based antigen quantitation assay as a potency testing method in its monograph on inactivated NDV vaccines (Figure 5, Ⓑ). Although this *in vitro* assay was not accompanied by the deletion of the *in vivo* challenge assay already authorized in the monograph, study authors noted that the ELISA approach could nevertheless be regarded as technically superior to the older, more variable *in vivo* procedures.

In accordance with our paradigm, our first action was to ensure that manufacturers under the Ph. Eur.'s jurisdiction were aware of this monographic revision and had access to the appropriate information (Figure 5, Ⓒ). We sent copies of the ELISA standard operating procedure to European-based NDV vaccine manufacturers and asked that they implement the non-animal process in place of the *in vivo* challenge assay. In some cases, manufacturers had directly participated in the collaborative study but did not provide additional information on their plans to replace the use of *in vivo* assays with the non-animal potency test or a potential timelines for replacement.

We also sought to establish the acceptability of data from ELISA-based potency assays for NDV vaccines licensed by USDA's CVB for distribution in the U.S. (Figure 5, Ⓓ). Because the codified NDV potency assay in the U.S. requires a vaccination-challenge potency assay, we encouraged CVB to host a similar collaborative study among U.S.-licensed NDV vaccine manufacturers (Figure 5, Ⓔ). In response, CVB asserted that U.S. vaccines had been found incompatible with *in vitro* NDV vaccine potency tests despite their approved use elsewhere. Although CVB permits individual manufacturers to develop and submit non-animal assays that can be used in place of *in vivo* regulatory standards, the Center has not committed to a U.S.-based validation of the E.U.-approved non-animal potency assay. Commenting on international testing differences for similar products, CVB noted that “husbandry practices, nature of vaccine strains and nature of challenge strains may differ between the U.S. and other countries, and all this must be factored into changes in testing or the interpretation of test methods” [34]. As a result, “a suitable replacement [for the *in vivo* NDV vaccine potency assay] has not been identified.”

The potential for increased animal use caused by international differences in the regulation of NDV vaccine products is compounded by the multinational status of several vaccine manufacturers. Fort Dodge Animal Health, for example, participated in the E.U.-based collaborative validation study, yet also holds USDA licenses for NDV vaccine products distributed in the U.S. [35]. Considering this overlap, and the lack of transparency from manufacturers themselves, it is uncertain whether similar products produced by a single manufacturer are subjected to repeated or redundant potency assays depending on the regulatory jurisdiction of their ultimate destination.

We submitted FOIA requests for lot release protocols and outlines of production for NDV vaccine products licensed by USDA in addition to compiling publicly available USDA Annual Reports of Facility for the companies responsible for the manufacture and quality testing of these products (Figure 5, Ⓕ, Ⓖ). While these documents can directly indicate the implementation of an accepted non-animal potency test (e.g., an outline of production indicates the use of an ELISA rather than a codified challenge assay), in most cases companies are legally permitted to selectively redact such information. Alternately, analysis of several years' animal use numbers from Annual Reports of Facility may indicate a drop in animal use during a particular year that, if sustained, may indicate implementation of an available non-animal method. These reports are useless in gauging implementation of non-animal NDV vaccine potency tests, however, because birds are specifically excluded from the Animal Welfare Act (AWA) and their use is therefore not reported by testing facilities. In addition, as noted previously, the FOIA request process does not operate on a predictable or timely schedule, and manufacturers do not consistently reply to USDA's requests for data. Of five FOIA requests submitted regarding NDV vaccine manufacturers since March 2010, the results of only two have been received.

### 3.5. Target Animal Batch Safety Testing for Veterinary Vaccines

The target animal batch safety test (TABST) is a safety test performed on each batch of veterinary vaccines manufactured in the United States but, since 2002, this test can be waived in the E.U. after batch consistency is demonstrated [36]. These tests are performed for veterinary vaccines on the intended target animal (e.g., a vaccine manufactured for dogs would be tested on dogs) to insure that no overt adverse changes occur in healthy animals and to prove that the current batch is safe for clinical use. These tests use up to 10 times the recommended dose (for live attenuated vaccines) and twice the normal dose for inactivated vaccines and can last up to 28 days.

**Figure 6:**
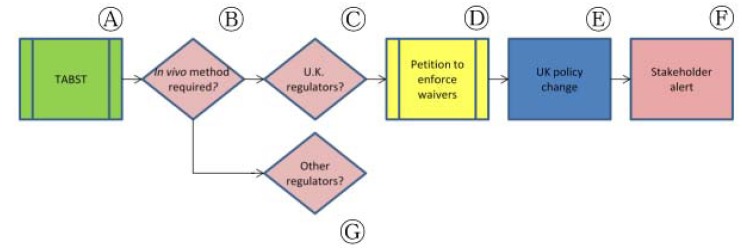
Application of bridging paradigm to TABST in the United Kingdom and additional jurisdictions

The necessity of the TABST was called into question to such a degree in the 1990s that the European Centre for the Validation of Alternative Methods (ECVAM) took up the issue (Figure 6, Ⓐ). In 1997, ECVAM convened the Advisory Group on Alternatives to Animal Testing in Immunobiologicals to perform a study on the relevance of TABST [37]. Results of this study included data from official medicines control laboratories (OMCLs) within the European Union as well as from private industry. Throughout the period of 1994 to 1997, OMCLs tested 11,185 vaccine batches and fourteen manufacturers submitted data from 11,386 batches for the period between 1997 and 1999.

The study concluded that TABST as a routine part of batch testing was no longer relevant to insure safety due to the increased rigor and quality control introduced by Good Laboratory Practice and Good Manufacturing Practice (Figure 6, Ⓑ). The advisory group recommended that TABST be omitted from routine testing requirements in most every case (except for new products or for vaccines that had been recently licensed so that batch safety could be established).

The advisory group went on to recommend that the TABST requirement should be immediately deleted from Ph. Eur. monographs (except in specific cases where it is considered necessary) and that the Committee for Veterinary Medicinal Products (CVMP) of the European Medicines Agency (EMA) revise its guidance for immunobiologicals to reflect this recommended change. Harmonization between Ph. Eur. and E.U. guidance was also a priority in the 2002 “Statement of the Relevance of the Target Animal Safety Test for Batch Safety Testing of Vaccines for Veterinary Use” published by ECVAM [36].

In 2008, we examined ECVAM's recommendation to waive the TABST for those companies that had previously proven batch safety. We began researching whether the waiver system had been taken up by industry and whether there was any oversight to ensure that companies were not conducting TABSTs when they were not required. We corresponded with, *inter alia*, the Home Office (H.O.) in the United Kingdom (U.K.)—the government department responsible for approving all scientific procedures using animals—to ascertain the extent of uptake by local industry and to find out how the U.K. government oversaw the waiver system (Figure 6, Ⓒ). Because E.U. legislation requires that scientific procedures using animals “shall not be performed if another scientifically satisfactory method of obtaining the result sought, not entailing the use of an animal, is reasonably and practicably available” (European Council Directive 86/609/EEC, subsequently replaced by Directive 2010/63/EU), our request for information was buoyed by the legal requirement to avoid animal tests whenever possible.

We found that the H.O. was not tracking the use of the use of the waiver system for individual products. In fact, at the time (2008), the U.K. regulator of veterinary medicines, the Veterinary Medicines Directorate (VMD), was requiring that industry pay a fee in order to apply for the waiver. We pursued this matter with ministers and officials—including corresponding and holding two meetings with the H.O. After our initial requests, the ministry supervising VMD agreed it would stop charging fees for the application to waive the TABST (Figure 6, Ⓓ) [38,39]. However, as the regulator responsible for ensuring compliance with the E.U. legal obligation not to use animal methods where other approaches are available, the H.O. needed to introduce a mechanism to ensure that waivers were being implemented wherever possible. The development of a compliance program for the U.K. was an 18-month-long process, but the H.O. will now monitor the performance of TABST and will no longer approve the tests when they are not required by law (Figure 6, Ⓔ) [40,41]. At the culminating stage of this effort in the U.K., we sent letters to vaccine manufacturers alerting them of the policy change and requested that the H.O. do the same (Figure 6, Ⓕ). Currently, a similar process is being followed with respect to the European Commission (so that each member state has a similar process of oversight and full implementation), USDA and VICH (Figure 6, Ⓖ).

## 4. Conclusions and Recommendations

Without focusing on the development or validation of novel reduction or refinement techniques, the bridging paradigm successfully expanded implementation of available non-animal methods for U.S. batch potency testing of several veterinary vaccines while also highlighting and resolving examples of vaccine-related animal tests that can be reduced in number or replaced with other means in the U.S. and the U.K. Application of this paradigm demonstrably succeeded—from the elimination of barriers to exemptions from avoidable TABST for all veterinary vaccines to the deletion of guidance involving animal-based tests—but its value included the identification of implementation barriers otherwise not addressed by any other regulatory-industrial cooperations that are otherwise driving forces in the promotion of non-animal potency tests. Throughout the development and refinement of the bridging paradigm we observed that no other entity or process was established to provide this function. In addition to the abundance of regulations and guidance in which information regarding test method availability and even acceptance by regulatory agencies can be lost, we found that there were specific roadblocks that inhibited the flow of information and the modernization of vaccine potency testing methods which, relative to the cases presented here, are described below.

Barriers to implementation included a lack of oversight even when there was a legal requirement to avoid animal testing whenever possible. Without specific government oversight, industry and regulators were often prone to continue with familiar protocols. This was apparent in our experience with TABST, until our bridging process resulted in a change in regulatory structure. The tendency to cling to familiar protocols, or difficulty in demonstrating technical ability to adopt approved non-animal protocols, was also an issue with the continued use of hamsters in lieu of the ELISA-based test for insuring potency of the *Leptospirosis* vaccines in at least one instance.

With respect to issues of transparency (or lack thereof) and its effects on our ability to collect the information needed to assess compliance with Directive 86/609/EEC, the AWA, or to verify the voluntary uptake of new methods by industry, we were met with major limitations. Obtaining information on veterinary vaccine testing through FOIA requests is unpredictable, even with respect to aspects of quality testing that are federally mandated, and the irregular interpretation of CBI results in inconsistent treatment of information that can reliably be considered public. Even when requested to do so by USDA, companies do not consistently provide records on the timetable recommended and, in some cases, these requests must be made several times before any material is provided to USDA for review in a process that can take more than a year [42,43]. Because the vast majority of animals used in vaccine testing—rats and mice—are not covered under the AWA, it is nearly impossible to track changes in animal use involving these animals and we therefore have to rely upon industry's willingness to communicate their use of newer methods. Without reliable information reflecting the numbers and species of animals used for vaccine potency tests, assessments of the costs and purported benefits of animal-based methods is compromised. These problems were obstacles in almost every case outlined above and should be taken into consideration by regulatory authorities in the future.

In order to decrease the use of animal-based methods when there is an available non-animal-based alternative, we have petitioned for the deletion of old methods and requested that agencies send out notices regarding novel method acceptance. In many cases we have taken on the notification process ourselves. We have found that the process of method retirement is not standardized and reducing the use of animal-based methods is often random. We have even been met with resistance when we have requested method deletion, in one case simply due to the claimed administrative burden that deletion would impose upon the agency. Despite these obstacles, we verifiably increased the use of novel *in vitro* methods while also making it clearer to stakeholders that older animal-based methods could be retired.

By engaging with regulators and manufacturers, we have effectively promoted non-animal or refined approaches to vaccine batch potency testing. This process establishes the acceptability of data from novel methods by regulatory authorities, distributes information on available and accepted non-animal approaches via stakeholder alerts, involves trade press in publicizing accepted non-animal techniques, and confirms manufacturer implementation of these methods. Despite a lack of transparency in the process of non-animal test method approval in the U.S., we have shown that petitioning for regulatory acceptance of internationally validated methods can hasten the approval of existing non-animal methods or, conversely, the removal an obsolete *in vivo* method from use.

This process has also underscored the fact that, currently, the burden of ensuring that validated non-animal methods are taken up in the regulatory and industrial arena lies with interested parties that are not necessarily directly involved in regulation or in manufacturing. Despite the fact that both the U.S. and the E.U. have specific coordination efforts for the validation of newer testing methods, neither ECVAM nor ICCVAM have a mandate to audit the uptake of novel methods. Although mechanisms are theoretically in place to incorporate new approaches, the cases presented here suggest that these mechanisms are incomplete and cannot be relied upon to promote replacement of animal-based testing protocols in a timely and proactive manner. Our experience indicates—and the preceding examples emphasize—that changes made to regulatory guidance and oversight due to the application of the bridging paradigm would not have taken place otherwise. Until international regulators are able to demonstrate that their approval of non-animal tests results in the active implementation of those methods, we will continue to apply this bridging paradigm for these and other veterinary and human vaccines.

## Figures and Tables

**Table 1:** Status of USDA Supplemental Assay Methods for *E. rhusiopathiae* biological products.

**SAM**	**Description**	**Status**
605	*In vivo* potency testing of erysipelas bacterins in swine	Deleted
606	*In vivo* potency testing of erysipelas vaccines in swine	Deleted
611	*In vivo* potency testing of erysipelas bacterins in mice	Active
612	*In vitro* potency testing (bacterial plate count) of *E. rhusiopathiae* vaccines	Active
613	*In vitro* potency testing of *E. rhusiopathiae* bacterins	Active

## Supplementary Files

Supplementary File 1
