# Supplementary material for: Bridging the Gap Between Validation and Implementation of Non-Animal Veterinary Vaccine Potency Testing Methods
Source: Animals (Basel). 2011 Nov 29;1(4):414–32. doi: 10.3390/ani1040414 (PMC4513470; doi:10.3390/ani1040414)
Supplement: Supplementary File 1 [file animals-01-00414-s001.zip › supplementary materials/24 CVB erysipelas.pdf]

USDA

September 25, 2009

United States Department of Agriculture

Animal and Plant Health Inspection Service

Veterinary Services

Center for Veterinary Biologics

1920 Dayton Avenue
P.O. Box 844
Ames, IA 50010

(515) 337-6100

Mr. Jeffrey Brown
Regulatory Testing Division
People for Ethical Treatment of Animals (PETA)
501 Front Street
Norfolk, VA 23510

Dear Mr. Brown:

This is in response to your letter dated August 19, 2009, regarding the application of an *in vitro* potency test for *Erysipelas rhusiopathiae* biologics. We appreciate your interest in our biologics program and welcome the opportunity to describe our work in the development of alternatives to animal usage in the potency testing of veterinary biologics.

The Center for Veterinary Biologics (CVB) advocates and supports the development and implementation of *in vitro* methods for potency testing of veterinary biologics to reduce the use of animals in routine batch testing. This commitment was recently demonstrated when the CVB issued Notice Number 09-20 on August 31, 2009, entitled "Withdrawal of Supplemental Assay Methods 601 (Potency Testing of Erysipelas Antiserum in Mice), 605 (Potency Testing of Erysipelas Bacterins in Swine), and 606 (Potency Testing of Erysipelas Vaccines in Swine)". Elimination of these three *in vivo* test methods promotes the use of established *in vitro* methods currently outlined in Supplemental Assay Methods (SAMs) 612 and 613.

SAM 612, "Supplemental Assay Method for Bacterial Plate Count of *Erysipelothrix rhusiopathiae* Vaccines", describes an *in vitro* method for determining the potency of live erysipelas vaccines by bacterial plate count. SAM 613, "Supplemental Assay Method for *In vitro* Potency Testing of *Erysipelothrix rhusiopathiae* Bacterins", describes an *in vitro* method for determining the potency of inactivated erysipelas bacterins by enzyme-linked immunosorbent assay (ELISA). The procedure outlined in SAM 613 was developed and implemented by the CVB in 1993 (Henderson, *et al.*, 1993a), and is widely used by veterinary biologics firms for batch potency testing, as well as the CVB for regulatory testing. This ELISA method utilizes a monoclonal antibody and a monospecific polyclonal antibody in tandem to quantify the major protective antigen, 64-66 kilodalton (kDa) protein of *E. rhusiopathiae* (Henderson, *et al.*, 1993b).

Currently, one *in vivo* test method is available for testing the potency of inactivated erysipelas bacterins. The assay, outlined in SAM 611 "Supplemental Assay Method for Potency Testing of Erysipelas Bacterins in Mice", is the *in vivo* method previously described in the European Pharmacopoeia (monograph 0064) for batch potency testing of inactivated erysipelas bacterins. The CVB is committed to the 3R's concept to refine, replace, and reduce the use of animals in routine testing, and in regard to *Erysipelothrix* bacterins specifically; the CVB recommends the use of SAM 613 for potency testing.

However, biologics firms have the right to develop and validate their own potency tests for their products which are subject to regulatory review prior to implementation. Because nearly all new test methods submitted by firms are *in vitro* assays, it is expected that SAM 611 will be obsolete in the near future.

The CVB is actively developing new *in vitro* methods and reagents for testing veterinary biologics. Currently, the CVB is working on an ELISA-based serological test utilizing the major protective 64-66 kDa protein of *E. rhusiopathiae* to indirectly measure the potency of erysipelas bacterins in experimentally vaccinated swine. If a positive correlation between antibody titers and protection is found, this procedure may eliminate use of the challenge test that is currently used to qualify erysipelas reference bacterins. In terms of reagents, the CVB produces several monoclonal antibodies by bioreactor system to replace ascites production in mice. Currently, an anti-*E. rhusiopathiae* 64-66 kDa protein monoclonal antibody, produced in the bioreactor system, is being evaluated for use in SAM 613.

The CVB supports the use of the internationally-validated serological test for routine potency testing of inactivated swine erysipelas bacterins as recommended by the European Centre for the Validation of Alternative Methods, and efforts are being made to evaluate its use with products designed for the United States market. As with any new testing protocol, there are particular issues with this assay that must be investigated prior to implementation. In particular, it must be determined whether the coating antigen, developed from the German reference strain Frankfurt XI (serotype N), is completely recognized by antibodies produced against *E. rhusiopathiae* strains of serotypes 1 and 2 used in U.S. erysipelas bacterins. The article by Rosskopf-Steicher, *et al.*, (2001) specifically states that "the vaccines [tested] were representative for the spectrum of products on the German market", and it is unclear whether U.S. vaccines can be accurately evaluated with the coating antigen used in the protocol. It has been shown that the 64-66 kDa protective protein (encoded by the *spa* gene) of *E. rhusiopathiae* has an amino acid sequence that can vary from strain to strain which can negatively affect cross-protectiveness among strains possessing an alternate sequence (To and Nagai, 2007). The CVB has conducted its own research regarding this topic, and our results substantiate this finding; therefore, it is important to thoroughly evaluate the European serological test against products designed for the U.S. market before it can be implemented as an official regulatory test.

Another aspect that requires consideration is the fact that the serological ELISA does not measure the effects of the cell-mediated immune (CMI) response. The CMI response is believed to play a role in immunity, because erysipelas is a systemic disease and the causative organism can survive intracellularly (Shimoji, *et al.*, 1996; Franz, *et al.*, 1995). Some erysipelas bacterins may be tested by *in vivo* method to account for the CMI response which is not measured in assays that only assess antibody titers. Although a correlation between mouse protection and antibody titers against the 64-66 kDa antigen from vaccinated mice has been reported (Imada,

et al., 2003), results are inconsistent among laboratories (Eames, *et al.*, 2006 and CVB studies, 2008) and are not fully accepted. Additionally, it is important to realize that ELISA titers do not distinguish between protective and non-protective antibodies which may cause misleading results. From this perspective, an *in vitro* antigen quantitation assay, as outlined in SAM 613, may be a superior method of determining erysipelas bacterin potency.

The CVB recognizes the interests of our many stakeholders, and we take very seriously our role of assuring quality veterinary biologics. Animal care and use is an extremely important aspect of meeting our mission, and your concerns are recognized. Although at this time we are unable to agree to adopt the European ELISA-based erysipelas bacterin potency test into our regulatory protocol, please be assured that attention is being given to this important topic.

References:

Eames GJ, Chin JC, Turner B, Barchia I. Evaluation of *Erysipelothrix rhusiopathiae* vaccines in pigs by intradermal challenge and immune responses. Veterinary Microbiology 116: 138-148. 2006.

Franz B, Davies ME, Horner A. Localization of viable bacteria and bacterial antigens in arthritic joints of *Erysipelothrix rhusiopathiae*-infected pigs. FEMS Immunology and Medical Microbiology 12:137-142. 1995.

Henderson LM, Scheevel KF, Walden DM. An enzyme-linked immunosorbent assay (ELISA) for potency testing of *Erysipelothrix rhusiopathiae* bacterins based on a protective monoclonal antibody. Iowa State University, Ames, Iowa. Doctoral thesis. 1993a.

Henderson LM, Jenkins PS, Scheevel KF, Walden DM. A monoclonal antibody specific for a putative protective immunogen of *Erysipelothrix rhusiopathiae*. Iowa State University, Ames, Iowa. Doctoral thesis. 1993b.

Imada Y, Mori Y, Daizoh M, Kudoh K, Sakano T. Enzyme-linked immunosorbent assay employing a recombinant antigen for detection of protective antibody against swine erysipelas. Journal of Clinical Microbiology 41:5015-5021. 2003.

Rosskopf-Streicher U, Johannes S, Wilhelm M, Cussler K. Quality control of inactivated erysipelas vaccines: results of an international collaborative study to establish a new regulatory test. Vaccine 19:1477-1483. 2001.

Shimoji Y, Yokomizo Y, Mori Y. Intracellular survival and replication of *Erysipelothrix rhusiopathiae* within murine macrophages: failure of induction of the oxidative burst of macrophages. Infection and Immunity 64:1789-1793. 1996.

To N and Nagai S. Genetic and antigenic diversity of the surface protective antigen proteins of *Erysipelothrix rhusiopathiae*. Clinical and Vaccine Immunology 14:813-820. 2007.

For your further reference, I have included information regarding the references cited in my letter.

Sincerely,

Richard E. Hill, Jr.
Director

Enclosures

USDA

August 31, 2009

United States
Department of
Agriculture

Animal and Plant
Health Inspection
Service

Veterinary Services

Center for Veterinary
Biologics

1920 Dayton Avenue
PO Box 844
Ames, IA 50010
(515) 337-6100

CENTER FOR VETERINARY BIOLOGICS NOTICE NO. 09-20

TO: Biologics Licensees, Permittees, and Applicants
Directors, Center for Veterinary Biologics
Veterinary Services Management Team

FROM: Richard E. Hill, Jr. /s/ Richard E. Hill, Jr.
Director
Center for Veterinary Biologics

SUBJECT: Withdrawal of Supplemental Assay Methods 601, 605, and 606

I. PURPOSE

The purpose of this document is to notify veterinary biologics manufacturers that Supplemental Assay Method for Potency Testing of Erysipelas Antiserum in Mice (SAM 601), Supplemental Assay Method for the Potency Testing of Erysipelas Bacterins in Swine (SAM 605), and Supplemental Assay Method for the Potency Testing of Erysipelas Vaccines in Swine (SAM 606) will be removed from the list of approved SAMs, effective immediately.

II. BACKGROUND

SAM 601 describes an assay method using mice, in which the degree of passive protection against a known challenge of *Erysipelas rhusiopathiae* is determined. SAM 605 describes an *in vivo* test for determining the potency of an Erysipelas bacterin in swine. SAM 606 describes an *in vivo* method for determining the potency of an Erysipelas vaccine, live culture (avirulent or modified) by challenging vaccinated swine with a virulent culture of *Erysipelothrix rhusiopathiae*.

III. ACTION

Biologic manufacturers who utilized SAMs 601, 605, 606 as assay method(s) in studies currently in progress may complete those studies as planned. Protocols and reports of future studies should not reference these documents. Biologics manufacturers who utilize these SAMs as procedures in current Outlines of Production should update these Outlines of Production by deleting references to these SAMs and describing the procedures either in the Outline of Production or Special Outline within 1 year of the date of this notice.

APHIS Safeguarding American Agriculture
APHIS is an agency of USDA's Marketing and Regulatory Programs
An Equal Opportunity Provider and Employer

Federal Relay Service
(Voice/TTY/ASCII/Spanish)
1-800-877-8339

Center for Veterinary Biologics
and
National Veterinary Services Laboratories
Testing Protocol

Supplemental Assay Method for Potency Testing of Erysipelas Antiserum in Mice

Date: March 19, 1999

Supersedes: May 26, 1982

Number: BBSAM0601.01

Standard Requirement: 9 CFR, Part 113.452

Contact Person: Charles Egemo, (515) 663-7407

Approvals:

/s/ Linda K. Schlater Date: 19 Mar 99
Linda R. K. Schlater, Head
Biologics Bacteriology Section

/s/ P Frank Ross Date: 19 Mar 99
P. Frank Ross, Acting Quality Assurance Manager

/s/ Randall L. Levings Date: 3/19/99
Randall L. Levings, Director
Center for Veterinary Biologics-Laboratory

United States Department of Agriculture
Animal and Plant Health Inspection Service
P. O. Box 844
Ames, IA 50010

Mention of trademark or proprietary product does not constitute a guarantee or warranty of the product by USDA and does not imply its approval to the exclusion of other products that may be suitable.

UNCONTROLLED COPY

Supplemental Assay Method for Potency Testing Erysipelas Antiserum in Mice

Table of Contents

1. Introduction

2. Materials

   2.1 Equipment/instrumentation
   2.2 Reagents/supplies
   2.3 Animals

3. Preparation for the test

   3.1 Personnel qualifications/training
   3.2 Selection and handling of test mice
   3.3 Preparation of supplies/equipment
   3.4 Preparation of reagents

4. Performance of test

   4.1 Injection of test animals with antiserum
   4.2 Preparation of challenge
   4.3 Timing and administration of challenge
   4.4 Postinoculation plate count
   4.5 Observation of mice after challenge

5. Interpretation of test results

6. Report of test results

7. References

8. Summary of revisions

UNCONTROLLED COPY

Supplemental Assay Method for Potency Testing Erysipelas Antiserum in Mice

1. Introduction

This Supplemental Assay Method (SAM) describes procedures for determining potency *Erysipelothrix rhusiopathiae* antiserum, as prescribed in the Code of Federal Regulations, Title 9 (9 CFR), Part 113.452. It is a test designed to use mice, in which the degree of passive protection against a known challenge of *E. rhusiopathiae* is determined.

2. Materials

2.1 Equipment/instrumentation

2.1.1 Spectrophotometer, Spectronic 70™ (Bausch and Lomb, Rochester, New York) or equivalent

2.1.2 Incubator, 37°C

2.1.3 Automatic pipetting device, or pipette bulb

2.1.4 Crimper for aluminum caps on serum vials

2.2 Reagents/supplies

2.2.1 *E. rhusiopathiae* strain E1-6 challenge culture, IRP ERC, current lot. This culture is available from the United States Department of Agriculture (USDA), Animal and Plant Health Inspection Service (APHIS), Center for Veterinary Biologics-Labortory, Ames, IA.

2.2.2 Positive control *E. rhusiopathiae* antiserum, IRP ERHU AS, current lot. This control serum is available from the CVB-L.

2.2.3 Test antiserum

2.2.4 Syringes, 1 ml

2.2.5 Needles, 26 ga x δ in

2.2.6 Glass serum bottle, 20-100 ml

2.2.7 Rubber stopper, 13 x 20 mm, and aluminum cap for serum bottle

2.2.8 Glass screw-top tubes, 13 x 100 mm, with caps

2.2.9 Pipettes, 5 ml and 25 ml

2.2.10 Erysipelas challenge culture medium

2.2.11 Bovine blood agar plates

2.2.12 Peptone buffer

2.2.13 Water, distilled or deionized, or water of equivalent purity

2.3 Animals

2.3.1 Swiss mice, 16-20 g. Although the 9 CFR does not specify sex, the CVB-L uses female mice.

2.3.2 Forty mice are required for each lot of antiserum to be tested. Forty additional mice are required for the positive control antiserum, and 10 mice are required as negative controls. Thirty mice are required to determine the LD_{50} of the challenge inoculum. All mice should be from the same source colony. **Note:** Although 9 CFR regulations do not require the use of a positive control antiserum or a determination of LD_{50}, the CVB-L uses these additional mice as checks on test validity.

3. Preparation for the test

3.1 Personnel qualifications/training

Technical personnel must have working knowledge of the use of general laboratory chemicals, equipment, and glassware and have specific training and experience in sterile technique, the handling of live bacterial cultures, and the handling of mice.

3.2 Selection and handling of test mice

3.2.1 Mice of either sex may be used, but females are recommended.

3.2.2 All mice must be housed and fed in a similar manner.

3.2.3 Identify each cage of mice by treatment group.

3.2.4 If any mice die after vaccination, but prior to challenge with live *E. rhusiopathiae*, necropsy these mice to determine cause of death if the cause of death is not outwardly apparent. If the cause of death is unrelated to vaccination, file the necropsy report with the test records, and no additional action is needed. If death is attributable to the test antiserum, report the death immediately to Inspection and Compliance, Center for Veterinary Biologics (CVB), which may request further safety testing of the antiserum.

3.2.5 When the test is concluded, instruct the animal caretakers to euthanize and incinerate the mice and to sanitize contaminated rooms.

3.3 Preparation of supplies/equipment

3.3.1 Sterilize all glassware before use.

3.3.2 Use only sterile supplies (pipettes, syringes, needles, rubber stoppers, diluents, etc.).

3.3.3 All equipment must be operated according to manufacturers' instructions and maintained and calibrated, as applicable, according to current CVB-L Standard Operating Procedures.

3.4 Preparation of reagents

3.4.1 *E. rhusiopathiae* challenge culture. The challenge culture, IRP ERC, is lyophilized in 0.2 ml amounts. Store vials of lyophilized culture at $\leq 4°C$.

3.4.2 Peptone buffer (NVSL media 10522)

Peptone	10 g
Sodium phosphate, dibasic	12.01 g
Potassium phosphate, monobasic	2.09g
Water	q.s. 1000ml

Adjust pH to 7.3-7.5. Autoclave 20 min at 121°C. Cool before using. Store at room temperature no more than 6 mo.

UNCONTROLLED COPY

3.4.3 Bovine blood agar (NVSL media 10006)

Blood agar base powder	40 g
Water	q.s. to 950 ml

Autoclave 20 min at 121°C. Cool to 47°C. Add 50 ml defibrinated bovine blood. Pour into sterile petri dishes. Cool to room temperature. Store at 4°C for no more than 6 mo.

3.4.4 Erysipelas challenge culture medium (NVSL media 10133)

Horse meat (no fat)	454 g
Horse liver	8 g
Water	1000 ml

Grind tissue and dispense in hot water in a cooker. Heat to boiling and simmer 1 hr. Allow to settle at least 2 hr. Skim off fat and discard meat. Strain through cheese cloth. Filter through No. 2 Whatman filter paper.
Combine 1000 ml of filtrate with:

Sodium phosphate, monobasic	11 g
Potassium phosphate, monobasic	1 g
Bile (fresh frozen fluid)	10 ml
**OR** Oxgall® (1 g in 10 ml H_2O)	
Peptone	20 g
Gelatin, granulated	5 g

Heat to just below boiling to dissolve the gelatin. Cool to 56°C. Adjust to pH 7.8.

Add:

Dextrose	5 g
Horse serum (not heat inactivated)	100 ml

Filter while still hot through a sterile Horman filter. Filter should also be hot. Adjust final pH to 7.6-7.8. Store at 4°C for no longer than 6 mo.

UNCONTROLLED COPY

4. Performance of the test

4.1 Injection of test animals with antiserum

4.1.1 Check the label on each product to confirm identity. Thoroughly mix product by inverting end-to-end.

4.1.2 Weigh 5 randomly selected mice immediately prior to injection to assure that the average body weight of the mice is between 16 and 20 g. Record weights.

4.1.3 Inject each of 40 mice subcutaneously with 0.1 ml of the test antiserum and each of 40 additional mice subcutaneously with 0.1 ml of the positive control antiserum.

4.1.4 Retain 10 noninjected mice as negative controls and 30 additional noninjected mice to determine LD_{50} of the challenge.

4.2 Preparation of challenge

4.2.1 Reconstitute a vial of IRP ERC challenge culture in 1.5 ml peptone buffer.

4.2.2 Inoculate 10 ml erysipelas challenge culture medium with entire contents of vial of reconstituted culture.

4.2.3 Incubate the inoculated broth at 37°C for 18-20 hr.

4.2.4 Perform a Gram stain, according to the current version of BBSOP0004, on the overnight cultures. If the Gram stain shows a pure culture of Gram positive rods, continue with the challenge procedure.

4.2.5 Dilute overnight culture, as necessary, in sterile erysipelas challenge culture medium to 70% ± 2% T at 600 nm, using a spectrophotometer.

Note: Use sterile erysipelas challenge culture medium as a blank for the spectrophotometer.

UNCONTROLLED COPY

4.2.6 Prepare a 10^{-5} dilution of the standardized culture in sterile erysipelas challenge medium. **This is the inoculum used to challenge the mice.** Place in a serum vial and seal with a rubber stopper and aluminum ring. Save an aliquot(s) of this inoculum in a separate vial(s); retain vial(s) as a sample for postchallenge plate counts.

4.2.7 Make 3 additional tenfold dilutions (10^{-2}, 10^{-3}, and 10^{-4}) of the challenge inoculum to determine LD_{50} of the challenge. Place each dilution in a separate labeled serum vial and seal.

4.2.8 Place all vials of challenge on ice to transport to animal room. Keep on ice throughout challenge procedure and until culture is added to plates for postinoculation plate count.

4.3 Timing and administration of challenge

4.3.1 Challenge all vaccinates 24 hr after injecting antiserum.

4.3.2 Challenge noninjected controls and LD_{50} mice at the same time as the injected mice.

4.3.3 Administer 0.2 ml of challenge inoculum subcutaneously to each mouse that received antiserum and to each of the negative control mice, using a 1-ml syringe with a 26-ga x δ-in needle.

4.3.4 Divide the LD_{50} mice into groups of 10. For each of the LD_{50} dilutions, inoculate each of 10 mice subcutaneously with 0.2 ml of the appropriate preparation.

4.4 Postinoculation plate count

4.4.1 After mice are challenged, perform a colony count on blood agar plates according to current version of BBSOP0019, using the vials retained for this purpose.

1. Use sterile erysipelas challenge culture broth as the diluent for the plate count, and plate on bovine blood agar. Incubate plates aerobically at 37°C for 48-72 hr.

UNCONTROLLED COPY

2. Calculate the colony-forming units (CFU) per challenge dose according to the following formula:

Note: Average count in 0.1 ml culture x 2 x dilution factor (see table below)=CFU/0.2 ml dose of challenge culture

If plates used for average count were inoculated with:	Dilution factor
10^{-2} dilution of challenge inoculum	100
10^{-3} dilution of challenge inoculum	1000
10^{-4} dilution of challenge inoculum	10000

4.5 Observation of mice after challenge

4.5.1 Observe the negative control mice and LD_{50} mice daily for 7 days after challenge. Observe the remaining mice (those receiving antiserum) for 10 days after challenge. Record deaths.

4.5.2 If deaths occurring after challenge are suspected to be due to causes other than erysipelas, necropsy such mice to determine the cause of death. If cause of death is unrelated to antiserum and/or challenge, do not include the deaths in the total deaths for the test.

5. Interpretation of test results

5.1 Interpret the test as prescribed in 9 CFR, Part 113.452.

5.1.1 At least 8 of 10 negative control mice must die within 7 days of challenge for a valid test. At least 34 of 40 mice receiving positive control antiserum must live for 10 days postchallenge for a valid test.

5.1.2 If at least 34 of 40 mice receiving the test antiserum survive 10 days after challenge, the serial is satisfactory without additional testing. If 11 or more mice die, the serial is unsatisfactory without additional testing. If 7-10 mice die, the serial is eligible for second stage testing.

5.1.3 Perform the second-stage test in a manner identical to the original test. Results of both tests

UNCONTROLLED COPY

are combined and interpreted according to the following chart:

Stage	Number of mice receiving antiserum	Cumulative number of mice receiving antiserum	Cumulative number of dead mice for....	
			Satisfactory serial	Unsatisfactory serial
1	40	40	6 or less	11 or more
2	40	80	12 or less	13 or more

5.1.4 Calculate the LD_{50} (theoretical dose/dilution at which the challenge would be lethal to 50% of the control mice) of the challenge inoculum, using the Reed-Muench method of estimation. Record on the test result form. This information is for informational purposes to track trends and to troubleshoot problem tests. The 9 CFR does not specify an acceptable LD_{50} range for this test.

5.1.5 Record the plate count (CFU/dose) of the challenge on the test result form. This information is for informational purposes to track trends and to troubleshoot problem tests. The 9 CFR does not specify a minimum or maximum LD_{50} or CFU/dose for this test.

6. Report of test results

Report the results of the test(s) as described by the current version of BBSOP0020.

7. References

7.1 Code of Federal Regulations, Title 9, Part 113.452, U.S. Government Printing Office, Washington, DC, 1998.

7.2 Reed LJ, Muench H, 1938. A simple method of estimating fifty percent endpoints. *Am J Hygiene*, 27:493-497.

UNCONTROLLED COPY

8. Summary of revisions

This document was rewritten to meet the current NVSL/CVB QA requirements, to clarify practices currently in use in the CVB-L, and to provide additional detail. No significant changes were made from the previous protocol.

UNCONTROLLED COPY

UNTED STATES DEPARTMENT OF AGRICULTURE
ANIMAL AND PLANT HEALTH INSPECTION SERVICE
VETERINARY SERVICES
Biologics Bacteriology Laboratory
P. O. Box 844
Ames, Iowa 50010

SAM 605

9 CFR 113.104(e) | May 25, 1982 Supersedes November 1, 1974 | *Erysipelothrix rhusiopathiae* Agent

SUPPLEMENTAL ASSAY METHOD FOR POTENCY TESTING OF ERYSIPELAS BACTERINS IN SWINE

A. SUMMARY

This is a method for determining the potency of an Erysipelas bacterin, as set forth in 9 CFR 113.104(e). It is a test to determine the immunity of vaccinated swine by challenging with a virulent culture of *Erysipelothrix rhusiopathiae*.

B. MATERIALS

1. Animals - pigs, susceptible to erysipelas, 20-30 pounds.

2. Media and Diluent

a. One percent Peptone in Soil Buffer solution

Peptone	10.00 grams
Sodium Phosphate Dibasic (anhydrous)	12.02 grams
Potassium Phosphate Monobasic	2.09 grams
Distilled Water	1000.00 ml.

All ingredients are mixed thoroughly in a 2000 ml Erlenmeyer flask. The pH is adjusted to 7.5. Ninety-nine ml amounts are dispensed in milk dilution bottles with screw caps and sterilized by autoclaving for 15 minutes at 121°C.

b. Erysipelas Challenge Culture Media

The infusion is prepared as follows:

Horse Meat (no fat)	454 grams
Horse Liver	18 grams
Distilled Water	1000 ml

The meat and liver are thawed (if frozen) and fat is trimmed off. It is ground and dispersed in the hot distilled water in a stainless steel cooker with a spigot at the bottom. The infusion is heated to boiling, simmered (just below the boiling point) for one hour and then brought back to a boil for 3 to 5 minutes. The infusion is allowed to cool and settle

for at least two hours. The broth is drawn off through the spigot by gravity and filtered through No. 2 Whatman filter paper. The following ingredients are added per liter of filtered broth:

Sodium Phosphate Dibasic (anhydrous)	11 grams
Potassium Phosphate Monobasic	1 gram
Peptone	20 grams
Gelatin - granular	5 grams
Ox Bile (if frozen, thaw)	10 ml

The medium is adjusted to pH 8 with 10N NaOH before sterilization. The medium is sterilized by filtering through a Model 7B Hormann filter (filter grades D5 and D9). Ninety-nine ml of medium are dispensed into 160 ml milk dilution bottles. The final pH is adjusted to 7.6 to 7.8.

c. 5% Bovine Blood agar - plates.

d. Normal horse serum - sterile.

3. Test Materials

a. Challenge material - _Erysipelothrix_ _rhusiopathiae_ - Strain E 1-6.

b. Sample(s) of _Erysipelothrix_ _rhusiopathiae_ bacterin(s) to be tested.

4. Equipment (Sterile)

a. Serum bottle - 50 ml.

b. Spectrophotometer (e.g., Bausch and Lomb, Spec 70).

c. Syringes - 3 ml plastic disposable.

d. Needles - 20 gauge (1 inch).

e. Pro-Pipet.

f. Pipettes - 1 ml, 2 ml and 10 ml.

g. Test tubes - 13 x 75 screw cap (for spec) and 16 x 125 screw cap (for dilutions).

C. PROCEDURES

1. Upon receipt of samples a test series is assigned and worksheets are prepared with the following information: test series number, serial number, bacterin dose, route of vaccination, identity of challenge culture, dose of challenge culture, date of vaccination, challenge and termination, sex, color, and ear tag identification of swine, and the initials of the person conducting the test.

2. The test animals are received and observed for 1 week prior to initiation of test. The pigs are identified by ear tags and grouped by random selection. The temperature of each pig is taken for 3 days prior to vaccination to establish a normal range.

3. Four susceptible pigs are vaccinated with the bacterin to be tested according to the directions on the label with one recommended swine dose.

Four (4) susceptible pigs from the same source are randomly selected for controls. These animals are observed daily for abnormal reactions. Temperatures are taken three times a week prior to challenge to establish a normal range.

4. Preparation of the Challenge Inoculum

   a. Eighteen to twenty hours prior to time of challenge, each of two vials of Strain E 1-6 challenge culture is reconstituted with 1.5 ml of 1% peptone in soil buffer solution and mixed well. Ten (10) ml of sterile normal horse serum are added aseptically to each of three milk dilution bottles containing 90 ml of Erysipelas media (B.,2.,b.). Two of the three bottles are each inoculated with the entire volume of each seed vial. The third bottle is held as an uninoculated control. All three bottles are incubated at 37°C.

   b. An 18-20 hour culture is adjusted to 40% LT at 600 nm on a spectrophotometer. Tenfold dilutions are made of the 40% culture.

5. Fourteen to twenty-one days following vaccination, all test animals are challenged intramuscularly with 2 ml of the challenge dilution (e.g., 10^{-5}). Bacterial count of the challenge culture is done on 5% B.A. media.

D. OBSERVATION OF SWINE AFTER CHALLENGE

1. The swine are observed daily for clinical signs as described in 9 CFR 113.104(e) and temperatures taken for 7 days. This information is recorded on the worksheets.

E. INTERPRETATION

The results are interpreted in accordance with 9 CFR 113.104(e).

UNITED STATES DEPARTMENT OF AGRICULTURE
ANIMAL AND PLANT HEALTH INSPECTION SERVICE
VETERINARY SERVICES
Biologics Bacteriology Laboratory
P. O. Box 844
Ames, Iowa 50010

SAM 606

9 CFR 113.67

May 20, 1982
Supersedes
November 1, 1974

Erysipelothrix rhusiopathiae
Agent

SUPPLEMENTAL ASSAY METHOD

FOR

POTENCY TESTING OF

ERYSIPELAS VACCINES

IN SWINE

A. SUMMARY

This is a method for determining the potency of an Erysipelas vaccine, live culture (avirulent or modified) as set forth in 9 CFR 113.67. It is a test to determine the immunity of vaccinated swine by challenging with a virulent culture of *Erysipelothrix rhusiopathiae*.

B. MATERIALS

1. Animals - pigs susceptible to erysipelas, 20-50 pounds.

2. Media and Diluent

a. One percent peptone in soil buffer solution.

Peptone	10.00 grams
Sodium Phosphate Dibasic (Anhydrous)	12.02 grams
Potassium Phosphate Monobasic	2.09 grams
Distilled Water	1000.00 ml

All ingredients are mixed thoroughly in a 2000 ml Erlenmeyer flask. The pH is adjusted to 7.5. Ninety-nine ml amounts are dispensed in milk dilution bottles with screw caps and sterilized by autoclaving for 15 minutes at 121°C.

b. Erysipelas Challenge Culture Media

The infusion is prepared as follows:

Horse Meat (No Fat)	454 grams
Horse Liver	18 grams
Distilled Water	1000 ml

The meat and liver are thawed (if frozen) and fat is trimmed off. It is ground and dispersed in the hot distilled water in a stainless steel cooker with a spigot at the bottom. The infusion is heated to boiling, simmered (just below the boiling point) for 1 hour and then brought back to a boil for 3 to 5 minutes.

The infusion is allowed to cool and settle for at least two hours. The broth is drawn off through the spigot by gravity and filtered through No. 2 Whatman filter paper. The following ingredients are added per liter of filtered broth:

Sodium Phosphate Dibasic (Anhydrous)	11 grams
Potassium Phosphate Monobasic	1 gram
Peptone	20 grams
Gelatin - granular	5 grams
Ox Bile (if frozen, thaw)	10 ml

The medium is adjusted to pH 8 with 10N NaOH before sterilization. The medium is sterilized by filtering through a Model 7B Hormann Filter (Filter Grades D5 and D9). Ninety ml of medium are dispensed into 160 ml milk dilution bottles. The final pH is adjusted to 7.6 to 7.8.

c. 5% Bovine Blood Agar - plate.

d. Normal horse serum - sterile.

3. Test Materials

a. Challenge material - Erysipelas rhusiopathiae - Strain E 1-6.

b. Sample(s) of Erysipelothrix rhusiopathiae vaccine(s) to be tested.

4. Equipment (Sterile)

   a. Serum bottle - 50 ml.

   b. Spectrophotometer (Bausch & Lomb, Spec 70).

   c. Syringes - 3 ml plastic disposable.

   d. Needles - 20 gauge (1 inch).

   e. Pipettes - 1 ml, 2 ml and 10 ml.

   f. Pro-pipet.

   g. Test tubes - 13 x 75 screw cap (for spec) and 16 x 125 screw cap (for dilutions).

C. PROCEDURES

1. Upon receipt of samples, a test series is assigned, worksheets are prepared with the following information: test series number, serial number, vaccine dose, route of vaccination, identity of challenge culture, dose of challenge culture, date of vaccination, challenge, and termination, sex, color, and ear tag identification of swine, and the initials of the person conducting the test.

2. The test animals are received and observed for 1 week prior to initiation of test. The pigs are identified by ear tags and grouped by random selection. The temperature of each pig is taken for 3 days prior to vaccination to establish a normal range.

3. Four susceptible pigs are vaccinated with the vaccine to be tested according to the directions on the label with one

recommended swine dose.

Four (4) susceptible pigs from the same source are randomly selected for controls and housed separately from the vaccinates. These animals are observed daily for abnormal reactions. Temperatures are taken three times a week prior to challenge to establish a normal range.

4. Preparation of the Challenge Inoculum

   a. Eighteen to twenty hours prior to time of challenge, each of two vials of Strain E 1-6 challenge culture is reconstituted with 1.5 ml of 1% peptone in soil buffer solution and mixed well. Ten (10) ml of sterile normal horse serum is added aseptically to each of three milk dilution bottles containing ninety ml of Erysipelas media (B.2.b.). Two of the 3 bottles are each inoculated with the entire volume of each seed vial. The third bottle is held as an uninoculated control. All three bottles are incubated at 37°C.

   b. An 18-20 hour culture is adjusted to 40% LT at 600 nm on a spectrophotometer. Tenfold dilutions are made of the 40% culture.

5. Fourteen to twenty-one days following vaccination, all test animals are challenged intramuscularly with 2 ml of challenge dilution (e.g., 10^{-5}). Bacterial count of the challenge culture is done on 5% B.A. media.

D. OBSERVATION OF SWINE AFTER CHALLENGE

1. The swine are daily observed and temperatures taken for 7 days. This information is recorded on the worksheets.

E. INTERPRETATION

The results are interpreted in accordance with 9 CFR 113.67.
