# Supplementary material for: Bridging the Gap Between Validation and Implementation of Non-Animal Veterinary Vaccine Potency Testing Methods
Source: Animals (Basel). 2011 Nov 29;1(4):414–32. doi: 10.3390/ani1040414 (PMC4513470; doi:10.3390/ani1040414)
Supplement: Supplementary File 1 [file animals-01-00414-s001.zip › supplementary materials/25 CVB notice 09-20 erysipelas.pdf]

USDA

August 31, 2009

United States Department of Agriculture

Animal and Plant Health Inspection Service

Veterinary Services

Center for Veterinary Biologics

1920 Dayton Avenue
PO Box 844
Ames, IA 50010
(515) 337-6100

CENTER FOR VETERINARY BIOLOGICS NOTICE NO. 09-20

TO: Biologics Licensees, Permittees, and Applicants
Directors, Center for Veterinary Biologics
Veterinary Services Management Team

FROM: Richard E. Hill, Jr. /s/ Richard E. Hill, Jr.
Director
Center for Veterinary Biologics

SUBJECT: Withdrawal of Supplemental Assay Methods 601, 605, and 606

I. PURPOSE

The purpose of this document is to notify veterinary biologics manufacturers that Supplemental Assay Method for Potency Testing of Erysipelas Antiserum in Mice (SAM 601), Supplemental Assay Method for the Potency Testing of Erysipelas Bacterins in Swine (SAM 605), and Supplemental Assay Method for the Potency Testing of Erysipelas Vaccines in Swine (SAM 606) will be removed from the list of approved SAMs, effective immediately.

II. BACKGROUND

SAM 601 describes an assay method using mice, in which the degree of passive protection against a known challenge of *Erysipelas rhusiopathiae* is determined. SAM 605 describes an *in vivo* test for determining the potency of an Erysipelas bacterin in swine. SAM 606 describes an *in vivo* method for determining the potency of an Erysipelas vaccine, live culture (avirulent or modified) by challenging vaccinated swine with a virulent culture of *Erysipelothrix rhusiopathiae*.

III. ACTION

Biologic manufacturers who utilized SAMs 601, 605, 606 as assay method(s) in studies currently in progress may complete those studies as planned. Protocols and reports of future studies should not reference these documents. Biologics manufacturers who utilize these SAMs as procedures in current Outlines of Production should update these Outlines of Production by deleting references to these SAMs and describing the procedures either in the Outline of Production or Special Outline within 1 year of the date of this notice.
