# Supplementary material for: Bridging the Gap Between Validation and Implementation of Non-Animal Veterinary Vaccine Potency Testing Methods
Source: Animals (Basel). 2011 Nov 29;1(4):414–32. doi: 10.3390/ani1040414 (PMC4513470; doi:10.3390/ani1040414)
Supplement: Supplementary File 1 [file animals-01-00414-s001.zip › supplementary materials/27 USDA complaint results leptospirosis.pdf]

USDA

Animal Care

USDA, APHIS, Animal Care
ANIMAL WELFARE COMPLAINT

Complaint No.	Date Entered	Received By
WIL ~~FY#~~W-170	10-Aug-10	EMC

Referred To	Reply Due
Randy Ridenour, DVM, SACS & Tracy Thompson, VMO	

Facility or Person Complaint Filed Against

Name	Customer/License/Registration No.
Coloradom Serum Company*	84-R-0007/#1086

Address
P. O. Box 16428

City	State	Zip	Phone No
Denver	CO	80216	303-295-7527

Complainant

Name	Organization
Jeffrey Brown	PETA

Address
N/A

City	State	Zip	Phone No./Email address
N/A	N/A	N/A	310-437-8003 or JeffreyB@peta.org

How was complaint received?
email

Details of Complaint: Using guinea pigs for batch testing for Leptospira; causes pain and distress in the form of blood poisioning, kidney damage and anorexia.

Results: Inspection was perfomed on 8/26/10. Colorado Serum Company (CSC) performs codified tests for production of leptospira vaccine products for use in cattle and swine and must follow strict regulatory procedures according to the 9 CFR. In-vitro teting was approved for use in leptospira vaccine potency testing for dog vaccine in 2006 and for cattle and swine in 2009 but this requires an exemption from CVB which involves testing of the master seed of the various serovars of Leptospira that are used at the facility. CSC submitted 5 master seeds to CVB for testing in March 2009 and again in November 2009 but these did not pass the approval and CSC does not qualify for the exemption from live animal testing involving hamsters at this time. CSC intends to resubmit master seed samples in the near future (currently being cultured to ensure their potency and viability). Complaint allegations are determined to be invalid based on all the evidence provided during this inspection.

Application packet provided? Yes ☐
No ☒

Connie R Morris/CO/APHIS/USDA
08/10/2010 01:21 PM

To Evelyn M Celli/CO/APHIS/USDA@USDA
cc
bcc
Subject Fw: Complaint against the Colorado Serum Company by PETA (3)

Jeffrey Brown <JeffreyB@peta.org>
08/10/2010 07:25 AM

To <robert.m.gibbens@usda.gov>
cc
Subject Complaint against the Colorado Serum Company by PETA (3)

Please see the attached document.

Jeffrey Brown
Research Associate
Regulatory Testing Division
People for the Ethical Treatment of Animals
2898 Rowena Avenue, Suite 102
Los Angeles, California 90039
(310) 437-8003

2007_84-R-0007 records CSC.pdf

This report is required by law (7 USC 2143). Failure to report according to the regulations can result in an order to cease and desist and to be subject to penalties as provided for in Section 2150.

See reverse side for additional information.

Interagency Report Control No 0180-DOA-AN

UNITED STATES DEPARTMENT OF AGRICULTURE
ANIMAL AND PLANT HEALTH INSPECTION SERVICE

ANNUAL REPORT OF RESEARCH FACILITY

(TYPE OR PRINT)

1. REGISTRATION NO. 84-R-0007 CUSTOMER NO. 1086

FORM APPROVED
OMB NO. 0579-0036

2. HEADQUARTERS RESEARCH FACILITY *(Name and Address, as registered with USDA, include Zip Code)*

COLORADO SERUM COMPANY
P. O. BOX 16428
DENVER, CO 80216
(303) 295-7527

3. REPORTING FACILITY (List all locations where animals were housed or used in actual research, testing, teaching, or experimentation, or held for these purposes. Attach additional sheets if necessary.)

FACILITY LOCATIONS*(sites)*

See Attached Listing

REPORT OF ANIMALS USED BY OR UNDER CONTROL OF RESEARCH FACILITY *(Attach additional sheets if necessary or use APHIS FORM 7023A.)*

A. Animals Covered By The Animal Welfare Regulations	B. Number of animals being bred, conditioned, or held for use in teaching, testing, experiments, research, or surgery but not yet used for such purposes.	C. Number of animals upon which teaching, research, experiments, or tests were conducted involving no pain, distress, or use of pain-relieving drugs.	D. Number of animals upon which experiments, teaching, research, surgery, or tests were conducted involving accompanying pain or distress to the animals and for which appropriate anesthetic, analgesic, or tranquilizing drugs were used.	E. Number of animals upon which teaching, experiments, research, surgery or tests were conducted involving accompanying pain or distress to the animals and for which the use of appropriate anesthetic, analgesic, or tranquilizing drugs would have adversely affected the procedures, results, or interpretation of the teaching, research, experiments, surgery, or tests. *(An explanation of the procedures producing pain or distress in these animals and the reasons such drugs were not used must be attached to this report)*	F. TOTAL NO. OF ANIMALS (Cols. C + D + E)
4. Dogs	--	--	--	--	--
5. Cats	--	--	--	--	--
6. Guinea Pigs	82	277	238	1,975	2,490
7. Hamsters	65	644	0	491	1,135
8. Rabbits	1	0	115	0	115
9. Non-Human Primates	--	--	--	--	--
10. Sheep	--	--	--	--	--
11. Pigs	--	--	--	--	--
12. Other Farm Animals	--	--	--	--	--
13. Other Animals	--	--	--	--	--

ASSURANCE STATEMENTS

1) Professionally acceptable standards governing the care, treatment, and use of animals, including appropriate use of anesthetic, analgesic, and tranquilizing drugs, prior to, during, and following actual research, teaching, testing, surgery, or experimentation were followed by this research facility.

2) Each principal investigator has considered alternatives to painful procedures.

OCT 17 2007

3) This facility is adhering to the standards and regulations under the Act, and it has required that exceptions to the standards and regulations be specified and explained by the principal investigator and approved by the Institutional Animal Care and Use Committee (IACUC). **A summary of all the exceptions is attached to this annual report.** In addition to identifying the IACUC approved exceptions, this summary includes a brief explanation of the exceptions, as well as the species and number of animals affected.

4) The attending veterinarian for this research facility has appropriate authority to ensure the provision of adequate veterinary care and to oversee the adequacy of other aspects of animal care and use.

CERTIFICATION BY HEADQUARTERS RESEARCH FACILITY OFFICIAL
(Chief Executive Officer or Legally Responsible Institutional official)

I certify that the above is true, correct, and complete (7 U.S.C. Section 2143)

SIGNAT[illegible]AL OFFICIAL	NAME & TITLE OF C.E.O. OR INSTITUTIONAL OFFICIAL *(Type or Print)*	DATE SIGNED
(b)(6),(b)(7)(c)	(b)(6),(b)(7)(c)	10-12-07

APHIS [illegible] FORM 18-23 (Oct 88), which is obsolete
(AUG 91)

PART 1 - HEADQUARTERS

Colorado Serum Company

October 12, 2007

Dr. Robert M. Gibbens, DVM
Director, Western Region Animal Care
USDA-APHIS-Animal Care
2150 Centre Avenue
Building B Mail Stop #36011
Ft. Collins, CO 80526

Dear Dr. Gibbens:

Enclosed please find our Research Facility Annual Report (APHIS Form 7023).

Supplementary documents are included which were enclosed with the 2006 Annual Report.

1. "Investigation into Guinea Pig Analgesia.......... ", dated November 11, 2004.
2. "Investigation by Colorado Serum Company's IACUC into Hamster Analgesia...... ", dated July 22, 2004.

In each case we have found no new information which would allow a change in our approach to these issues.

We have also enclosed a description of "Symptoms in Control Guinea Pigs after Challenge with *Clostridium chauvoei*", dated December 19, 2004.

Throughout this year, provisions in 9CFR 117.4(e) were applied to all animals in tests conducted according to 9CFR 113.451(b), 9CFR 113.106(c), 9CFR 113.101(c), 9CFR 113.102 (c), 9CFR 113.103(c), 9CFR 113.104(c) and 9CFR 113.107(c).

In each test, guinea pigs or hamsters that showed clinical signs of illness due to the test were humanely destroyed if the illness progressed to the point when death was certain to occur without therapeutic intervention.

Please contact us if there are any questions.

Sincerely,

(b)(6),(b)(7)(c)

JNH:al
Enclosure

OCT 17 2007

OLORADO
rinary Products

(b)(2)High, (b)(7)(F) P.O. Box 16428 • Denver, Colorado 80216-0428
Phone: (303) 295-7527 • Fax: (303) 295-1923

Colorado Serum Company
License No. 84-R-0007

For FY 2006-2007 Annual Report of Registered Research Facility

Each animal in each referenced test was employed in testing a Licensed Veterinary Biological product as required by Federal Regulations as codified in Title 9CFR. Humane euthanasia of animals on 9CFR required tests is provided for in 9CFR 117.4(e) - Animals at Licensed Establishments - Test Animals (filed August 19, 1995). This is included as standard testing protocol. While limiting the duration of the pain and distress, it does not fulfill the description of use as described treatment in Section D of APHIS Form 7023, Page 1. Therefore, we feel obligated to continue to include these test animals in Section E.

Attending veterinarians and related employees have been informed and Colorado Serum Company is complying with this provision.

Explanation of usage of animals listed in Column E.

Line Item 6. Guinea Pigs

1975 guinea pigs were tested causing pain, for which no anesthetic, analgesics, or tranquilizers could be used. A detailed breakdown of guinea pigs by test reference is provided.

9CFR 113.451(b) Tetanus Antitoxin potency testing - 1547 guinea pigs

Humane endpoints were addressed for all animals on this test, thus reducing the duration of pain and suffering.

9CFR 113.106(c) Clostridium Chauvoei Bacterin potency testing - 377 guinea pigs

Humane endpoints were addressed for all animals on this test, thus reducing the duration of pain and suffering.

9CFR 113.107(c) Clostridium Haemolyticum Bacterin potency testing - 51 guinea pigs

Humane endpoints were addressed for all animals on this test, thus reducing the duration of pain and suffering.

OCT 17 2007

Colorado Serum Company
License No. 84-R-0007

Line Item 7 — Hamsters

491 hamsters were tested causing pain, for which no anesthetics, analgesics, or tranquilizers could be used. A detailed breakdown of hamsters by test referenced is provided.

9CFR 113.101(c) — Leptospira Pomona Bacterin potency testing - 119 hamsters

The 119 hamsters included required controls for potency tests [9CFR 113.101(c)(2)], plus necessary hamsters required to establish the validity of the challenge dose (10 to 10,000 hamster LD_{50}) by titration. 9CFR 113.101 (c)(3).

For all test hamsters, Colorado Serum Company specifically has applied provisions of 9CFR 117.4(e) wherein ill animals were humanely euthanized upon reaching a point where death was expected to occur.

9CFR 113.102(c) — Leptospira Icterohaemorrhagiae Bacterin potency testing - 119 hamsters

The 119 hamsters included required controls for potency tests [9CFR 113.102(c)(2)], plus necessary hamsters required to establish the validity of the challenge dose (10 to 10,000 hamster LD_{50}) by titration. 9CFR 113.102(c)(3).

For all test hamsters, Colorado Serum Company specifically has applied provisions of 9CFR 117.4(e) wherein ill animals were humanely euthanized upon reaching a point where death was expected to occur.

9CFR 113.103(c) — Leptospira Canicola Bacterin potency testing - 134 hamsters

The 134 hamsters included required controls for potency tests [9CFR 113.103(c)(2)], plus necessary hamsters required to establish the validity of the challenge dose (10 to 10,000 hamster LD_{50}) by titration. (CFR 113.103(c)(3).

OCT 17 2007

Colorado Serum Company
License No. 84-R-0007

Line Item 7	9CFR 113.103(c)	Continued
		For all test hamsters, Colorado Serum Company specifically has applied provisions of 9CFR 117.4(e) wherein ill animals were humanely euthanized upon reaching a point where death was expected to occur.
	9CFR 113.104(c)	Leptospira Grippotyphosa Bacterin potency testing - 119 hamsters
		The 119 hamsters included required controls for potency tests [(9CFR 113.104(c)(2)], plus necessary hamsters required to establish the validity of the challenge dose (10 to 10,000 hamster LD_{50}) by titration. 9CFR 113.104 (c)(3).
		For all test hamsters, Colorado Serum Company specifically has applied provisions of 9CFR 117.4(e) wherein ill animals were humanely euthanized upon reaching a point where death was expected to occur.

(b)(6),(b)(7)(c)

10-12-07
Date

OCT 17 2007

December [illegible], 2004

(b)(6),(b)(7)(c)

Subject: Symptoms in control guinea pigs after challenge with *Clostridium chauvoei* and *hemolyticum* during *Cl. chauvoei* and *hemolyticum* bacterin in vivo potency tests.

These are symptoms that I have personally observed during this potency test. I will also include some more detailed textbook description of these symptoms to use if you like.

This potency tests for both of these fractions involves a 3 day observation after IM challenge with ~100 LD_{50} of live bacteria (*Cl. chauvoei or hemolyticum*). Within 24 to 48 hours the first symptoms of disease begin to appear. Lethargy, anorexia, stiffness with reluctance to move, sero-purulent discharge from the festering injection site, and vocalization (associated with pain) when handled are frequent symptoms most commonly seen. The swelling at the injection site is hot and painful at first and eventually becomes cold and necrotic as the infection becomes systemic. A high fever is also associated with these Clostridial diseases. Once symptoms are obvious the disease progresses quickly and death can happen rapidly. Once obvious symptoms are observed, veterinary intervention and humane euthanasia is implemented whenever possible.

COLORADO
SERUM COMPANY

PHONE (303) 29[illegible]
FAX (303) 29[illegible]

(b)(2)High, (b)(7)(F) *P.O. Box 16428 Denver, Colorado 80216-0428*

November 11, 2004

Subject: Investigation by Colorado Serum Company's Institutional Animal Care and Use Committee into guinea pig analgesia and its possible usage in guinea pigs during the *Clostridium tetani* antitoxin potency tests done at Colorado Serum Company.

From:
Veteri (b)(6),(b)(7)(c)

To: Colorado Serum Company's Institutional Animal Care and Use Committee and Mr. (b)(6),(b)(7)(c) Colorado Serum Company.

Background: Use of guinea pigs for potency tests when developing Tetanus Antitoxin has been the requirement by USDA (9-CFR, 113.451) for many years. The potency test is essentially an in-vivo neutralization test that requires two guinea pigs each for controls and for each dilution. The two controls are injected subcutaneously with a 3 ml dose of the Standard Toxin-Antitoxin mixture. Injections shall be made in the same order that toxin is added to the dilutions of antitoxins. These shall be observed parallel with the titration of one or more unknown antitoxins. Two test guinea pigs will be used for each dilution of the unknown antitoxin (also a 3 ml dose, subcutaneously). Controls are observed until they are down and are unable to rise or stand under their own power. At this time they are humanely euthanized and the time of death is recorded in hours. For a satisfactory test, the controls must reach this point with clinical signs of tetanus within 24 hours of each other and within an overall time of 60 – 120 hours. The clinical signs to be observed are increased muscle tonus, curvature of the spine, asymmetry of the body outline when the resting animal is viewed from above, generalized spastic paralysis, particularly of the extensor muscles, inability to rise from a smooth surface when the animal is placed on its side, or any combination of these signs. If the control guinea pigs do not respond in this manner the entire test shall be repeated. Potency of an unknown antitoxin is determined by finding the mixture which will protect the test animal the same as the Standard Toxin-Antitoxin mixture. Test animals dying sooner than the controls indicate the unit value selected in that dilution was not present, whereas those living longer indicate a greater unit value.

Analgesia in Guinea Pigs: An on-line and textbook search conducted by Colorado Serum Company's IACUC (b)(6),(b)(7)(c) revealed a variety of drugs used in guinea pigs after surgical procedures. The Duke University IACUC website had a very thorough

OCT 17 2007

U. S. VETERINARY LICENSE NO. 188

guideline for analgesia in all rodents and a variety of drugs and routes of administration based on the degree of pain and discomfort of a given procedure (see attached). Injectable analgesic options for guinea pigs include Codeine, Nalbuphine or Morphine given every 4 hours or Buprenorphine every 12 hours beginning just before symptoms are estimated to begin. Oral medications include Aspirin or Phenylbutazone added to the drinking water once daily, however once guinea pigs begin to show signs of tetanus it can be expected that their water consumption will diminish greatly and that once they are prostrate they will physically be unable to stand and drink. At the later stages of tetanus they are unable to swallow. Euthanasia hopefully is done before the late stages of disease but unfortunately death is sometimes the outcome with the test pigs before the study investigator can officially call the test over, if they have a lower level of antibody protection when compared to the Standard Controls.

Conclusion: There is obviously a direct conflict between animal welfare issues (pain and suffering) and this neutralization potency test that is required by USDA-APHIS-CVB for each new serial of tetanus antitoxin. The dilemma is that the test relies solely on symptoms of tetanus in relation to the Standard Controls as described above, in essence pain and suffering are components of the measured parameters of the test and giving any drugs to alter or help alleviate the symptoms will affect the results of the test. Currently the best welfare that can be provided is humane euthanasia once the symptoms have reached the point that the study investigator can interpret the test and give the staff veterinarian the o.k. to intervene and euthanize.

Due to this direct conflict, Colorado Serum Company's Institutional Animal Care and Use Committee has determined that there is no practical way to intervene with pain medications during the Tetanus Antitoxin potency neutralization test without altering the animal symptoms and thus altering the interpretation of the test. The only solution to this is to replace the guinea pig test with an in-vitro test (which Colorado Serum Company would be greatly in favor of). Currently there is a Competitive Elisa Test for Tetanus Antitoxin that has been tried but unfortunately it has not shown consistent, comparable results when run in parallel with the guinea pig neutralization test.

(b)(6),(b)(7)(c)

OCT 1 7 2007

COLORADO SERUM COMPANY

PHONE: [illegible]
FAX: [illegible]
(b)(2)High, (b)(7)(F) P.O. Box 16428 Denver, Colorado 80216-0428

July 22, 2004

Subject: Investigation by Colorado Serum Company's Institutional Animal Care and Use Committee into hamster analgesia and its possible usage in hamsters during the *Leptospira* challenge portion of *Leptospira* potency tests at Colorado Serum Company

From: (b)(6),(b)(7)(c)

To: Colorado Serum Company Institutional Animal Care and Use Committee and Mr. (b)(6),(b)(7)(c) Colorado Serum Company

Background: Use of hamsters for potency tests when developing Leptospira organisms for bacterin production has been the requirement by USDA (9-CFR, 113.101 – 113.105) for many years. Ten vaccinates and 10 or more controls are challenged intraperitoneally with a suspension of virulent Leptospira organisms. This applies to potency tests for *Leptospira pomona*, *Leptospira icterohaemorrhagiae*, *Leptospira canicola* and *Leptospira grippotyphosa* fractions. If 8 or more controls die from leptospirosis during a 14 day post challenge observation period then the test is considered valid. The degree of pain and suffering to the control hamsters is of concern for animal welfare reasons and an alternative in-vitro test that has reproducible and comparable results to the in-vivo potency test would be a much better alternative replacement test. Until that happens all biologic companies producing *Leptospira* bacterins will continue to use hamster in-vivo potency tests.

The degree of pain and suffering the control hamsters endure during this 14 day observation period is a subjective evaluation given the fact that hamsters cannot communicate with humans. Based on the disease syndrome of Leptospirosis in our domestic species the clinical signs can be mild to severe. In most animal species the disease causes septicemia, nephritis, depression and anorexia. It is safe to assume that hamsters suffer from many of these symptoms as well, and it is most likely not a chronic pain as much as it is a general discomfort or sick feeling that they are experiencing. Parameters of hamster behavior used to measure discomfort or pain would include exploration, grooming and posture as well as food and water consumption and fur quality.

Analgesic use in hamsters: An on-line and textbook search conducted by Colorado Serum Company's IACUC (b)(6),(b)(7)(c) revealed very few drug options for analgesia in hamsters (see attached documents). Buprenorphine was the most common analgesic for hamsters cited. The labeled route is by subcutaneous injection and the

frequency is every 8 hours. In one published paper (see attached) buprenorphine was used in the syngeneic murine tumor model to investigate whether the pain or discomfort in mice with these tumors could be reduced with this analgesic via an oral route. In this study they found no significant difference in pain or discomfort between the buprenorphine treated group and the control group based on their scoring system. Even though this experiment was done with mice it would be plausible to compare the discomfort or pain from multiple tumors to that which would be experienced by hamsters during a systemic leptospirosis infection. Also the process of handling these hamsters and giving them injections 2 – 3 times daily would itself be stressful and painful. There would be a significant zoonotic concern for the person involved giving the injections to multiple infected hamsters from the urine as well as an increased chance of being bitten considering you are giving injections to sick and uncomfortable hamsters 2 to 3 times daily for up to 14 days. Treating the symptoms without treating the source of the disease with antibiotics (which can't be done for obvious reasons) is also of questionable value. There would also be concern as to what effect this analgesic would have on the outcome of the potency test. All drugs are processed and eliminated by the liver and kidneys and since these are two target organs of *Leptospira* organisms, toxic levels of buprenorphine could buildup in a *Leptospira* infected hamster and could alter the time of death or even contribute to a death that might not otherwise happen - thus affecting the outcome of the potency test and possibly resulting in a falsely valid test. Other drugs mentioned are injectable also and do not offer anything unique or superior to buprenorphine.

The Cornell website detailed an oral preparation of buprenorphine in jello for use in mice (see attached). Whether this would work in hamsters is not known. One concern is the fact that anorexia is a very common symptom with Leptospirosis and depending on the degree of anorexia and the fact that these hamsters are usually housed 5 per cage, some hamsters may be getting their medication and some may not - due to anorexia or competition. This could also cause some hamsters to overdose on buprenorphine if they all aren't eating the same amount of jello.

Conclusion:
Colorado Serum Company's IACUC has determined that no analgesia we have found can realistically be expected to relieve an infected hamster from the symptoms of Leptospirosis especially without concurrent use of antibiotics to fight the etiologic agent (*Leptospira*) since this would defeat the purpose of the potency study. Buprenorphine works well with acute, sharp post-surgical type pain and would be of very questionable value in hamsters suffering from discomfort associated with a systemic infection. The best course of action in our opinion would be to replace the hamster in-vivo potency test with an in-vitro test that does not require animal testing for potency.

(b)(6),(b)(7)(c)

Connie R Morris/CO/APHIS/USDA
08/10/2010 01:22 PM

To Evelyn M Celli/CO/APHIS/USDA@USDA
cc
bcc
Subject Fw: Complaint against the Colorado Serum Company by PETA (4)

Jeffrey Brown <JeffreyB@peta.org>
08/10/2010 07:25 AM

To <robert.m.gibbens@usda.gov>
cc
Subject Complaint against the Colorado Serum Company by PETA (4)

Please see the attached document.

Jeffrey Brown
Research Associate
Regulatory Testing Division
People for the Ethical Treatment of Animals
2898 Rowena Avenue, Suite 102
Los Angeles, California 90039
(310) 437-8003

2008_84-R-0007 records CSC.pdf

This report is required by law (7 USC 2143). Failure to report according to the regulations can result in an order to cease and desist and to be subject to penalties as provided for in Section 21!

See attached form for additional information.

Interagency Report Control No.:

UNITED STATES DEPARTMENT OF AGRICULTURE
ANIMAL AND PLANT HEALTH INSPECTION SERVICE

ANNUAL REPORT OF RESEARCH FACILITY

(TYPE OR PRINT)

1. CERTIFICATE NUMBER: 84-R-0007
CUSTOMER NUMBER: 1086

FORM APPROVED
OMB NO. 0579-0036

Colorado Serum Company
P. O. Box 16428
Denver, CO 80216

Telephone: (303) -295-7527

3. REPORTING FACILITY (List all locations where animals were housed or used in actual research, testing, or experimentation, or held for these purposes. Attach additional sheets if necessary)

FACILITY LOCATIONS (Sites) - See Atached Listing

REPORT OF ANIMALS USED BY OR UNDER CONTROL OF RESEARCH FACILITY (Attach additional sheets if necessary or use APHIS Form 7023A)

A. Animals Covered By The Animal Welfare Regulations	B. Number of animal being bred, conditioned, or held for use in teaching, testing, experiments, research, or surgery but not ye used for such purposes.	C. Number of animals upon which teaching, research, experiments, or tests were conducted involving no pain, distress, or use o pain-relieving drugs.	D. Number of animals upon which experiments, teaching, research, surgery, or tests were conducted involving accompanying pain or distress to the animals an for which appropriate anesthetic, analgesic, or tranquilizing drugs were used.	E. Number of animals upon which teaching, experiments, research, surgery or tests were conducted involving accompanying pain or distress to the animals and for wh the use of appropriate anesthetic, analgesic, or tranquiliz drugs would have adversely affected the procedures, res or interpretation of the teaching, research, experiments, surgery, or tests. (An explanation of the procedures producing pain or distress in these animals and the reas such drugs were not used must be attached to this report	F. TOTAL NUMBER OF ANIMALS (COLUMNS C + D + E)
4. Dogs	--	--	--	--	--
5. Cats	--	--	--	--	--
6. Guinea Pigs	82	487	373	1,173	2,033
7. Hamsters	103	857	0	533	1,390
[illegible] Rabbits	0	0	177	0	177
[illegible] Non-human Primates	--	--	--	--	--
10. Sheep	--	--	--	--	--
11. Pigs	--	--	--	--	--
12. Other Farm Animals	--	--	--	--	--
13. Other Animals	--	--	--	--	--

ASSURANCE STATEMENTS

1) Professionally acceptable standards governing the care, treatment, and use of animals, including appropriate use of anestetic, analgesic, and tranquilizing drugs, prior to, during, and following actual rese teaching, testing, surgery, or experimentation were followed by this research facility.

2) Each principal investigator has considered alternatives to painful procedures.

3) This facility is adhering to the standards and regulations under the Act, and it has required that exceptions to the standards and regulations be specified and explained by the principal investigator and ap Institutional Animal Care and Use Committee (IACUC). **A summary of all such exceptions is attached to this annual report.** In addition to identifying the IACUC-approved exceptions, this summary inc brief explanation of the exceptions, as well as the species and number of animals affected.

4) The attending veterinarian for this research facility has appropriate authority to ensure the provision of adequate veterinary care and to oversee the adequacy of other aspects of animal care and use.

CERTIFICATION BY HEADQUARTERS RESEARCH FACILITY OFFICIAL
(Chief Executive Officer or Legally Responsible Institutional Official)

SIGNAT[illegible]	NAM[illegible] IAL *(Type or Print)*	DATE SIGNED
(b)(6),(b)(7)(c)	(b)(6),(b)(7)(c)	10-16-08

IIS FC[illegible] (OCT 88), which is obsolete.)
(AU[illegible]

OCT 17 2008

2010-11-16 - 000017

Colorado Serum Company
License No. 84-R-0007

For FY 2007-2008 Annual Report of Registered Research Facility:

Each animal in each referenced test was employed in testing a Licensed Veterinary Biological product as required by Federal Regulations as codified in Title 9CFR. Humane euthanasia of animals on 9CFR required tests is provided for in 9CFR 117.4(e) - Animals at Licensed Establishments - Test Animals (filed August 19, 1995). This is included as standard testing protocol. While limiting the duration of the pain and distress, it does not fulfill the description of use as described treatment in Section D of APHIS Form 7023, Page 1. Therefore, we feel obligated to continue to include these test animals in Section E.

Attending veterinarians and related employees have been informed and Colorado Serum Company is complying with this provision.

Explanation of usage of animals listed in Column E.

Line Item 6. Guinea Pigs

1173 guinea pigs were tested causing pain, for which no anesthetic, analgesics, or tranquilizers could be used. A detailed breakdown of guinea pigs by test reference is provided.

9CFR 113.451(b) Tetanus Antitoxin potency testing - 1019 guinea pigs

Humane endpoints were addressed for all animals on this test, thus reducing the duration of pain and suffering.

9CFR 113.106(c) Clostridium Chauvoei Bacterin potency testing - 112 guinea pigs

Humane endpoints were addressed for all animals on this test, thus reducing the duration of pain and suffering.

9CFR 113.107(c) Clostridium Haemolyticum Bacterin potency testing - 42 guinea pigs

Humane endpoints were addressed for all animals on this test, thus reducing the duration of pain and suffering.

OCT 17 2008

Colorado Serum Company
License No. 84-R-0007

Line Item 7 Hamsters

533 hamsters were tested causing pain, for which no anesthetics, analgesics, or tranquilizers could be used. A detailed breakdown of hamsters by test referenced is provided.

9CFR 113.101(c) Leptospira Pomona Bacterin potency testing - 200 hamsters

The 200 hamsters included required controls for potency tests [9CFR 113.101(c)(2)], plus necessary hamsters required to establish the validity of the challenge dose (10 to 10,000 hamster LD_{50}) by titration. 9CFR 113.101 (c)(3).

For all test hamsters, Colorado Serum Company specifically has applied provisions of 9CFR 117.4(e) wherein ill animals were humanely euthanized upon reaching a point where death was expected to occur.

9CFR 113.102(c) Leptospira Icterohaemorrhagiae Bacterin potency testing - 100 hamsters

The 100 hamsters included required controls for potency tests [9CFR 113.102(c)(2)], plus necessary hamsters required to establish the validity of the challenge dose (10 to 10,000 hamster LD_{50}) by titration. 9CFR 113.102(c)(3).

For all test hamsters, Colorado Serum Company specifically has applied provisions of 9CFR 117.4(e) wherein ill animals were humanely euthanized upon reaching a point where death was expected to occur.

9CFR 113.103(c) Leptospira Canicola Bacterin potency testing - 82 hamsters

The 82 hamsters included required controls for potency tests [9CFR 113.103(c)(2)], plus necessary hamsters required to establish the validity of the challenge dose (10 to 10,000 hamster LD_{50}) by titration. (CFR 113.103(c)(3).

OCT 17 2008

Colorado Serum Company
License No. 84-R-0007

Line Item 7	9CFR 113.103(c)	Continued<br><br>For all test hamsters, Colorado Serum Company specifically has applied provisions of 9CFR 117.4(e) wherein ill animals were humanely euthanized upon reaching a point where death was expected to occur.
	9CFR 113.104(c)	Leptospira Grippotyphosa Bacterin potency testing - 151 hamsters<br><br>The 151 hamsters included required controls for potency tests [(9CFR 113.104(c)(2)], plus necessary hamsters required to establish the validity of the challenge dose (10 to 10,000 hamster LD_{50}) by titration. 9CFR 113.104 (c)(3).<br><br>For all test hamsters, Colorado Serum Company specifically has applied provisions of 9CFR 117.4(e) wherein ill animals were humanely euthanized upon reaching a point where death was expected to occur.

(b)(6),(b)(7)(c) ________________

10-16-08
Date

OCT 17 2008

84R 0007
1086

COLORADO SERUM COMPANY

PHONE: (303) 295-7527
FAX: (303) 295-1923
(b)(2)High, (b)(7)(F) P.O. Box 16428 Denver, Colorado 80216-0428

November 11, 2004

Subject: Investigation by Colorado Serum Company's Institutional Animal Care and Use Committee into guinea pig analgesia and its possible usage in guinea pigs during the *Clostridium tetani* antitoxin potency tests done at Colorado Serum Company.

From:
Veteri (b)(6),(b)(7)(c)

To: Colorado Serum Company's Institutional Animal Care and Use Committee and Mr. (b)(6),(b)(7)(c) Colorado Serum Company.

Background: Use of guinea pigs for potency tests when developing Tetanus Antitoxin has been the requirement by USDA (9-CFR, 113.451) for many years. The potency test is essentially an in-vivo neutralization test that requires two guinea pigs each for controls and for each dilution. The two controls are injected subcutaneously with a 3 ml dose of the Standard Toxin-Antitoxin mixture. Injections shall be made in the same order that toxin is added to the dilutions of antitoxins. These shall be observed parallel with the titration of one or more unknown antitoxins. Two test guinea pigs will be used for each dilution of the unknown antitoxin (also a 3 ml dose, subcutaneously). Controls are observed until they are down and are unable to rise or stand under their own power. At this time they are humanely euthanized and the time of death is recorded in hours. For a satisfactory test, the controls must reach this point with clinical signs of tetanus within 24 hours of each other and within an overall time of 60 – 120 hours. The clinical signs to be observed are increased muscle tonus, curvature of the spine, asymmetry of the body outline when the resting animal is viewed from above, generalized spastic paralysis, particularly of the extensor muscles, inability to rise from a smooth surface when the animal is placed on its side, or any combination of these signs. If the control guinea pigs do not respond in this manner the entire test shall be repeated. Potency of an unknown antitoxin is determined by finding the mixture which will protect the test animal the same as the Standard Toxin-Antitoxin mixture. Test animals dying sooner than the controls indicate the unit value selected in that dilution was not present, whereas those living longer indicate a greater unit value.

Analgesia in Guinea Pigs: An on-line and textbook search conducted by Colorado Serum Company's IACUC (b)(6),(b)(7)(c) revealed a variety of drugs used in guinea pigs after surgical procedures. The Duke University IACUC website had a very thorough

OCT 17 2008

guideline for analgesia in all rodents and a variety of drugs and routes of administration based on the degree of pain and discomfort of a given procedure (see attached). Injectable analgesic options for guinea pigs include Codeine, Nalbuphine or Morphine given every 4 hours or Buprenorphine every 12 hours beginning just before symptoms are estimated to begin. Oral medications include Aspirin or Phenylbutazone added to the drinking water once daily, however once guinea pigs begin to show signs of tetanus it can be expected that their water consumption will diminish greatly and that once they are prostrate they will physically be unable to stand and drink. At the later stages of tetanus they are unable to swallow. Euthanasia hopefully is done before the late stages of disease but unfortunately death is sometimes the outcome with the test pigs before the study investigator can officially call the test over, if they have a lower level of antibody protection when compared to the Standard Controls.

Conclusion: There is obviously a direct conflict between animal welfare issues (pain and suffering) and this neutralization potency test that is required by USDA-APHIS-CVB for each new serial of tetanus antitoxin. The dilemma is that the test relies solely on symptoms of tetanus in relation to the Standard Controls as described above, in essence pain and suffering are components of the measured parameters of the test and giving any drugs to alter or help alleviate the symptoms will affect the results of the test. Currently the best welfare that can be provided is humane euthanasia once the symptoms have reached the point that the study investigator can interpret the test and give the staff veterinarian the o.k. to intervene and euthanize.

Due to this direct conflict, Colorado Serum Company's Institutional Animal Care and Use Committee has determined that there is no practical way to intervene with pain medications during the Tetanus Antitoxin potency neutralization test without altering the animal symptoms and thus altering the interpretation of the test. The only solution to this is to replace the guinea pig test with an in-vitro test (which Colorado Serum Company would be greatly in favor of). Currently there is a Competitive Elisa Test for Tetanus Antitoxin that has been tried but unfortunately it has not shown consistent, comparable results when run in parallel with the guinea pig neutralization test.

(b)(6),(b)(7)(c)

OCT 17 2008

/086

COLORADO SERUM COMPANY

PHONE: (303) 295-7527
FAX: (303) 295-1923
(b)(2)High, (b)(7)(F) P.O. Box 16428 Denver, Colorado 80216-0428

July 22, 2004

Subject: Investigation by Colorado Serum Company's Institutional Animal Care and Use Committee into hamster analgesia and its possible usage in hamsters during the *Leptospira* challenge portion of *Leptospira* potency tests at Colorado Serum Company.

From: (b)(6),(b)(7)(c)

To: Colorado Serum Company Institutional Animal Care and Use Committee and Mr. (b)(6),(b)(7)(c) Colorado Serum Company.

Background: Use of hamsters for potency tests when developing Leptospira organisms for bacterin production has been the requirement by USDA (9-CFR, 113.101 – 113.105) for many years. Ten vaccinates and 10 or more controls are challenged intraperitoneally with a suspension of virulent Leptospira organisms. This applies to potency tests for *Leptospira pomona, Leptospira icterohaemorrhagiae, Leptospira canicola* and *Leptospira grippotyphosa* fractions. If 8 or more controls die from leptospirosis during a 14 day post challenge observation period then the test is considered valid. The degree of pain and suffering to the control hamsters is of concern for animal welfare reasons and an alternative in-vitro test that has reproducible and comparable results to the in-vivo potency test would be a much better alternative replacement test. Until that happens all biologic companies producing *Leptospira* bacterins will continue to use hamster in-vivo potency tests.

The degree of pain and suffering the control hamsters endure during this 14 day observation period is a subjective evaluation given the fact that hamsters cannot communicate with humans. Based on the disease syndrome of Leptospirosis in our domestic species the clinical signs can be mild to severe. In most animal species the disease causes septicemia, nephritis, depression and anorexia. It is safe to assume that hamsters suffer from many of these symptoms as well, and it is most likely not a chronic pain as much as it is a general discomfort or sick feeling that they are experiencing. Parameters of hamster behavior used to measure discomfort or pain would include exploration, grooming and posture as well as food and water consumption and fur quality.

Analgesic use in hamsters: An on-line and textbook search conducted by Colorado Serum Company's IACUC (b)(6),(b)(7)(c) revealed very few drug options for analgesia in hamsters (see attached documents). Buprenorphine was the most common analgesic for hamsters cited. The labeled route is by subcutaneous injection and the

OCT 17 2008

THE PEAK OF QUALITY

U.S. VETERINARY LICENSE NO. 188

frequency is every 8 hours. In one published paper (see attached) buprenorphine was used in the syngeneic murine tumor model to investigate whether the pain or discomfort in mice with these tumors could be reduced with this analgesic via an oral route. In this study they found no significant difference in pain or discomfort between the buprenorphine treated group and the control group based on their scoring system. Even though this experiment was done with mice it would be plausible to compare the discomfort or pain from multiple tumors to that which would be experienced by hamsters during a systemic leptospirosis infection. Also the process of handling these hamsters and giving them injections 2 – 3 times daily would itself be stressful and painful. There would be a significant zoonotic concern for the person involved giving the injections to multiple infected hamsters from the urine as well as an increased chance of being bitten considering you are giving injections to sick and uncomfortable hamsters 2 to 3 times daily for up to 14 days. Treating the symptoms without treating the source of the disease with antibiotics (which can't be done for obvious reasons) is also of questionable value. There would also be concern as to what effect this analgesic would have on the outcome of the potency test. All drugs are processed and eliminated by the liver and kidneys and since these are two target organs of *Leptospira* organisms, toxic levels of buprenorphine could buildup in a *Leptospira* infected hamster and could alter the time of death or even contribute to a death that might not otherwise happen – thus affecting the outcome of the potency test and possibly resulting in a falsely valid test. Other drugs mentioned are injectable also and do not offer anything unique or superior to buprenorphine.

The Cornell website detailed an oral preparation of buprenorphine in jello for use in mice (see attached). Whether this would work in hamsters is not known. One concern is the fact that anorexia is a very common symptom with Leptospirosis and depending on the degree of anorexia and the fact that these hamsters are usually housed 5 per cage, some hamsters may be getting their medication and some may not - due to anorexia or competition. This could also cause some hamsters to overdose on buprenorphine if they all aren't eating the same amount of jello.

Conclusion:
Colorado Serum Company's IACUC has determined that no analgesia we have found can realistically be expected to relieve an infected hamster from the symptoms of Leptospirosis especially without concurrent use of antibiotics to fight the etiologic agent (*Leptospira*) since this would defeat the purpose of the potency study. Buprenorphine works well with acute, sharp post-surgical type pain and would be of very questionable value in hamsters suffering from discomfort associated with a systemic infection. The best course of action in our opinion would be to replace the hamster in-vivo potency test with an in-vitro test that does not require animal testing for potency.

(b)(6),(b)(7)(c)

OCT 17 2008

1086

December 29, 2004

(b)(6),(b)(7)(c)

Subject: Symptoms in control guinea pigs after challenge with *Clostridium chauvoei and hemolyticum* during *Cl. chauvoei* and *hemolyticum* bacterin in vivo potency tests.

These are symptoms that I have personally observed during this potency test. I will also include some more detailed textbook description of these symptoms to use if you like.

This potency tests for both of these fractions involves a 3 day observation after IM challenge with ~100 LD_{50} of live bacteria (*Cl. chauvoei or hemolyticum*). Within 24 to 48 hours the first symptoms of disease begin to appear. Lethargy, anorexia, stiffness with reluctance to move, sero-purulent discharge from the festering injection site, and vocalization (associated with pain) when handled are frequent symptoms most commonly seen. The swelling at the injection site is hot and painful at first and eventually becomes cold and necrotic as the infection becomes systemic. A high fever is also associated with these Clostridial diseases. Once symptoms are obvious the disease progresses quickly and death can happen rapidly. Once obvious symptoms are observed, veterinary intervention and humane euthanasia is implemented whenever possible.

OCT 17 [illegible]

Established 1923

Colorado Serum Company

October 16, 2008

Dr. Robert M. Gibbens, DVM
Director, Western Region Animal Care
USDA-APHIS-Animal Care
2150 Centre Avenue
Building B, Mail Stop #3W11
Ft. Collins, CO 80526

Dear Dr. Gibbens:

Enclosed please find our Research Facility Annual Report (APHIS Form 7023).

Supplementary documents are included which were enclosed with the 2007 Annual Report.

1. "Investigation into Guinea Pig Analgesia.......... ", dated November 11, 2004.
2. "Investigation by Colorado Serum Company's IACUC into Hamster Analgesia...... ", dated July 22, 2004.

In each case we have found no new information which would allow a change in our approach to these issues.

We have also enclosed a description of "Symptoms in Control Guinea Pigs after Challenge with *Clostridium chauvoei*", dated December 19, 2004.

Throughout this year, provisions in 9CFR 117.4(e) were applied to all animals in tests conducted according to 9CFR 113.451(b), 9CFR 113.106(c), 9CFR 113.101(c), 9CFR 113.102 (c), 9CFR 113.103(c), 9CFR 113.104(c) and 9CFR 113.107(c).

In each test, guinea pigs or hamsters that showed clinical signs of illness due to the test were humanely destroyed if the illness progressed to the point when death was certain to occur without therapeutic intervention.

Please contact us if there are any questions.

Sincerely,

(b)(6),(b)(7)(c)

JNH:al
Enclosure

COLORADO Veterinary Products

(b)(2)High, (b)(7)(F) P.O. Box 16428 • Denver, Colorado 80216-0428
Phone: (303) 295-7527• Fax: (303) 295-1923

OCT 17 2008

www.coloradoserum.com

U.S. Vet. License No. 188

Connie R Morris/CO/APHIS/USDA
08/10/2010 01:22 PM

To Evelyn M Celli/CO/APHIS/USDA@USDA
cc
bcc
Subject Fw: Complaint against the Colorado Serum Company by PETA (5)

Jeffrey Brown <JeffreyB@peta.org>
08/10/2010 07:26 AM

To <robert.m.gibbens@usda.gov>
cc
Subject Complaint against the Colorado Serum Company by PETA (5)

Please see the attached document.

Jeffrey Brown
Research Associate
Regulatory Testing Division
People for the Ethical Treatment of Animals
2898 Rowena Avenue, Suite 102
Los Angeles, California 90039
(310) 437-8003

2009_84-R-0007 records CSC.pdf

According to the Paperwork Reduction Act of 1995, an agency may not conduct or sponsor, and a person is not required to respond to, a collection of information unless it displays a valid OMB control number. The valid OMB control number for this information collection is 0579-0036. The time required to complete this information collection is estimated to average 2 hours per response, including the time for reviewing instructions, searching existing data sources, gathering and maintaining the data needed, and completing and reviewing the collection of information.

OMB APPROVED
0579-0036

This report is required by law (7 U.S.C. 2143). Failure to report according to the regulations can result in an order to cease and desist and to be subject to penalties as provided for in Section 2150.

Interagency Report Control No. 0180-DOA-AN

Fiscal Year: 2009

UNITED STATES DEPARTMENT OF AGRICULTURE
ANIMAL AND PLANT HEALTH INSPECTION SERVICE

ANNUAL REPORT OF RESEARCH FACILITY

(TYPE OR PRINT)

REGISTRATION NUMBER: 84-R-0007

Customer Number: 1086

2. HEADQUARTERS RESEARCH FACILITY *(Name and Address, as registered with USDA, include ZIP Code)*

Colorado Serum Company
P. O. Box 16428
Denver, CO 80216

[redacted]

3. REPORTING FACILITY *(List all locations where animals were housed or used in actual research, testing, teaching, or experimentation, or held for these purposes. Attach additional sheets if necessary.)*

FACILITY LOCATIONS *(Sites)* *See Attached Listing*

REPORT OF ANIMALS USED BY OR UNDER CONTROL OF RESEARCH FACILITY *(Attach additional sheets if necessary or use APHIS FORM 7023A.)*

A. Animals Covered By The Animal Welfare Regulations	B. Number of animals being bred, conditioned, or held for use in teaching, testing, experiments, research, or surgery but not yet used for such purposes	C. Number of animals upon which teaching, research, experiments, or tests were conducted involving no pain, distress, or use of pain-relieving drugs.	D. Number of animals upon which experiments, teaching, research, surgery, or tests were conducted involving accompanying pain or distress to the animals and for which appropriate anesthetic, analgesic, or tranquilizing drugs were used.	E. Number of animals upon which teaching, experiments, research, surgery, or tests were conducted involving accompanying pain or distress to the animals and for which the use of appropriate anesthetic, analgesic, or tranquilizing drugs would have adversely affected the procedures, results, or interpretation of the teaching, research, experiments, surgery, or tests. *(An explanation of the procedures producing pain or distress on these animals and the reasons such drugs were not used must be attached to this report.)*	F. TOTAL NUMBER OF ANIMALS (Cols. C + D + E)
4. Dogs	----	----	----	-----	-----
5. Cats	----	----	----	-----	-----
6. Guinea Pigs	51	319	228	1,238	1,785
7. Hamsters	13	434	0	388	822
8. Rabbits	11	0	200	0	200
9. Non-human Primates	——	——	——	——	——
10. Sheep	——	——	——	——	——
11. Pigs	——	——	——	——	——
12. Other Farm Animals	——	——	——	——	——
13. Other Animals	——	——	——	——	——

ASSURANCE STATEMENTS

1.) Professionally acceptable standards governing the care, treatment, and use of animals, including appropriate use of anesthetic, analgesic, and tranquilizing drugs, prior to, during, and following actual research, teaching, testing, surgery, or experimentation were followed by this research facility.

2.) Each principal investigator has considered alternatives to painful procedures.

3.) This facility is adhering to the standards and regulations under the Act, and it has required that exceptions to the standards and regulations be specified and explained by the principal investigator and approved by the Institutional Animal Care and Use Committee (IACUC). **A summary of all such exceptions is attached to this annual report.** In addition to identifying the IACUC approved exceptions, this summary includes a brief explanation of the exceptions, as well as the species and number of animals affected.

4.) The attending veterinarian for this research facility has appropriate authority to ensure the provisions of adequate veterinary care and to oversee the adequacy of other aspects of animal care and use.

CERTIFICATION BY HEADQUARTERS RESEARCH FACILITY OFFICIAL
(Chief Executive Officer (C.E.O.) or Legally Responsible Institutional Official (I.O.))
I certify that the above is true, correct, and complete (7 U.S.C. Section 2143).

SIGNATURE [redacted]

DATE SIGNED
10-27-09

APHIS FORM
AUG 2000

OCT 29 2009

Established 1923

Colorado Serum Company

October 27, 2009

Dr. Robert M. Gibbens, DVM
Director, Western Region Animal Care
USDA-APHIS-Animal Care
2150 Centre Avenue
Building B, Mail Stop #3W11
Ft. Collins, CO 80526

Dear Dr. Gibbens:

Enclosed please find our Research Facility Annual Report (APHIS Form 7023).

Supplementary documents are included which were enclosed with the 2008 Annual Report.

1. "Investigation into Guinea Pig Analgesia.......... ", dated November 11, 2004.
2. "Investigation by Colorado Serum Company's IACUC into Hamster Analgesia...... ", dated July 22, 2004.

In each case we have found no new information which would allow a change in our approach to these issues.

We have also enclosed a description of "Symptoms in Control Guinea Pigs after Challenge with *Clostridium chauvoei*", dated December 19, 2004.

Throughout this year, provisions in 9CFR 117.4(e) were applied to all animals in tests conducted according to 9CFR 113.451(b), 9CFR 113.106(c), 9CFR 113.101(c), 9CFR 113.102 (c), 9CFR 113.103(c), 9CFR 113.104(c) and 9CFR 113.107(c).

In each test, guinea pigs or hamsters that showed clinical signs of illness due to the test were humanely destroyed if the illness progressed to the point when death was certain to occur without therapeutic intervention.

Please contact us if there are any questions.

Sincerely,

[redacted]

Enclosure

COLORADO Veterinary Products

[redacted] • P.O. Box 16428 • Denver, Colorado 80216-0428
[redacted]

OCT 29 2009

www.coloradoserum.com

U.S. Vet. License No. 188

Colorado Serum Company
License No. 84-R-0007

For FY 2008-2009 Annual Report of Registered Research Facility:

Each animal in each referenced test was employed in testing a Licensed Veterinary Biological product as required by Federal Regulations as codified in Title 9CFR. Humane euthanasia of animals on 9CFR required tests is provided for in 9CFR 117.4(e) - Animals at Licensed Establishments - Test Animals (filed August 19, 1995). This is included as standard testing protocol. While limiting the duration of the pain and distress, it does not fulfill the description of use as described treatment in Section D of APHIS Form 7023, Page 1. Therefore, we feel obligated to continue to include these test animals in Section E.

Attending veterinarians and related employees have been informed and Colorado Serum Company is complying with this provision.

Explanation of usage of animals listed in Column E.

Line Item 6. Guinea Pigs

1238 guinea pigs were tested causing pain, for which no anesthetic, analgesics, or tranquilizers could be used. A detailed breakdown of guinea pigs by test reference is provided.

9CFR 113.451(b) Tetanus Antitoxin potency testing - 997 guinea pigs

Humane endpoints were addressed for all animals on this test, thus reducing the duration of pain and suffering.

9CFR 113.106(c) Clostridium Chauvoei Bacterin potency testing - 218 guinea pigs

Humane endpoints were addressed for all animals on this test, thus reducing the duration of pain and suffering.

9CFR 113.107(c) Clostridium Haemolyticum Bacterin potency testing - 23 guinea pigs

Humane endpoints were addressed for all animals on this test, thus reducing the duration of pain and suffering.

OCT 2 9 2009

Colorado Serum Company
License No. 84-R-0007

Line Item 7 Hamsters

388 hamsters were tested causing pain, for which no anesthetics, analgesics, or tranquilizers could be used. A detailed breakdown of hamsters by test referenced is provided.

9CFR 113.101(c) Leptospira Pomona Bacterin potency testing - 111 hamsters

The 111 hamsters included required controls for potency tests [9CFR 113.101(c)(2)], plus necessary hamsters required to establish the validity of the challenge dose (10 to 10,000 hamster LD_{50}) by titration. 9CFR 113.101 (c)(3).

For all test hamsters, Colorado Serum Company specifically has applied provisions of 9CFR 117.4(e) wherein ill animals were humanely euthanized upon reaching a point where death was expected to occur.

9CFR 113.102(c) Leptospira Icterohaemorrhagiae Bacterin potency testing - 96 hamsters

The 96 hamsters included required controls for potency tests [9CFR 113.102(c)(2)], plus necessary hamsters required to establish the validity of the challenge dose (10 to 10,000 hamster LD_{50}) by titration. 9CFR 113.102(c)(3).

For all test hamsters, Colorado Serum Company specifically has applied provisions of 9CFR 117.4(e) wherein ill animals were humanely euthanized upon reaching a point where death was expected to occur.

9CFR 113.103(c) Leptospira Canicola Bacterin potency testing - 82 hamsters

The 82 hamsters included required controls for potency tests [9CFR 113.103(c)(2)], plus necessary hamsters required to establish the validity of the challenge dose (10 to 10,000 hamster LD_{50}) by titration. (CFR 113.103(c)(3).

OCT 2 9 2009

Colorado Serum Company
License No. 84-R-0007

Line Item 7 | 9CFR 113.103(c) | Continued

For all test hamsters, Colorado Serum Company specifically has applied provisions of 9CFR 117.4(e) wherein ill animals were humanely euthanized upon reaching a point where death was expected to occur.

9CFR 113.104(c) | Leptospira Grippotyphosa Bacterin potency testing - 99 hamsters

The 99 hamsters included required controls for potency tests [(9CFR 113.104(c)(2)], plus necessary hamsters required to establish the validity of the challenge dose (10 to 10,000 hamster LD_{50}) by titration. 9CFR 113.104 (c)(3).

For all test hamsters, Colorado Serum Company specifically has applied provisions of 9CFR 117.4(e) wherein ill animals were humanely euthanized upon reaching a point where death was expected to occur.

[redacted] ______________

Oct 26 2009
Date

OCT 2 9 2009

COLORADO SERUM COMPANY

[redacted]

P.O. Box 16428 Denver, Colorado 80216-0428

November 11, 2004

Subject: Investigation by Colorado Serum Company's Institutional Animal Care and Use Committee into guinea pig analgesia and its possible usage in guinea pigs during the *Clostridium tetani* antitoxin potency tests done at Colorado Serum Company

From: [redacted]

To: Colorado Serum Company's Institutional Animal Care and Use Committee and [redacted] [redacted] Colorado Serum Company

Background: Use of guinea pigs for potency tests when developing Tetanus Antitoxin has been the requirement by USDA (9-CFR, 113.451) for many years. The potency test is essentially an in-vivo neutralization test that requires two guinea pigs each for controls and for each dilution. The two controls are injected subcutaneously with a 3-ml dose of the Standard Toxin-Antitoxin mixture. Injections shall be made in the same order that toxin is added to the dilutions of antitoxins. These shall be observed parallel with the titration of one or more unknown antitoxins. Two test guinea pigs will be used for each dilution of the unknown antitoxin (also a 3-ml dose, subcutaneously). Controls are observed until they are down and are unable to rise or stand under their own power. At this time they are humanely euthanized and the time of death is recorded in hours. For a satisfactory test, the controls must reach this point with clinical signs of tetanus within 24 hours of each other and within an overall time of 60—120 hours. The clinical signs to be observed are increased muscle tonus, curvature of the spine, asymmetry of the body outline when the resting animal is viewed from above, generalized spastic paralysis, particularly of the extensor muscles, inability to rise from a smooth surface when the animal is placed on its side, or any combination of these signs. If the control guinea pigs do not respond in this manner the entire test shall be repeated. Potency of an unknown antitoxin is determined by finding the mixture which will protect the test animal the same as the Standard Toxin-Antitoxin mixture. Test animals dying sooner than the controls indicate the unit value selected in that dilution was not present, whereas those living longer indicate a greater unit value.

Analgesia in Guinea Pigs: An on-line and textbook search conducted by Colorado Serum Company's IACUC [redacted] revealed a variety of drugs used in guinea pigs after surgical procedures. The Duke University IACUC website had a very thorough

OCT 29 2009

THE PEAK OF QUALITY

U.S. VETERINARY LICENSE NO. 188

guideline for analgesia in all rodents and a variety of drugs and routes of administration based on the degree of pain and discomfort of a given procedure (see attached). Injectable analgesic options for guinea pigs include Codeine, Nalbuphine or Morphine given every 4 hours or Buprenorphine every 12 hours beginning just before symptoms are estimated to begin. Oral medications include Aspirin or Phenylbutazone added to the drinking water once daily, however once guinea pigs begin to show signs of tetanus it can be expected that their water consumption will diminish greatly and that once they are prostrate they will physically be unable to stand and drink. At the later stages of tetanus they are unable to swallow. Euthanasia hopefully is done before the late stages of disease but unfortunately death is sometimes the outcome with the test pigs before the study investigator can officially call the test over, if they have a lower level of antibody protection when compared to the Standard Controls.

Conclusion: There is obviously a direct conflict between animal welfare issues (pain and suffering) and this neutralization potency test that is required by USDA-APHIS-CVB for each new serial of tetanus antitoxin. The dilemma is that the test relies solely on symptoms of tetanus in relation to the Standard Controls as described above, in essence pain and suffering are components of the measured parameters of the test and giving any drugs to alter or help alleviate the symptoms will affect the results of the test. Currently the best welfare that can be provided is humane euthanasia once the symptoms have reached the point that the study investigator can interpret the test and give the staff veterinarian the o.k. to intervene and euthanize.

Due to this direct conflict, Colorado Serum Company's Institutional Animal Care and Use Committee has determined that there is no practical way to intervene with pain medications during the Tetanus Antitoxin potency neutralization test without altering the animal symptoms and thus altering the interpretation of the test. The only solution to this is to replace the guinea pig test with an in-vitro test (which Colorado Serum Company would be greatly in favor of). Currently there is a Competitive Elisa Test for Tetanus Antitoxin that has been tried but unfortunately it has not shown consistent, comparable results when run in parallel with the guinea pig neutralization test.

[redacted]

November 12, 2004

OCT 29 2009

Animal Care & Use Program

DLAR
Excellence in Animal Care and Use

IACUC

OAWA

Important Deadlines: New Protocols: Dec. 1, 2004 | Amendments: Nov. 10, 2004 | IACUC Meeting: Nov. 10, 2004

SPECIAL PROGRAM NOTE: HOLIDAY ANIMAL ORDER DEADLINES - CLICK HERE!

Animal Diseases
Animals on the Web
Program Contacts
References
Reporting Concerns
EMERGENCIES

Guidelines for Rodent Analgesia

- Because the anatomic structures and neurophysiologic mechanisms leading to the perception of pain are similar in humans and non-human animals, it is reasonable to assume that if a stimulus:
  - is painful to humans,
  - is damaging or potentially damaging to tissues, or
  - induces escape and emotional responses in an animal, it must be considered to be painful to that animal.

The choice of post-surgical analgesic drug must take into consideration (and be appropriate for) the estimated level and duration of post-surgical/post-procedural pain/discomfort associated with the specific surgery/procedure.

Generally speaking, analgesics should be provided as early in the exercise as possible. Providing analgesic PRIOR to the initiation of the painful stimulus is most preferred; providing analgesia during the procedure (of an anesthetized animal) is considered the minimal standard when pre-procedural analgesia is not possible.

This concept of pre-emptive analgesic is important to the stability of the research

OCT 2 9 2009

animal and the outcome of the research data. An additional benefit of pre-emptive analgesia is that the amount of anesthetics required for proper anesthesia are often reduced, further benefiting the animal with a rapid return to normal, while saving critical research dollars.

For analgesic drugs that are administered via the oral route, the drug must:

- be administered via oral gavage at the appropriate dosing frequency, or
- if administered via the drinking water, the drug must be placed in the drinking water starting a minimum of 7 days prior to the surgery/painful procedure in order for the animal to be "exposed" and, presumably, adapted to the altered taste of the water at the time of the surgery/procedure. This prepatory step is necessary to overcome "neophobia," a behavioral adaptation of rodents (especially rats) whereby they may not consume adequate quantities of fluids with a new taste sensation is recognized. Placing a flavored analgesic in the water post procedure may allow for association of the "pain" with the new flavor and thereby rejection of the flavored water resulting in inadequate analgesia.

Duke utilizes a tiered system of analgesic delivery, based upon generalized observations of degrees of pain or discomfort. These include:
P1: Survival Surgery/Survival Procedures Estimated to Cause MARKED Post-Surgery/Post-Procedural Pain
P2: Survival Surgery/Survival Procedures Estimated to Cause MODERATE Post-Surgery/Post-Procedural Pain
P3: Survival Surgery/Survival Procedures Estimated to Cause MILD Post-Surgery/Post-Procedural Pain

P1: Survival Surgery/Survival Procedures Estimated to Cause MARKED Post-Surgery/Post-Procedural Pain. (Examples: any surgery involving thoracotomy or celiotomy; transplantation of an organ; limb amputation; extensive orthopedic surgery; cecal ligation and puncture):

Drug	Mouse	Rat	Hamster	Gerbil	Guinea Pig
Morphine	10 mg/kg SQ or IM every 4	10 mg/kg SQ or IM every 4	10 mg/kg SQ or IM every 4	10 mg/kg SQ or IM every 4	10 mg/kg SQ or IM every 4

OCT 2 9 2009

	hours	hours	hours	hours	hours
Codeine	20 mg/kg SQ every 4 hours	50 mg/kg SQ every 4 hours			40 mg/kg SQ every 4 hours
Oxymorphone	0.15 mg/kg SQ or IM every 4 hours	0.22-0.33 mg/kg SQ every 4 hours	0.2-0.5 mg/kg SQ or IM every 6 hours	0.2-0.5 mg/kg SQ or IM every 6 hours	

P2: Survival Surgery/Survival Procedures Estimated to Cause MODERATE Post-Surgery/Post-Procedural Pain: (Examples: most laparoscopic (i.e. intra-abdominal) surgeries, limited orthopedic surgery, intra-cranial surgery):

Drug	Mouse	Rat	Hamster	Gerbil	Guinea Pig
Meperidine	20 mg/kg SQ or IM every 3 hours	20 mg/kg SQ or IM every 3 hours	20 mg/kg SQ or IM every 3 hours	20 mg/kg SQ or IM every 3 hours	
Nalbuphine	4-8 mg/kg SQ or IM every 3 hours.	2-5 mg/kg SQ every 4 hours	4-8 mg/kg SQ or IM every 3 hours	4-8 mg/kg SQ or IM every 3 hours	1-2 mg/kg IM every 4 hours
Pentazocine	10 mg/kg SQ every 4 hours	10 mg/kg SQ every 4 hours			
Butorphanol	2 mg/kg SQ every 4 hours	5 mg/kg SQ every 4 hours	5 mg/kg SQ every 4 hours	5 mg/kg SQ every 4 hours	

OCT 2 9 2009

Buprenorphine	0.05-0.1 mg/kg SQ every 12 hours	0.1-0.5 mg/kg SQ every 12 hours	0.5 mg/kg SQ every 8 hours	0.2 mg/kg every 8 hours	0.05 mg/kg SQ every 12 hours
Carprofen	5 mg/kg SQ every 12 hours	5 mg/kg SQ every 12 hours			
Aspirin	300 mg/kg orally every 24 hours	100 1 mg/kg orally every 24 hours			86 mg/kg orally every 24 hours
Ibuprofen	7.5 mg/kg orally every 24 hours	30 mg/kg orally every 24 hours			
Phenylbutazone	30 mg/kg orally every 24 hours	20 mg/kg orally every 24 hours			40 mg/kg orally every 24 hours
Acetaminophen	300 mg/kg orally every 24 hours	300 mg/kg orally every 24 hours			

OCT 29 2009

P3: Survival Surgery/Survival Procedures Estimated to Cause MILD Post-Surgery/Post-Procedural Pain. (Examples: Placement of subcutaneous tumors or subcutaneous osmotic pumps; placement of chronic indwelling vascular cannulaes; orchiectomy)

Drug	Mouse	Rat	Hamster	Gerbil	Guinea Pig
Flunixin			2.5 mg/kg IM every 12 hours	2.5 mg/kg IM every 12 hours	
Aspirin	300 mg/kg orally every 24 hours	100 mg/kg orally every 24 hours			86 mg/kg orally every 24 hours
Ibuprofen	7.6 mg/kg orally every 24 hours	30 mg/kg orally every 24 hours			
Phenylbutazone	30 mg/kg orally every 24 hours	20 mg/kg orally every 24 hours			40 mg/kg orally every 24 hours
Acetaminophen	300 mg/kg orally every 24 hours	300 mg/kg orally every 24 hours			

SQ=subcutaneous
IM=intramuscular

For purposes of administering a drug via the drinking water:

OCT 2 9 2009

	Rat	Mouse	Hamster	Gerbil	Guinea Pig
Normal* Daily Water Consumption	8-11 ml/100 gm body weight/day	15 ml/100 gm body weight/day	30 ml/day	4-7 ml/100gm body weight/day	10 ml/100 gm body weight/day

*Animals that have been subjected to a painful procedure/surgery will not drink the "normal" amount of water for a minimum of 24 hours post-surgery/post-procedure. It is estimated that normal water consumption will be reduced by at least 50%.

Amount of orally administered drug to mix/administer per ml of drinking water:

Drug	Rat-Normal Water Consumption	Rat 50% Decrease in Water Consumption	Mouse Normal Water Consumption	Mouse 50% Decrease in Water Consumption	Guinea Pig Normal Water Consumption	Guinea Pig 50% Decrease in Water Consumption
Aspirin	0.9 mg/ml	1.8 mg/ml	2 mg/ml	4 mg/ml	0.9 mg/ml	1.8 mg/ml
Ibuprofen	0.3 mg/ml	0.6 mg/ml	.05 mg/ml	0.1 mg/ml		
Phenylbutazone	0.2 mg/ml	0.4 mg/ml	0.2 mg/ml	0.4 mg/ml	0.4 mg/ml	0.8 mg/ml
Acetaminophen	2.7 mg/ml	5.4 mg/m	2.0 mg/ml	4.0 mg/ml		

Local Anesthetics: Even with general anesthesia, the topical, subcutaneous (at surgical incision site), intra-articular, etc. administration of a local anesthetic is recommended in order to provide additional post-surgical analgesia. Local anesthetics should not be used alone to provide post-surgical/post-procedural analgesia.

Agent	Potency (Procaine=1)	Onset	Duration*	Topical Use	Infiltration Use	Nerve Block Use
Procaine (Novacaine)	1	Slow	30-90 minutes	-	1-2%	1-2%
Chloroprocaine (Nesacaine)	2.4:1	Fast	Up to 2 hours	-	1-2%	1-2%
Lidocaine (Xylocaine)	2:1	Fast	2 hours	2-4%	0.5-2%	0.5-2%
Mipivacaine (Carbocaine)	2.5:1	Fast	2-4 hours	-	1-2%	1-2%
Tetracaine	12:1	Slow	3-8 hours	0.2%	0.1%	0.1%

2010-11-16 - 000040

OCT 2 9 2009

[illegible]						
[illegible]	1:2	Fast	3-6 hours	5%	0.5-1%	2%
[illegible]	1:1	Intermediate	[illegible] hours		0.25%	0.5%

[illegible] Duration in rodents (with their higher metabolic [illegible]

About Duke | News | Research | Events Calendar | Libraries

Protocol Approval | Animal Care Question | Home Page | Reporting an Animal Welfare Concern

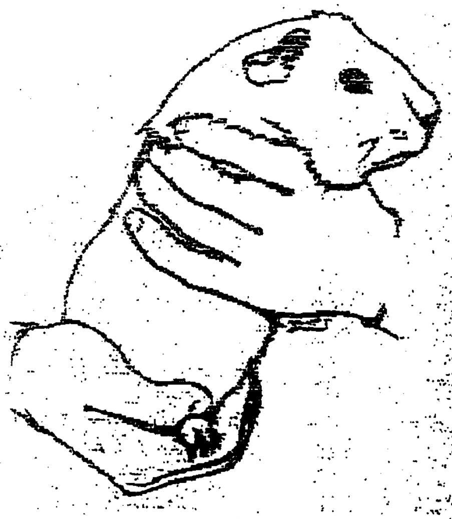

Guinea Pig

Compiled by
Dr. James B. Nichols, University Veterinarian
Florida Atlantic University

30 May 2003

LABORATORY GUINEA PIG

Cavia porcellus

Background

The wild cavy or guinea pig occurs in South America from Columbia and Venezuela southward to Brazil and northern Argentina. Guinea pigs inhabit a wide variety of habitat such as rocky regions, savannas, the edge of forests and swamps. In the wild forms the hair is fairly course and long but in the domestic guinea pigs it varies from smooth and short to smooth and long to course and short in some for-ms the hair even radiates in rosettes. Although domestic guinea pigs exhibit an extremely wide range of color, the wild forms are generally gray or brownish. Guinea pigs have stocky bodies, fairly short hind legs, short ears and lack a tail. The hind feet are long with sharp claws. Guinea pigs in the wild are burrowing animals. Sometimes they live in the abandoned burrows of other animals. They generally associate in small groups, usually 5 to 10 individuals, and are always prepared to flee at the slightest indication of danger. They are nocturnal feeders and the diet consists of various forms of vegetation. South American native Indian tribes, particularly the Incas, raised them for food since their flesh is of excellent quality. During the second world war a project conducted by the Navy investigating the

OCT 2 9 2009

2010-11-16 - 000042

palatability and nutritional value of various laboratory species for the human population determined that the Guinea Pig was the most desirable of the common species. The Guinea Pig was introduced into Europe in the 16th Century by 1 the Dutch and their use spread quickly throughout the Continent. They were known by a variety of common names: "Sea Pig," "Barbary Rabbit," or "Little Sea Pig". The name Guinea Pig has become synonymous with of "experimental subject" in the that English language.

The unique characteristics of the Guinea Pig that differ from other experimental animals include the highly developed young at birth due to the long gestation period and the udder type mammary gland. The young at birth are fully developed. Their eyes are open, they are fully haired, and completely mobile. They begin eating solid food at approximately 2 to 3 days of age and can be easily weaned at 10 days. The mammary gland consists of a two section udder, each section with a single teat. Milking machines have been easily developed to utilize this udder for milk secretion research studies. The Guinea Pig is unable to synthesize an adequate supply of vitamin C and therefore must be supplied with either a supplement or a fortified diet.

Handling techniques

Guinea Pigs are probably the easiest of all the common laboratory animals to handle and restrain. They are nervous, high-strung animals compared to other laboratory rodents and are rather noisy. These sounds consist mainly of squealing, whistling, and grunting sounds. To restrain a Guinea Pig, grasp the animal around the thoracic area from above with the thumb and forefinger right behind the front legs and lift. The other hand should be placed under the Guinea Pig's hind quarters for support. Failure to restrain and support the hind quarters may result in injury to the Guinea Pig as well as injuries to the operator from scratching. Guinea Pigs rarely if ever bite. Since they have short legs and heavy bodies they are unable to climb or jump. In some instances they are housed in open-top cages or pens because of this inability to escape. IP injection of the Guinea Pig is accomplished by restraining the animal as indicated above and injecting in one of the lower quadrants of the abdomen. Subcutaneous injection can be accomplished by lifting the loose skin of the back, particularly over the neck and shoulder, and injecting under the f o I d . The Guinea Pig may be bled by heart puncture, anterior vena cava puncture, or small samples may be obtained from the medical canthus of the eye. Repeated blood sampling utilizing these methods can be accomplished if a suitable period of two or three days between drawings is allowed. In males the lateral vein the penis may be used for IV injections.

Experimental Uses

As the common usage of the term Guinea Pig implies, this species is widely employed as a research animal. The Guinea Pig has been used in antibody production, tumorigenesis, nutrition, genetics, radiation research, and dental studies including antibacterial action of saliva and production. The Guinea Pig is used extensively in laboratory studies of disease, nutrition and heredity, and in, development of sera. The Guinea Pig has been widely employed in biomedical research since 1780. Lavoisier used the cavy for measuring heat production. Much of the work relating to the discovery of Vitamin C, the diagnosis of tuberculosis, and the research on anaphylaxis has been performed in this species.

Strains and Sources

The majority of Guinea Pigs used in this country are random bred Guinea Pigs. Although inbreeding of Guinea Pigs was initiated at the Bureau of Animal Industry facility near Washington, D.C., in 1897, only two inbred strains, 2 and 13, still exist and these are not widely used,. The Guinea Pig, because of its large size and caging requirements, is not as widely used as the other laboratory species but does lend itself as an animal model to many research studies. When selecting a particular inbred or many research

OCT 2 9 2009

studies. When selecting a particular inbred or random bred strain of Guinea Pig for utilization, the availability and source should be closely considered. Because of its limited use, only a small number of breeders supply Guinea Pigs. The investigator is encouraged to investigate fully the suggested commercial sources before utilizing the animals these sources. As the quality of the animal received and supplied may vary greatly from one supplier to another, once a particular strain and supplier is selected they should be utilized throughout the entire study. Strain differences between individual breeders may be considerable. Therefore one supplier should be used if possible.

PHYSIOLOGICAL AND GENERAL DATA	
Breeding season	no definite season
Estrous cycle	16 ½ days (range of 16 to 19 days)
Estrous duration	6 to 11 hours
Gestation period	63 days (range of 59 to 72)
Birth weight	70 - 100 grams
Litter size	2 to 5
Weaning weight	150-200 grams (14-21 days)
Male breeding age	3 to 4 months
Female breeding age	2 to 3 months
Adult Male weight	1000 to 1200 grams
Adult female weight	850 to 900 grams
Male reproductive life span	4 years
Female reproductive life span	1.5 to 4 years (4-5 litters)
Body temperature	39.1°C (range of 38.4 to 39.8)
Diploid number	64
Respiration rate	90 per minute
Food consumption	6 grams of feed/100 grams of body weight/day
Water consumption	10 ml/100 grams of body weight/day
GI transit time	13-30 hours
Heart rate	280 (range 260 to 400)

Guinea Pig

Updated 08/26/2003

Anesthesia/Analgesia	Anticholinergic	Antiparasitic	Euthanasia
Anti-inflammatory	Antibiotics	Miscellaneous	Notes

ANESTHETIC, ANALGESIC		Back to Top
ACETAMINOPHEN (TYLENOL)	269 MG/KG IP *7	NONSTEROIDAL ANTI-INFLAMMATORY
ACETHYLSALICYLICATE ACID (ASPIRIN)	269 IP/300 PO/20 SC MG/KG	NONSTEROIDAL ANTI-INFLAMMATORY
ACETYLPROMAZINE (ACEPROMAZINE) - DOSAGE	1-2 MG/KG IM	TRANQUILIZER-PHENOTHIAZINE
BUPIVACAINE (MARCAINE)	AS NEEDED	LOCAL ANESTHETIC

OCT 29 2009

BUPRENORPHINE (BUPRENEX)	0.05 MG/KG SC* Q8-12H**/****	NARCOTIC AGONIST-ANTAGONIST V
CHLORAL HYDRATE 5%	200-400 MG/KG IP	HYPNOTIC
DIAZAPAM (VALIUM) DOSAGE	2.5 MG/KG IP *	TRANQUILIZER - BENZODIAZEPINE IV
DIAZAPAM - DURATION TO EFFECT	1-2 HOURS	TRANQUILIZER - BENZODIAZEPINE IV
DIAZAPAM - TIME TO EFFECT	2-5/5 MIN	TRANQUILIZER - BENZODIAZEPINE IV
DOXAPRAM -DOPRAM V-DOSAGE	5MG/KG IV***	ANALEPTIC
FENTANYL/DROPERIDOL (INNOVAR-VET)-DOSAGE	0.66-0.88 ML/KG IM * (CAUTION)	NEUROLEPTANALGESIC, NARCOTIC/BUTYROPHENONE TRANQUILIZER II
FENTANYL/DROPERDOL - DURATION EFFECT	20-40 MIN	NEUROLEPTANALGESIC, NARCOTIC/BUTYROPHENONE TRANQUILIZER II
FENTANYL/DROPERIDOL - TIME TO EFFECT	15/5 MIN	NEUROLEPTANALGESIC, NARCOTIC/BUTYROPHENONE TRANQUILIZER II
FENTANYL/DROPERIDOL/DIAZEPAM-DOSE	1 ML/KG IM/2.5 MG/KG IP *	NEUROLEPTANALGESIC; NARCOTIC/BUTYROPHENONE TRANQUILIZER,BUTYROPHENONE II
KETAMINE (KETASET, VETALAR) - DOSAGE	50-200 MG/KG IM *	DISSOCIATIVE
KETAMINE-ACEPROMAZINE	125/5 MG/KG IM*/**	DISSOCIATIVE/TRANQUILIZER
KETAMINE-XYLAZINE (ROMPUN)	40-100 IM/4-5 SC IM MG/KG/ OR 87/13 MG/KG IM*	DISSOCIATIVE/ANALGESIC
KETAMINE-DIAZEPAM (VALIUM)	100/5 MG/KG IM*/**	DISSOCIATIVE/TRANQUILIZER
MEPERIDINE (DEMEROL)	20 MG/KG IP/IMSC Q2-3H	NARCOTIC II
METHOHEXITAL (BREVITAL/BREVANE)	31 MG/KG IP **	ULTRA SHORT BARBITUATE IV
MORPHINE	10 MG/KG IM SC **Q2-4H**	NARCOTIC II
PENTAZOCINE (TALWIN-V)	10 MG/KG SC IM IV	NON-NARCOTIC ANALGESIC IV
PENTOBARBITAL Na (NEMBUTAL)	25-40 MG/KG IM IP*	BARBITUATE SHORT II
PHENCYCLADINE - DOSAGE	3 MG/KG IM:	TRANQUILIZER
PHENCYCLADINE - DURATION	20-60 MIN	TRANQUILIZER
PHENCYCLADINE - TIME EFFECT	5-10 MIN	TRANQUILIZER
PHENCYLADINE - TIME FULL RECOVERY	2 HOURS	TRANQUILIZER
PHENYLBUTAZONE (BUTAZOLDIN)	150 MG/KG IP *7	NONSTEROIDAL ANTI-INFLAMMATORY
THIAMYLAL (SURITAL)	20-50 MG/KG IV;IP	BARBITUATE ULTRASHORT III
THIOPENTAL (PENTOTHAL)	20-55 MG/KG IV IP // 20 IV ****	BARBITUATE ULTRASHORT III
XYLAZINE (ROMPUN)- DOSAGE	3-5 MG/KG IM	ALPHA-2-ADRENERGIC AGONIST SEDATIVE, ANAGESIC, MUSCLE RELAXANT
ANESTHETIC GAS		**Back to Top**
HALOTHANE - MAC	0.95	INHALANT
ISOFLURANE - MAC		INHALANT
METHOXYFLURANE - MAC	0.22	INHALANT
NITROUS OXIDE-MAC	150	INHALANT
ANTI-INFLAMMATORY		**Back to Top**
DEXAMETHASONE (AZIUM)	0.06 MG SC IM IV IP	STEROID
PREDNISONE (METICORTIN)	0.05-0.22 MG SC IM	STEROID
ANTIBIOTIC		**Back to Top**
AMOXICILLIN	TOXIC**	ANTIBIOTIC

OCT 29 2009

AMPICILLIN (POLYFLEX)	TOXIC**/***	BETA LACTAM ANTIBIOTIC
BACITRACIN	NEVER	ANTIBIOTIC
CEPHALCRIDINE (LORIDINE)	25 MG/KG IM Q24H**/***	BETA LACTAM ANTIBIOTIC
CHLORAMPHENICOL PALMITATE (CHLOROMYCETIN)	50 MMG/KG PO TID ***	BACTERIAL STATIC BROAD SPEC.
CHLORAMPHENICOL SUCCINATE (CHLOROMYCETIN)	20 MG/KG IM Q24H **/***	BACTERIAL STATIC BROAD SPEC.
ERYTHROMYCIN	NEVER	BACTERIAL
GENTAMICIN (GENTOCIN)	5 MG/KG SC IM SID *** 14D*	AMINOGLYCOSIDES
GRISEOFULVIN (FULVICIN U/F)	25 MG/KG PO IN FOOD ***	ANTIFUNGAL
NEOMYCIN (BIOSOL)	10 PO/ 30 SC MG/KG Q24H **// OR 5 PO Q12H/30 SC Q24H MG/KG ***	DIARRHEA
OXYTETRACYCLINE (LIQUAMYCIN)	TOXIC **/***	BACTERIAL STATIC BROAD SPEC.
PENICILLIN	NEVER	ANITBIOTIC
PENICILLIN - BENZATHIN/PROCAINE(BICILLIN)(FLOCILLIN)		BACTERIAL
STREPTOMYCIN (BIOTEC)	NEVER	BACTERIAL
SULFAMETHAZINE OR SULFAMERAZINE	DW 1 MG/ML ***	BACT. COCCIDIA,CIT,B
SULFAQUINOXALINE (SULQUIN)	DW 1 MG/ML ***	EIMERIA,KLOS. PAST.
TETRACYCLINES	TOXIC ***	BACTERIAL STATIC BROAD SPEC//FROG RED LEG
TRIMETHOPRIM (40MG/ML)/SULPHADOXINE (200MG/ML) (TRIBRISSEN)	0.5ML/KG SC**// OR 30 MG/KG SC Q24H (YOUNG 15 MG/KG SC Q24H)***	TRIMETHOPRIM / SULPHONAMIDE
TYLOSIN	66 MG/L 21D	MACROLIDES
ANTICHOLINERGIC		Back to Top
ATROPINE - DOSAGE	0.05 MG/KG SC IM**/***	ANTICHOLINERGIC
ATROPINE - DURATION EFFECT	15 MIN	ANTICHOLINERGIC
ATROPINE - TIME TO EFFECT	10 MIN	ANTICHOLINERGIC
ANTIPARASITIC		Back to Top
CARBARYL POWDER (DIRYL)	5 D	ECTOPARASITIC
DICHLORVOS (TASK)	500 MG/KG IN FEED/24H	ECTOPARASITIC-SYPH, HETER
DICHLORVOS (VOPONA STRIPS)	STRIP 48H/WK // OR 1/5 OVER CAGE FOR 3D***	ECTOPARASITIC
FURANACE	NEVER	BACTERIAL
IVERMECTIN (IVOMEC)	200 MCG/KG PO SC***	ANTI-NEMATODES/ECTOPARASITIC
MEBENDAZOLE (TELMIN)	10 MG/KG PO 5D***	NEMATODIASIS COSMOCE
NICLOSAMIDE (YOMESAN)	100 MG/KG PO***	CESTODES-HYMENOLEPSIS
PIPERAZINE	100 MG/KG PO***//3 MG/ML IN WATER + SYRUP *9	ANTI-NEMATODES-SYPHACIA & HET & PINWORMS
SUCCINYLSULFATHIAZOLE	1% IN WATER	EIMERIA
THIABENDAZOLE (TBZ; OMIZOLE)	100 MG/KG PO 5D***	ANTI-NEMATODES - OPHIDAS
HORMONE		Back to Top
OXYTOCIN	0.2-3 UNITS/KG SC IM *9	UTERINE/MILK HORM.
EUTHANASIA	Dose	Comments Back to Top
Sodium pentobarbital	150 mg/kg IP	Controlled substance
Halothane	To effect	High Concentration, Rapid flow
Isoflurane	To effect	High Concentration, Rapid flow
CO2	To effect	Requires some other method to ensure death

OCT 2 9 2009

CO	To effect	See hazards under euthanasia training
Potassium chloride	1-2 mmol/kg IV IC	Requires general anesthesia
Cervical dislocation	Unacceptable	Too much musculature
Decapitation	Conditionally acceptable	Requires scientific justification by user and approval of IACUC, Requires special training and monitoring, Hazard, requires special training for hazard, Equipment has to be maintained; Only on very young.
Chloral hydrate	Unacceptable	Not used in Guinea Pigs
Notes		Back to Top

*= Anesthesia and Analgesia in Laboratory Animals: American College of Laboratory Animal Medicine: 1990
** = Laboratory Animal Anesthesia: Flecknell: 1989
*** = Drug Dosages for Small Mammals: McKellar: 1989
****= Recognition and Alleviation of Pain and Distress in Laboratory Animals: NRC: 1992
*5= Current Veterinary Therapy 2 Food Animal Practice
*6 = University of Calif., San Fran., Morrish
*7 = Basic Care of Experimental Animals
*8= Therapeutic Guide and Anesthesia, O'Hamdley
*9= The Biology and Medicine of Rabbits and Rodents
*10= ???????? Continuing Education Vol. 5, No. 4, April 1992
*11 = USUHS Formulary
*12 = LAB ANIMAL OCT 91 PAGE 34
*13= ANESTHESIA AND ANALGESIC DOSES, SCHAEFFER, KNOXVILLE, TN

OCT 29 2009

COLORADO SERUM COMPANY

[redacted] P.O. Box 16428 Denver, Colorado 80216-0428

July 22, 2004

Subject: Investigation by Colorado Serum Company's Institutional Animal Care and Use Committee into hamster analgesia and its possible usage in hamsters during the *Leptospira* challenge portion of *Leptospira* potency tests at Colorado Serum Company.

From: [redacted] Colorado Serum Company IACUC Members.

To: Colorado Serum Company Institutional Animal Care and Use Committee and [redacted] [redacted] Colorado Serum Company.

Background: Use of hamsters for potency tests when developing Leptospira organisms for bacterin production has been the requirement by USDA (9-CFR, 113.101 – 113.105) for many years. Ten vaccinates and 10 or more controls are challenged intraperitoneally with a suspension of virulent Leptospira organisms. This applies to potency tests for *Leptospira pomona, Leptospira icterohaemorrhagiae, Leptospira canicola* and *Leptospira grippotyphosa* fractions. If 8 or more controls die from leptospirosis during a 14 day post challenge observation period then the test is considered valid. The degree of pain and suffering to the control hamsters is of concern for animal welfare reasons and an alternative in-vitro test that has reproducible and comparable results to the in-vivo potency test would be a much better alternative replacement test. Until that happens all biologic companies producing *Leptospira* bacterins will continue to use hamster in-vivo potency tests.

The degree of pain and suffering the control hamsters endure during this 14 day observation period is a subjective evaluation given the fact that hamsters cannot communicate with humans. Based on the disease syndrome of Leptospirosis in our domestic species the clinical signs can be mild to severe. In most animal species the disease causes septicemia, nephritis, depression and anorexia. It is safe to assume that hamsters suffer from many of these symptoms as well, and it is most likely not a chronic pain as much as it is a general discomfort or sick feeling that they are experiencing. Parameters of hamster behavior used to measure discomfort or pain would include exploration, grooming and posture as well as food and water consumption and fur quality.

Analgesic use in hamsters: An on-line and textbook search conducted by Colorado Serum Company's IACUC [redacted] revealed very few drug options for analgesia in hamsters (see attached documents). Buprenorphine was the most common analgesic for hamsters cited. The labeled route is by subcutaneous injection and the

OCT 29 2009

frequency is every 8 hours. In one published paper (see attached) buprenorphine was used in the syngeneic murine tumor model to investigate whether the pain or discomfort in mice with these tumors could be reduced with this analgesic via an oral route. In this study they found no significant difference in pain or discomfort between the buprenorphine treated group and the control group based on their scoring system. Even though this experiment was done with mice it would be plausible to compare the discomfort or pain from multiple tumors to that which would be experienced by hamsters during a systemic leptospirosis infection. Also the process of handling these hamsters and giving them injections 2 – 3 times daily would itself be stressful and painful. There would be a significant zoonotic concern for the person involved giving the injections to multiple infected hamsters from the urine as well as an increased chance of being bitten considering you are giving injections to sick and uncomfortable hamsters 2 to 3 times daily for up to 14 days. Treating the symptoms without treating the source of the disease with antibiotics (which can't be done for obvious reasons) is also of questionable value. There would also be concern as to what effect this analgesic would have on the outcome of the potency test. All drugs are processed and eliminated by the liver and kidneys and since these are two target organs of *Leptospira* organisms, toxic levels of buprenorphine could buildup in a *Leptospira* infected hamster and could alter the time of death or even contribute to a death that might not otherwise happen - thus affecting the outcome of the potency test and possibly resulting in a falsely valid test. Other drugs mentioned are injectable also and do not offer anything unique or superior to buprenorphine.

The Cornell website detailed an oral preparation of buprenorphine in jello for use in mice (see attached). Whether this would work in hamsters is not known. One concern is the fact that anorexia is a very common symptom with Leptospirosis and depending on the degree of anorexia and the fact that these hamsters are usually housed 5 per cage, some hamsters may be getting their medication and some may not - due to anorexia or competition. This could also cause some hamsters to overdose on buprenorphine if they all aren't eating the same amount of jello.

Conclusion:
Colorado Serum Company's IACUC has determined that no analgesia we have found can realistically be expected to relieve an infected hamster from the symptoms of Leptospirosis especially without concurrent use of antibiotics to fight the etiologic agent (*Leptospira*) since this would defeat the purpose of the potency study. Buprenorphine works well with acute, sharp post-surgical type pain and would be of very questionable value in hamsters suffering from discomfort associated with a systemic infection. The best course of action in our opinion would be to replace the hamster in-vivo potency test with an in-vitro test that does not require animal testing for potency.

[redacted]

OCT 2 9 2009

84R0007
1086

December 29, 2004

To: [redacted]
From: [redacted]

Subject: Symptoms in control guinea pigs after challenge with *Clostridium chauvoei* and *hemolyticum* during *Cl. chauvoei* and *hemolyticum* bacterin in vivo potency tests.

The following are symptoms that I have personally observed during this potency test. I will also include some more detailed textbook description of these symptoms.

The potency tests for both of these fractions involves a 3 day observation after IM challenge with ~100 LD_{50} of live bacteria (*Cl. chauvoei or hemolyticum*). Within 24 to 48 hours the first symptoms of disease begin to appear. Lethargy, anorexia, stiffness with reluctance to move, sero-purulent discharge from the festering injection site, and vocalization (associated with pain) when handled are frequent symptoms most commonly seen. The swelling at the injection site is hot and painful at first and eventually becomes cold and necrotic as the infection becomes systemic. A high fever is also associated with these clostridial diseases. Once symptoms are obvious the disease progresses quickly and death can happen rapidly. Once obvious symptoms are observed, veterinary intervention and humane euthanasia is implemented whenever possible.

[redacted]

OCT 2 9 2009

United States Department of Agriculture
Animal and Plant Health Inspection Service

TTHOMPSON
2381012563102O1 insp_id

Inspection Report

COLORADO SERUM COMPANY

Customer ID: **1086**
Certificate: **84-R-0007**
Site: 001
COLORADO SERUM COMPANY

P. O. BOX 16428

DENVER, CO 80216

Type: ROUTINE INSPECTION
Date: Aug-26-2010

3.28 (a) (4)
PRIMARY ENCLOSURES.

<<All primary enclosures for guinea pigs and hamsters shall conform to the following requirements: Primary enclosures equipped with mesh or wire floors shall be so constructed as to allow feces to pass through the spaces of the mesh or wire: Provided, however, That such floors shall be constructed so as to protect the animals' feet and legs from injury.>>

On review of the daily records for guinea pigs, at least 14 animals between 2/25/10 and 7/27/10 were found to have injuries to their legs, including 9 guinea pigs that had to be euthanized due to fractures, from being caught in the mesh flooring of the primary enclosures.

Primary enclosures equipped with mesh or wire floors shall be constructed so as to protect the animals' feet and legs from injury.

To be corrected by 8/26/10.

Accompanied on the inspection by the Attending Veterinarian and the Vice President for Technical Services and Business Development. Exit interview performed on site with the Vice President for Technical Services and Business Development and the President.

Prepared By: [signature] VMO
TRACY A THOMPSON, D.V.M. USDA, APHIS, Animal Care
Title: VETERINARY MEDICAL OFFICER Inspector 5044

Date: Aug-26-2010

Received By
(b)(6),(b)(7)c
Title:

Date: Aug-26-2010

AUG 31 2010

2010-11-16 - 000051

Evelyn M Celli/CO/APHIS/USDA
08/10/2010 02:48 PM

To JeffreyB@peta.org
cc
bcc
Subject Re: Fw: Complaint against the Colorado Serum Company by PETA email 1 of 5

August 10, 2010

Jeffrey Brown
JeffreyB@peta.org

Dear Complainant:

Thank you for your emailed letter dated August 10, 2010 concerning conducting regulated activity with guinea pigs. Your concern has been issued number #W10-170 to know the results of our findings, you must send a request, in writing, to our Freedom of Information Act office. Depending on the circumstances of the situation, please allow us enough time (30 to 60 days) to thoroughly investigate your concerns.

FOIA requests can be submitted three ways--

1. Email: foia.officer@aphis.usda.gov
2. Fax: 301-734-5941
3. US Mail: USDA, APHIS, FOIA
   4700 River Road, Unit 50
   Riverdale, MD 20723

Animal Care is the division of the U.S. Department of Agriculture (USDA) that is responsible for the enforcement of the Animal Welfare Act. The Act provides minimum standards for the humane care and use of animals at USDA licensed or registered facilities.

Animal Care inspectors conduct routine unannounced inspections at all USDA licensed and registered facilities to ensure that they are meeting or exceeding these minimum standards. Our inspectors also conduct searches for unlicensed facilities conducting regulated activities. We perform inspections and searches when necessary in response to valid concerns and complaints received from the public to ensure the well-being of the animals and compliance with the law. If violations are found, enforcement action appropriate for the circumstances will be initiated.

Please be assured that we will look into your concerns and take appropriate action if necessary.

Thank you for your interest in the welfare of these animals.

Sincerely,

Robert Gibbens, DVM

Director, Western Region
USDA, APHIS, Animal Care

Connie R Morris   Dear Dr. Gibbens,   08/10/2010 01:21:28 PM

Jeffrey Brown <JeffreyB@peta.org>

08/10/2010 07:21 AM

To <robert.m.gibbens@usda.gov>
cc
Subject Complaint against the Colorado Serum Company by PETA

Dear Dr. Gibbens,

Please see the attached cover letter, complaint and four supporting documents regarding PETA's complaint against the Colorado Serum Company. Because the attachments are larger than your email administrator allows, these items will be sent separately.

If you have any questions or have not received all of the aforementioned documents, please feel free to reach me by email at JeffreyB@peta.org or by telephone at (310) 437-8003.

Sincerely,

Jeffrey Brown
Research Associate
Regulatory Testing Division
People for the Ethical Treatment of Animals
2898 Rowena Avenue, Suite 102
Los Angeles, California 90039
(310) 437-8003

CSC USDA complaint cover letter 8_2010 FINAL.pdf   CSC USDA complaint 8_2010 FINAL.pdf

W10-100
84-R-0002
1086

(b)(6),(b)(7)c

08/10/2010 01:21 PM

To Evelyn M Celli/CO/APHIS/USDA@USDA
cc
bcc
Subject Fw: Complaint against the Colorado Serum Company by PETA email 1 of 5

Jeffrey Brown <JeffreyB@peta.org>

08/10/2010 07:21 AM

To <robert.m.gibbens@usda.gov>
cc
Subject Complaint against the Colorado Serum Company by PETA

Dear Dr. Gibbens,

Please see the attached cover letter, complaint and four supporting documents regarding PETA's complaint against the Colorado Serum Company. Because the attachments are larger than your email administrator allows, these items will be sent separately.

If you have any questions or have not received all of the aforementioned documents, please feel free to reach me by email at JeffreyB@peta.org or by telephone at (310) 437-8003.

Sincerely,

Jeffrey Brown
Research Associate
Regulatory Testing Division
People for the Ethical Treatment of Animals
2898 Rowena Avenue, Suite 102
Los Angeles, California 90039
(310) 437-8003

CSC USDA complaint cover letter 8_2010 FINAL.pdf   CSC USDA complaint 8_2010 FINAL.pdf

PEOPLE FOR THE ETHICAL TREATMENT OF ANIMALS

HEADQUARTERS
501 FRONT STREET
NORFOLK, VA 23510
TEL 757-622-PETA
FAX 757-622-0457

August 9, 2010

Dr. Robert Gibbens
Regional Director
USDA-Western Region
2150 Centre Ave., Bldg. B
Mail Stop #3W11
Fort Collins, CO 80526

Sent via USPS and e-mail to: robert.m.gibbens@usda.gov

Dear Dr. Gibbens:

On behalf of People for the Ethical Treatment of Animals (PETA) and our more than two million members and supporters, I am requesting an investigation into possible violations of the Animal Welfare Act (AWA) related to Institutional Animal Care and Use Committee review and approval of the use of live hamsters by the Colorado Serum Company (CSC).

In 2006, USDA approved the use of a non-animal approach to the batch potency testing of *Leptospira* vaccines in Supplemental Assay Methods (SAMs) 624, 625, 626 and 627, which eliminate the continued use of the animal test. The animal test requires a minimum of 40 hamsters and causes substantial pain and distress in the form of blood poisoning, kidney damage and anorexia. Pain relief is not provided to the animals. We subsequently contacted *Leptospira* vaccine manufacturers to share these approved non-animal methods and to confirm their use in place of the test using hamsters.

Our repeated attempts to contact the Colorado Serum Company have not been answered. Yet a recent review of publicly available APHIS Annual Reports of Research Facilities revealed that the CSC has continued to use the obsolete animal testing methods for its *Leptospira* vaccines despite the availability of the non-animal SAMs since 2006. These reports show that the CSC has not adequately searched for replacements for this painful animal test, instead simply submitting the same language each year justifying its use and recommending that a non-animal test be used when it becomes available.

In light of this information, we ask that you undertake a full investigation into the use of hamsters for *Leptospira* vaccine batch potency testing at the CSC. If non-compliance is found, we urge you to take swift and decisive action that includes citation for violations of the AWA, issuing an Official Warning, levying fines against the CSC, and other appropriate corrective and disciplinary measures. In addition, we urge you investigate the testing for *Leptsospira* vaccine batch potency at other U.S. vaccine manufacturers.

Please feel free to contact me with any questions you may have on this important matter. I can be reached by email at JeffreyB@peta.org or by telephone at (310) 437-8003 and look forward to hearing from you.

Sincerely,

Jeffrey Brown
Research Associate
Regulatory Testing Division

COMPLAINT AGAINST

THE COLORADO SERUM COMPANY

from

People for the Ethical Treatment of Animals

August 9, 2010

People for the Ethical Treatment of Animals (PETA) has uncovered evidence that the Colorado Serum Company (CSC) (84-R-0007) may have committed violations of the Animal Welfare Act related to Institutional Animal Care and Use Committee review and approval of the use of live hamsters for *Leptospira pomona*, *icterohaemmorhagiae*, *canicola*, and *grippotyphosa* bacterin batch potency testing. According to the CSC's documentation and USDA APHIS Annual Reports of Research Facilities obtained by PETA through publicly available APHIS records, between 388 and 533 hamsters have been used each year even though since 2006 APHIS has approved the *in vitro* Supplemental Assay Methods (SAMs) 624, 625, 626 and 627 as a complete replacement for the use of animals for batch potency testing of these *Leptospira* serovar bacterins.

**Annual Report period**	**Hamsters used at the CSC for Category E *Leptospira* vaccine potency tests**
2005	431
2006*	446
2007*	491
2008*	388
2009*	533

* *in vitro* SAMs 624—627 available from USDA

Existing *in vivo* methods and approved *in vitro* replacements

Title 9 of the Code of Federal Regulations (CFR) describes the accepted *in vivo* batch potency assays for *Leptospira* vaccines, serovars *pomona* (9 CFR 113.101), *icterohaemorrhagiae* (9 CFR 113.102), *canicola* (9 CFR 113.103), and *grippotyphosa* (9 CFR 113.104), each of which requires a minimum of forty hamsters. The methods approved for use by these sections are expanded in SAMs 608, 610, 609 and 617, respectively. Over the course of these painful and distressful tests, "clinical signs can be mild to severe," and animals may suffer from "septicemia, nephritis, depression and anorexia."

However, beginning with the 2006 publication of SAMS 624 through 627, *in vitro* batch potency assays were put into use at APHIS and approved for use by all *Leptospira* vaccine manufacturers as replacements for the use of live hamsters for potency testing.

Failure to Search for and Consider Alternatives to the Use of Animals

The attached 2006 through 2009 Research Facility Annual Reports (APHIS Form 7023) for the CSC contain a grossly outdated and inadequate search for alternatives. All Annual Reports submitted for this facility since the 2006 introduction of SAMs 624 through 627 have failed to demonstrate a new search for alternative methods, instead repeating the submission of an obsolete report that was written in 2004.

In this report, the CSC's IACUC concluded that "the best course of action would be to replace the hamster *in vivo* potency test with an *in vitro* potency test that does not require animal testing for potency." However, since alternatives were not approved when this was written in 2004, the CSC IACUC noted that "all biologics companies producing *Leptospira* bacterins will continue to use hamster *in vivo* potency tests" until an *in vitro* replacement test were developed. There is no indication that the CSC IACUC has revised this statement or conducted new searches for alternatives since 2004, though a series of *in vitro* tests has since been approved for use by APHIS. It would also appear that the CSC's "online and textbook search" for alternatives did not include search terms for standard non-animal alternatives such as "ELISA" or "*in vitro*."

If the CSC's IACUC had made "a reasonable and good faith effort" to search for alternatives[1] as required by the AWA, they would have discovered that the objectives of these potency assays can be achieved without the use of animals. The current USDA-approved methodology available in SAMs 624 through 627 suggests immediately available replacements for the use of animals for *Leptospira* vaccine batch potency testing.

Policy 12 of the USDA's *Animal Care Policy Manual* states, "A fundamental goal of the AWA and the accompanying regulations is the minimization of animal pain and distress via the consideration of alternatives[2] and alternative methods.[3]" The CSC should be an expert in the manufacture of *Leptospira* vaccines and, as such, the failure to identify and use existing refined protocols is inexplicable but, regardless, the approval for the use of live hamsters for the aforementioned batch potency tests represents a significant failure to achieve compliance with the AWA on the part of the CSC IACUC.

In light of all the above, it is clear that significant pain and suffering are associated with these procedures, that alternatives to the use of animals are available, and that the CSC's IACUC has failed in its responsibilities under the AWA. Specifically, the CSC IACUC has failed to conduct a thorough search for alternatives, and **given the existence of USDA-approved *in vitro* SAMs, it is simply not possible—as required by the AWA[4]—for an IACUC's required written narrative to demonstrate that alternatives to the use of animals for *Leptospira* vaccine batch potency testing are "not available" and that the use of animals for this purpose "is unavoidable for the conduct of scientifically valuable research.[5]"**

Attachments: 2004--2009 CSC Research Facility Annual Reports

---

[1]Animal and Plant Health Inspection Service, "Alternatives to Painful Procedures."
[2]According to the USDA's Animal Care Resource Guide Policy #12, "Alternatives or alternative methods are generally regarded as those that incorporate some aspect of replacement ... of animal use .... These include methods that use non-animal systems ... to partially or fully replace animals ...."
[3]Animal and Plant Health Inspection Service, "Alternatives to Painful Procedures."
[4]9 C.F.R. §2.31(d)(1); §2.32(c)(5)(ii); §2.36(b)(2)
[5]9 C.F.R. §2.31(e)(4)].

Connie R Morris/CO/APHIS/USDA
08/10/2010 01:21 PM

To Evelyn M Celli/CO/APHIS/USDA@USDA
cc
bcc
Subject Fw: Complaint against the Colorado Serum Company by PETA (2)

Jeffrey Brown <JeffreyB@peta.org>
08/10/2010 07:23 AM

To <robert.m.gibbens@usda.gov>
cc
Subject Complaint against the Colorado Serum Company by PETA (2)

Please see the attached document.

Jeffrey Brown
Research Associate
Regulatory Testing Division
People for the Ethical Treatment of Animals
2898 Rowena Avenue, Suite 102
Los Angeles, California 90039
(310) 437-8003

2006_84-R-0007 records CSC.pdf

This report is required by law (7 USC 2143). Failure to report according to the regulations can result in an order to cease and desist and to be subject to penalties as provided for in Section 2150.

See reverse side for additional information.

Interagency Report Control No 0180-DOA-AN

UNITED STATES DEPARTMENT OF AGRICULTURE
ANIMAL AND PLANT HEALTH INSPECTION SERVICE

ANNUAL REPORT OF RESEARCH FACILITY
(TYPE OR PRINT)

1. REGISTRATION NO. 84-R-0007 CUSTOMER NO. 1086

FORM APPROVED OMB NO. 0579-0036

2. HEADQUARTERS RESEARCH FACILITY *(Name and Address, as registered with USDA, include Zip Code)*
COLORADO SERUM COMPANY
P. O. BOX 16428
DENVER, CO 80216
(303) 295-7527

3. REPORTING FACILITY (List all locations where animals were housed or used in actual research, testing, teaching, or experimentation, or held for these purposes. Attach additional sheets if necessary.)

FACILITY LOCATIONS*(sites)*

REPORT OF ANIMALS USED BY OR UNDER CONTROL OF RESEARCH FACILITY *(Attach additional sheets if necessary or use APHIS FORM 7023A)*

A. Animals Covered By The Animal Welfare Regulations	B. Number of animals being bred, conditioned, or held for use in teaching, testing, experiments, research, or surgery but not yet used for such purposes.	C. Number of animals upon which teaching, research, experiments, or tests were conducted involving no pain, distress, or use of pain-relieving drugs.	D. Number of animals upon which experiments, teaching, research, surgery, or tests were conducted involving accompanying pain or distress to the animals and for which appropriate anesthetic, analgesic, or tranquilizing drugs were used.	E. Number of animals upon which teaching, experiments, research, surgery or tests were conducted involving accompanying pain or distress to the animals and for which the use of appropriate anesthetic, analgesic, or tranquilizing drugs would have adversely affected the procedures, results, or interpretation of the teaching, research, experiments, surgery, or tests. *(An explanation of the procedures producing pain or distress in these animals and the reasons such drugs were not used must be attached to this report)*	F. TOTAL NO. OF ANIMALS (Cols. C + D + E)
4. Dogs	-	-	-	-	-
5. Cats	-	-	-	-	-
6. Guinea Pigs	2	371	328	1482	2181
7. Hamsters	107	404	0	446	850
8. Rabbits	1	0	95	0	95
9. Non-Human Primates	-	-	-	-	-
10. Sheep	-	-	-	-	-
11. Pigs	-	-	-	-	-
12. Other Farm Animals	-	-	-	-	-
13. Other Animals	-	-	-	-	-

ASSURANCE STATEMENTS

1) Professionally acceptable standards governing the care, treatment, and use of animals, including appropriate use of anesthetic, analgesic, and tranquilizing drugs, prior to, during, and following actual research, teaching, testing, surgery, or experimentation were followed by this research facility.
2) Each principal investigator has considered alternatives to painful procedures.
3) This facility is adhering to the standards and regulations under the Act, and it has required that exceptions to the standards and regulations be specified and explained by the principal investigator and approved by the Institutional Animal Care and Use Committee (IACUC). A summary of all the exceptions is attached to this annual report. In addition to identifying the IACUC-approved exceptions, this summary includes a brief explanation of the exceptions, as well as the species and number of animals affected.
4) The attending veterinarian for this research facility has appropriate authority to ensure the provision of adequate veterinary care and to oversee the adequacy of other aspects of animal care and use.

CERTIFICATION BY HEADQUARTERS RESEARCH FACILITY OFFICIAL
(Chief Executive Officer or Legally Responsible Institutional official)
I certify that the above is true, correct, and complete (7 U.S.C. Section 2143)

SIGN[illegible] [illegible]FICIAL	NAME & TITLE OF C.E.O. OR INSTITUTIONAL OFFICIAL *(Type or Print)*	DATE SIGNED
(b)(6),(b)(7)(c)	(b)(6),(b)(7)(c)	10-19-06

APHI[illegible] [illegible]18-23 (Oct 88), which is ob[illegible]
(AUG 91)

PART 1 - HEADQUARTERS

OCT 2 3 2006

2010-11-16 - 000060

ATTACHMENT NO. 1

Colorado Serum Company
License No. 84-R-0007

For FY 2005-2006 Annual Report of Registered Research Facility

Each animal in each referenced test was employed in testing a Licensed Veterinary Biological product as required by Federal Regulations as codified in Title 9CFR. Humane euthanasia of animals on 9CFR required tests is provided for in 9CFR 117.4(e) - Animals at Licensed Establishments - Test Animals (filed August 19, 1995). This is included as standard testing protocol. While limiting the duration of the pain and distress, it does not fulfill the description of use as described treatment in Section D of APHIS Form 7023, Page 1. Therefore, we feel obligated to continue to include these test animals in Section E.

Attending veterinarians and related employees have been informed and Colorado Serum Company is complying with this provision.

Explanation of usage of animals listed in Column E.

Line Item 6. Guinea Pigs

1482 guinea pigs were tested causing pain, for which no anesthetic, analgesics, or tranquilizers could be used. A detailed breakdown of guinea pigs by test reference is provided.

9CFR 113.451(b) Tetanus Antitoxin potency testing - 1342 guinea pigs

Humane endpoints were addressed for all animals on this test, thus reducing the duration of pain and suffering.

9CFR 113.106(c) Clostridium Chauvoei Bacterin potency testing - 140 guinea pigs

Humane endpoints were addressed for all animals on this test, thus reducing the duration of pain and suffering.

OCT 2 3 2006

Colorado Serum Company
License No. 84-R-0007

Line Item 7 Hamsters

446 hamsters were tested causing pain, for which no anesthetics, analgesics, or tranquilizers could be used. A detailed breakdown of hamsters by test referenced is provided.

9CFR 113.101(c) Leptospira Pomona Bacterin potency testing - 111 hamsters

The 111 hamsters included required controls for potency tests [9CFR 113.101(c)(2)], plus necessary hamsters required to establish the validity of the challenge dose (10 to 10,000 hamster LD_{50}) by titration. 9CFR 113.101 (c)(3).

For all test hamsters, Colorado Serum Company specifically has applied provisions of 9CFR 117.4(e) wherein ill animals were humanely euthanized upon reaching a point where death was expected to occur.

9CFR 113.102(c) Leptospira Icterohaemorrhagiae Bacterin potency testing - 99 hamsters

The 99 hamsters included required controls for potency tests [9CFR 113.102(c)(2)], plus necessary hamsters required to establish the validity of the challenge dose (10 to 10,000 hamster LD_{50}) by titration. 9CFR 113.102(c)(3).

For all test hamsters, Colorado Serum Company specifically has applied provisions of 9CFR 117.4(e) wherein ill animals were humanely euthanized upon reaching a point where death was expected to occur.

9CFR 113.103(c) Leptospira Canicola Bacterin potency testing - 106 hamsters

The 106 hamsters included required controls for potency tests [9CFR 113.103(c)(2)], plus necessary hamsters required to establish the validity of the challenge dose (10 to 10,000 hamster LD_{50}) by titration. (CFR 113.103(c)(3).

OCT 2 3 2006

Colorado Serum Company
License No. 84-R-0007

Line Item 7	9CFR 113.103(c)	Continued
		For all test hamsters, Colorado Serum Company specifically has applied provisions of 9CFR 117.4(e) wherein ill animals were humanely euthanized upon reaching a point where death was expected to occur.
	9CFR 113.104(c)	Leptospira Grippotyphosa Bacterin potency testing - 130 hamsters
		The 130 hamsters included required controls for potency tests [(9CFR 113.104(c)(2)], plus necessary hamsters required to establish the validity of the challenge dose (10 to 10,000 hamster LD_{50}) by titration. 9CFR 113.104 (c)(3).
		For all test hamsters, Colorado Serum Company specifically has applied provisions of 9CFR 117.4(e) wherein ill animals were humanely euthanized upon reaching a point where death was expected to occur.

(b)(6),(b)(7)(c) ____________ 10-19-06
Date

OCT 2 3 2006

COLORADO
SERUM COMPANY

PHONE: (303) 295-7527
FAX: (303) 295-1923 (b)(2)High, (b)(7)(F) P.O. Box 16428 Denver, Colorado 80216-0428

July 22, 2004

Subject: Investigation by Colorado Serum Company's Institutional Animal Care and Use Committee into hamster analgesia and its possible usage in hamsters during the *Leptospira* challenge portion of *Leptospira* potency tests at Colorado Serum Company.

From: (b)(6),(b)(7)(c)

To: Colorado Serum Company Institutional Animal Care and Use Committee and Mr. (b)(6),(b)(7)(c) Colorado Serum Company.

Background: Use of hamsters for potency tests when developing Leptospira organisms for bacterin production has been the requirement by USDA (9-CFR, 113.101 – 113.105) for many years. Ten vaccinates and 10 or more controls are challenged intraperitoneally with a suspension of virulent Leptospira organisms. This applies to potency tests for *Leptospira pomona, Leptospira icterohaemorrhagiae, Leptospira canicola* and *Leptospira grippotyphosa* fractions. If 8 or more controls die from leptospirosis during a 14 day post challenge observation period then the test is considered valid. The degree of pain and suffering to the control hamsters is of concern for animal welfare reasons and an alternative in-vitro test that has reproducible and comparable results to the in-vivo potency test would be a much better alternative replacement test. Until that happens all biologic companies producing *Leptospira* bacterins will continue to use hamster in-vivo potency tests.

The degree of pain and suffering the control hamsters endure during this 14 day observation period is a subjective evaluation given the fact that hamsters cannot communicate with humans. Based on the disease syndrome of Leptospirosis in our domestic species the clinical signs can be mild to severe. In most animal species the disease causes septicemia, nephritis, depression and anorexia. It is safe to assume that hamsters suffer from many of these symptoms as well, and it is most likely not a chronic pain as much as it is a general discomfort or sick feeling that they are experiencing. Parameters of hamster behavior used to measure discomfort or pain would include exploration, grooming and posture as well as food and water consumption and fur quality.

Analgesic use in hamsters: An on-line and textbook search conducted by Colorado Serum Company's IACUC (b)(6),(b)(7)(c) revealed very few drug options for analgesia in hamsters (see attached documents). Buprenorphine was the most common analgesic for hamsters cited. The labeled route is by subcutaneous injection and the

frequency is every 8 hours. In one published paper (see attached) buprenorphine was used in the syngeneic murine tumor model to investigate whether the pain or discomfort in mice with these tumors could be reduced with this analgesic via an oral route. In this study they found no significant difference in pain or discomfort between the buprenorphine treated group and the control group based on their scoring system. Even though this experiment was done with mice it would be plausible to compare the discomfort or pain from multiple tumors to that which would be experienced by hamsters during a systemic leptospirosis infection. Also the process of handling these hamsters and giving them injections 2 – 3 times daily would itself be stressful and painful. There would be a significant zoonotic concern for the person involved giving the injections to multiple infected hamsters from the urine as well as an increased chance of being bitten considering you are giving injections to sick and uncomfortable hamsters 2 to 3 times daily for up to 14 days. Treating the symptoms without treating the source of the disease with antibiotics (which can't be done for obvious reasons) is also of questionable value. There would also be concern as to what effect this analgesic would have on the outcome of the potency test. All drugs are processed and eliminated by the liver and kidneys and since these are two target organs of *Leptospira* organisms, toxic levels of buprenorphine could buildup in a *Leptospira* infected hamster and could alter the time of death or even contribute to a death that might not otherwise happen - thus affecting the outcome of the potency test and possibly resulting in a falsely valid test. Other drugs mentioned are injectable also and do not offer anything unique or superior to buprenorphine.

The Cornell website detailed an oral preparation of buprenorphine in jello for use in mice (see attached). Whether this would work in hamsters is not known. One concern is the fact that anorexia is a very common symptom with Leptospirosis and depending on the degree of anorexia and the fact that these hamsters are usually housed 5 per cage, some hamsters may be getting their medication and some may not - due to anorexia or competition. This could also cause some hamsters to overdose on buprenorphine if they all aren't eating the same amount of jello.

Conclusion:
Colorado Serum Company's IACUC has determined that no analgesia we have found can realistically be expected to relieve an infected hamster from the symptoms of Leptospirosis especially without concurrent use of antibiotics to fight the etiologic agent (*Leptospira*) since this would defeat the purpose of the potency study. Buprenorphine works well with acute, sharp post-surgical type pain and would be of very questionable value in hamsters suffering from discomfort associated with a systemic infection. The best course of action in our opinion would be to replace the hamster in-vivo potency test with an in-vitro test that does not require animal testing for potency.

(b)(6),(b)(7)(c)

COLORADO SERUM COMPANY

PHONE: (303) 295-7527
FAX: (303) 295-1925

(b)(2)High, (b)(7)(F) P.O. Box 16428 Denver, Colorado 80216-0428

November 11, 2004

Subject: Investigation by Colorado Serum Company's Institutional Animal Care and Use Committee into guinea pig analgesia and its possible usage in guinea pigs during the *Clostridium tetani* antitoxin potency tests done at Colorado Serum Company.

From: (b)(6),(b)(7)(c)
Veterin[illegible].

To: Colorado Serum Company's Institutional Animal Care and Use Committee and Mr. (b)(6),(b)(7)(c) Colorado Serum Company.

Background: Use of guinea pigs for potency tests when developing Tetanus Antitoxin has been the requirement by USDA (9-CFR, 113.451) for many years. The potency test is essentially an in-vivo neutralization test that requires two guinea pigs each for controls and for each dilution. The two controls are injected subcutaneously with a 3 ml dose of the Standard Toxin-Antitoxin mixture. Injections shall be made in the same order that toxin is added to the dilutions of antitoxins. These shall be observed parallel with the titration of one or more unknown antitoxins. Two test guinea pigs will be used for each dilution of the unknown antitoxin (also a 3 ml dose, subcutaneously). Controls are observed until they are down and are unable to rise or stand under their own power. At this time they are humanely euthanized and the time of death is recorded in hours. For a satisfactory test, the controls must reach this point with clinical signs of tetanus within 24 hours of each other and within an overall time of 60 – 120 hours. The clinical signs to be observed are increased muscle tonus, curvature of the spine, asymmetry of the body outline when the resting animal is viewed from above, generalized spastic paralysis, particularly of the extensor muscles, inability to rise from a smooth surface when the animal is placed on its side, or any combination of these signs. If the control guinea pigs do not respond in this manner the entire test shall be repeated. Potency of an unknown antitoxin is determined by finding the mixture which will protect the test animal the same as the Standard Toxin-Antitoxin mixture. Test animals dying sooner than the controls indicate the unit value selected in that dilution was not present, whereas those living longer indicate a greater unit value.

Analgesia in Guinea Pigs: An on-line and textbook search conducted by Colorado Serum Company's IACUC (b)(6),(b)(7)(c) revealed a variety of drugs used in guinea pigs after surgical procedures. The Duke University IACUC website had a very thorough

OC[illegible] 2 3 200[illegible]

guideline for analgesia in all rodents and a variety of drugs and routes of administration based on the degree of pain and discomfort of a given procedure (see attached). Injectable analgesic options for guinea pigs include Codeine, Nalbuphine or Morphine given every 4 hours or Buprenorphine every 12 hours beginning just before symptoms are estimated to begin. Oral medications include Aspirin or Phenylbutazone added to the drinking water once daily, however once guinea pigs begin to show signs of tetanus it can be expected that their water consumption will diminish greatly and that once they are prostrate they will physically be unable to stand and drink. At the later stages of tetanus they are unable to swallow. Euthanasia hopefully is done before the late stages of disease but unfortunately death is sometimes the outcome with the test pigs before the study investigator can officially call the test over, if they have a lower level of antibody protection when compared to the Standard Controls.

Conclusion: There is obviously a direct conflict between animal welfare issues (pain and suffering) and this neutralization potency test that is required by USDA-APHIS-CVB for each new serial of tetanus antitoxin. The dilemma is that the test relies solely on symptoms of tetanus in relation to the Standard Controls as described above, in essence pain and suffering are components of the measured parameters of the test and giving any drugs to alter or help alleviate the symptoms will affect the results of the test. Currently the best welfare that can be provided is humane euthanasia once the symptoms have reached the point that the study investigator can interpret the test and give the staff veterinarian the o.k. to intervene and euthanize.

Due to this direct conflict, Colorado Serum Company's Institutional Animal Care and Use Committee has determined that there is no practical way to intervene with pain medications during the Tetanus Antitoxin potency neutralization test without altering the animal symptoms and thus altering the interpretation of the test. The only solution to this is to replace the guinea pig test with an in-vitro test (which Colorado Serum Company would be greatly in favor of). Currently there is a Competitive Elisa Test for Tetanus Antitoxin that has been tried but unfortunately it has not shown consistent, comparable results when run in parallel with the guinea pig neutralization test.

(b)(6),(b)(7)(c)

November 12, 2004

COLORADO SERUM COMPANY
Established 1923
COLORADO Veterinary Products
Phone: (303) 295-7527
Fax: (303) 295-1923
(b)(2)High, (b)(7)(F) P.O. Box 16428 - Denver, Colorado 80216-0428

October 19, 2006

Dr. Robert M. Gibbens, DVM
Director, Western Region Animal Care
USDA-APHIS-Animal Care
2150 Centre Avenue
Building B Mail Stop #36011
Ft. Collins, CO 80526

Dear Dr. Gibbens:

Enclosed please find our Research Facility Annual Report (APHIS Form 7023).

Supplementary documents are included which were enclosed with the 2005 Annual Report.

1. "Investigation into Guinea Pig Analgesia.......... ", dated November 11, 2004.
2. "Investigation by Colorado Serum Company's IACUC into Hamster Analgesia...... ", dated July 22, 2004.

In each case we have found no new information which would allow a change in our approach to these issues.

We have also enclosed a description of "Symptoms in Control Guinea Pigs after Challenge with *Clostridium chauvoei*", dated December 19, 2004.

Throughout this year, provisions in 9CFR 117.4(e) were applied to all animals in tests conducted according to 9CFR 113.451(b), 9CFR 113.106(c), 9CFR 113.101(c), 9CFR 113.102 (c), 9CFR 113.103(c), and 9CFR 113.104(c).

In each test, guinea pigs or hamsters that showed clinical signs of illness due to the test were humanely destroyed if the illness progressed to the point when death was certain to occur without therapeutic intervention.

Regarding another matter, we request that you continue to use our P.O. Box 16428 on future inspection reports instead of our street address.

OCT 23 2006

Gibbens
Page 2

Please contact us if there are any questions.

Sincerely,

JNH:al
Enclosure

OCT [illegible]

December 29, 2004

(b)(6),(b)(7)(c)

Subject: **Symptoms in control guinea pigs after challenge with *Clostridium chauvoei* and *hemolyticum* during *Cl. chauvoei* and *hemolyticum* bacterin in vivo potency tests.**

These are symptoms that I have personally observed during this potency test. I will also include some more detailed textbook description of these symptoms to use if you like.

This potency tests for both of these fractions involves a 3 day observation after IM challenge with ~100 LD_{50} of live bacteria (*Cl. chauvoei or hemolyticum*). Within 24 to 48 hours the first symptoms of disease begin to appear. Lethargy, anorexia, stiffness with reluctance to move, sero-purulent discharge from the festering injection site, and vocalization (associated with pain) when handled are frequent symptoms most commonly seen. The swelling at the injection site is hot and painful at first and eventually becomes cold and necrotic as the infection becomes systemic. A high fever is also associated with these Clostridial diseases. Once symptoms are obvious the disease progresses quickly and death can happen rapidly. Once obvious symptoms are observed, veterinary intervention and humane euthanasia is implemented whenever possible.

[illegible]
