# Supplementary material for: Bridging the Gap Between Validation and Implementation of Non-Animal Veterinary Vaccine Potency Testing Methods
Source: Animals (Basel). 2011 Nov 29;1(4):414–32. doi: 10.3390/ani1040414 (PMC4513470; doi:10.3390/ani1040414)
Supplement: Supplementary File 1 [file animals-01-00414-s001.zip › supplementary materials/28 CVB clostridials.pdf]

USDA

United States Department of Agriculture

Animal and Plant Health Inspection Service

Veterinary Services

Center for Veterinary Biologics

1920 Dayton Avenue
P.O. Box 844
Ames, IA 50010

(515) 337-6100

COPY

December 4, 2009

Mr. Jeffrey Brown
PETA
501 Front Street
Norfolk, VA 23510

Dear Mr. Brown:

This letter is in response to your October 30, 2009, inquiry to me and September, 2009, e-mail conversation with Dr. Geetha Srinivas regarding clostridial testing reagents and protocols. Thank you for your continued interest in our program. As with the issue of testing *Erysipelas rhusiopathiae* biologics, we welcome the opportunity to describe our work in the development of alternatives to animal usage in the potency testing of veterinary biologics.

You are correct that monoclonal antibodies against the alpha and beta toxins of *Clostridium perfringens*, *C. sordellii* lethal toxin, and *C. chauvoei* flagellar toxin are available as reagents from the Center for Veterinary Biologics (CVB). CVB Notice 02-09 discusses obtaining monoclonal antibodies from CVB, but currently these monoclonal antibodies would be obtained via the process outlined in Veterinary Services Memorandum 800.97 rather than the American Type Culture Collection. As you can see from this reagent list, we are laying the groundwork for *in vitro* testing for the clostridial antigens as well. There has been some preliminary work in utilizing these reagents for *in vitro* testing, as the CVB is committed to the reduction of animal usage as a part of our program. Of course, as you aptly point out, harmonization is part of this process. Tests we develop need to be applicable to all the products on the market and correlated with efficacy and past testing methods. Coordinated phase-in of test methods is a significant step in the introduction of reagents.

In the case of *Clostridium chauvoei* Flagella-specific Monoclonal Antibody, it is listed, in Veterinary Services Memorandum 800.97, as a reagent that can be obtained from CVB. The accompanying reagent data sheet indicates that it is for use in a capture enzyme-linked immunosorbent assay as described in Draft Supplemental Assay Method 220 for potency testing of *C. chauvoei* bacterins.

In terms of test methods used by Europe, the United States participates in VICH, a trilateral program aimed at harmonizing technical requirements for veterinary product registration for the European Union, Japan, and the United States. The full title of the program is the International Cooperation on Harmonization of Technical Requirements for Registration of Veterinary Medicinal Products. VICH was officially launched in April 1996. The CVB is actively working, through VICH, to harmonize our testing so there is a reduction of all testing for products with international markets, including testing using animals.

In regard to Supplemental Assay Method (SAM) 213, I am unaware of any confusion regarding this SAM. It is currently being updated. CVB has an active Quality Management system under which our documents are reviewed and updated on a regular basis. I have requested that a copy of the current version, that is undergoing updating, be sent to you under separate cover. Under our Quality Management system, a SAM cannot be obsoleted as long as a licensed product is still being manufactured and tested by this method.

If you have any questions regarding this letter, please contact me at your convenience.

Sincerely,

Richard E. Hill, Jr.
Director
Center for Veterinary Biologics
