# Supplementary material for: Bridging the Gap Between Validation and Implementation of Non-Animal Veterinary Vaccine Potency Testing Methods
Source: Animals (Basel). 2011 Nov 29;1(4):414–32. doi: 10.3390/ani1040414 (PMC4513470; doi:10.3390/ani1040414)
Supplement: Supplementary File 1 [file animals-01-00414-s001.zip › supplementary materials/29 CVB clostridials and ascites.pdf]

USDA

United States Department of Agriculture

Animal and Plant Health Inspection Service

Veterinary Services

Center for Veterinary Biologics

1920 Dayton Avenue
P.O. Box 844
Ames, IA 50010

(515) 337-6100

RECEIVED
JUN 23 2010

[illegible]ne 17, 2010

Mr. Jeffrey Brown
People for the Ethical Treatment of Animals
501 Front Street
Norfolk, VA 23510

Dear Mr. Brown:

This is in response to your March 29, 2010, letter regarding clostridial vaccine testing, monoclonal antibody production, and obtaining monoclonal antibodies from the Center for Veterinary Biologics (CVB).

As we have indicated previously, one of the CVB's goals is to reduce, refine, and replace animal testing. Our program needs to do this while ensuring our veterinary biologics are of the highest quality. With these goals in mind, we encourage open communication, and collaborate with the scientific community and our manufacturers, as new products and standards for products are developed. We strongly believe such communications and exchanges of ideas can lead to a reduction in animal use. It does also lead to "draft" documents that may be converted to other types of documents at a later date.

In some cases, an *in vitro* test may work for some manufacturers' products and not others. In such cases, we will use our regulatory flexibility to allow differing methods that still demonstrate compliance with our regulatory standards. This is the reason that you may find some documents listed as additions, instead of replacements, once a test has been fully validated for product testing. You may also see draft Supplemental Assay Methods (SAMs) converted to protocols. As I pointed out to you in my December 4, 2009, letter, the tests we develop need to be applicable to all products, correlated with efficacy, and correlated to past testing methods.

Such is the case with SAM 200 and Draft SAM 220. In regard to your request for a copy of the latter document, we have an active quality management program and are updating much of our documentation. SAM 220 is currently undergoing review, revision, and conversion to a protocol. As SAM 220 and other documents are finalized, we will post copies to our website. While it is our intent to post protocols to our website, this is currently not the procedure.

In the meantime, I hope that the following summary will be helpful: The document that you requested describes a potency test for Clostridium Chauvoei Bacterins that employs a capture enzyme-linked immunosorbent assay to measure the flagellar protein of *C. chauvoei*. Relative potency is determined by comparing the flagellar protein content of the test serial to that of a non-expired, suitably qualified reference bacterin. When the protocol is finalized, we will send your group a copy.

Although the CVB is primarily a regulatory program, rather than a research program, we have made steady advances in developing reagents and *in vitro* test methods to replace those you cited for other clostridial products. Although it is difficult to provide a timeline for the research and implementation of such methods, I assure you that the CVB is devoting resources toward replacing these tests.

In regard to monoclonal antibody production, the advances in the production methods you cited (dialysis cartridge roller and hollow fiber systems) are currently in use at the CVB. This has allowed a significant reduction in animal usage for that purpose. The CVB has made capital equipment acquisitions for the purchase of several types of *in vitro* production systems and for equipment to process the resulting reagent. The majority of our hybridoma cell lines can make sufficient antibody in the various bioreactor systems, and we are evaluating and qualifying these reagents.

The above efforts notwithstanding, the CVB has prepared reagents for use in testing of vaccines for decades. For this reason, some of our reagents date to a time when ascites production was the only suitable, consistent production method. Due to the stability and concentration of this type of reagent, we have stockpiles of these reagents. We feel depleting stocks of these valuable products—for which animals have already been used—through distribution is preferable to destruction. When the current lot is depleted, the replacement lots will most likely be produced by the bioreactor technologies that the CVB has shown to be successful.

In order to supply our stakeholders with quality reagents that are consistent among lots, new lots must be qualified against previous, acceptable lots. Preparation of some monoclonal antibodies may not transition easily to bioreactors, so these antibodies may continue to be prepared via ascites production for a time. Therefore, you will continue to see ascites reagents listed in Veterinary Services Memorandum 800.97. In addition, CVB-prepared monoclonal antibodies are currently listed in, and can be obtained via the request process outlined in, this memorandum. The reference to CVB Notice 02-09 in my December 2009 letter was an error; I regret any confusion this may have caused.

Finally, I would like to point out that the availability of monoclonal antibodies, prepared in mice, has allowed the development and implementation of *in vitro* potency tests, which has dramatically reduced the use of host animals. If the monoclonal antibodies were not available, we would need to return to testing in the host animal in order to provide quality vaccines.

If you have further questions about these issues, please contact me at your convenience.

Sincerely,

Richard E. Hill, Jr.
Director
Center for Veterinary Biologics
