# Supplementary material for: Bridging the Gap Between Validation and Implementation of Non-Animal Veterinary Vaccine Potency Testing Methods
Source: Animals (Basel). 2011 Nov 29;1(4):414–32. doi: 10.3390/ani1040414 (PMC4513470; doi:10.3390/ani1040414)
Supplement: Supplementary File 1 [file animals-01-00414-s001.zip › supplementary materials/31 CVB Newcastle disease.pdf]

USDA

February 8, 2011

United States Department of Agriculture

Animal and Plant Health Inspection Service

Veterinary Services

Center for Veterinary Biologics

1920 Dayton Avenue
P.O. Box 844
Ames, IA 50010

(515) 337-6100

RECEIVED
FEB 15 2011

Mr. Jeffrey Brown
People for the Ethical Treatment of Animals
501 Front Street
Norfolk, VA 23510

Dear Mr. Brown:

This letter is in response to your November 23, 2010, inquiries regarding codified animal vaccinate-challenge tests associated with leptospira bacterins and Newcastle Disease Vaccines.

Regarding leptospira bacterins, you inquired about Master Seed (MS) lot approval being a prerequiste for the use of Supplemental Assay Methods (SAMs) 624, 625, 626, and 627. Seed lot approval is part of the licensure process.

For each microorganism (Master Seed) used in the production of biological products, the manufacturer is required to submit a report describing testing performed to evaluate the purity (freedom from extraneous agents), identity, and safety of the seed. If the report is acceptable, the Center for Veterinary Biologics (CVB) authorizes the transfer of the MS for confirmatory testing at our lab. If our laboratory verifies the manufacturer's results, the MS may be transferred to their production facilities for use in the manufacture of biological products.

Before product prepared from a MS is eligible for sale, efficacy of the product from the seed(s) is determined. The efficacy is then correlated to a potency test which is repeated for each serial of product released to the market. The potency test is used to ensure efficacy of each serial. In the case of leptospira bacterins, the potency test is typically the codified hamster test or the SAM ELISA test.

Therefore, approval of a master seed lot(s) and the performance of a satisfactory potency test would be required for any veterinary biological product offered for sale in the United States. If you refer to Veterinary Services Memorandum 800.102, you will find the guidelines to utilize the ELISA tests rather than the codified hamster test. A copy of the memorandum is enclosed, for your information.

In regard to inactivated Newcastle Disease Vaccines, you inquired about the use of *in vitro* test methods as it relates to comments from a presentation given by Dr. Geetha Srinivas at the Interagency Coordination Committee on the Validation of Alternative Methods (ICCVAM), International Workshop on Alternative Methods to Reduce, Refine, and Replace the Use of Animals in Vaccine Potency and Safety Testing: Sate of the Science and Future Directions, in September 2010. The codified test for this product is a chicken vaccination-challenge.

There was context associated with Dr. Srinivas' public remarks that is not present in your summary. Dr. Srinivas was explaining that correlation approaches between efficacy and potency can be varied. For poultry vaccines in which potency and efficacy testing is routinely conducted in a host-like animal (i.e. SPF chickens), the correlate between potency

might be more straightforward in the sense that it is closer to an animal of interest (i.e. broiler or layer chickens). This is opposed to cattle or swine products using a laboratory animal model of a mouse, for example.

A correlation between chicken serology and protection in the case of licensed Newcastle Disease Vaccines has not been established. In the United States, other *in vitro* tests have not had success, either. Additionally, husbandry practices, nature of vaccine strains, and nature of challenge strains may differ between the United States and other countries, and all this must be factored into changes in testing or the interpretation of test methods. Therefore, while the CVB would welcome a new *in vitro* test for these types of vaccines, a suitable replacement has not been identified.

Please be assured that manufacturers that approach the CVB with *in vitro* potency tests to replace *in vivo* potency tests may receive exemptions, provided the data support a correlation of product efficacy and serial potency.

Thank you again for your continued interest in our program and our effort to reduce, refine, and replace animal use in the testing of licensed veterinary biologics.

Sincerely,

Richard E. Hill, Jr.
Director

Enclosure:
VSM 800.102

May 23, 2002

VETERINARY SERVICES MEMORANDUM NO. 800.102

Subject: Exemption from Leptospira Bacterin Testing Under 9 CFR 113.101(c), 113.102(c), 113.103(c), and 113.104(c)

To: Biologics Licensees, Permittees, and Applicants
Directors, Center for Veterinary Biologics

I. PURPOSE

This memorandum provides guidance for obtaining an exemption to the requirement for testing leptospiral bacterins for potency in hamsters.

II. BACKGROUND

APHIS has published Standard Requirement (SR) potency tests for leptospiral bacterins in 9 CFR 113.101(c), 113.102(c), 113.103(c), and 113.104(c). These regulations require that such bacterins be tested for potency using a vaccination-challenge test conducted in hamsters. However, the regulations under 9 CFR 113.4(a) provide for the granting of an exemption to this requirement if the replacement test is at least equivalent to the SR test, and the test methods and procedures are described in the filed Outline of Production for the product.

In keeping with its commitment to replace animal tests with in vitro tests, APHIS' Center for Veterinary Biologics-Laboratory, in collaboration with veterinary biologics manufacturers, developed and validated monoclonal antibody based antigen capture enzyme-linked immunoassays (ELISAs) for potency testing leptospiral bacterins containing *Leptospira interrogans* serovars *pomona, icterohaemorrhagiae, canicola,* and *grippotyphosa*.

At this time, the Center for Veterinary Biologics (CVB) is reviewing the SRs for leptospiral bacterins. While the review is not yet complete, we anticipate that a future proposed amendment to these requirements will include replacing hamster potency tests with ELISA tests. In anticipation of that proposal, we encourage firms to begin using the ELISA tests developed by CVB-L for releasing serials of leptospira bacterins.

This memorandum clarifies CVB policy concerning the granting of an exemption allowing ELISAs developed by the CVB-L to be used for potency testing leptospiral bacterins containing *Leptospira interrogans* serovars *pomona, icterohaemorrhagiae, canicola,* and *grippotyphosa* in place of the hamster tests specified in the regulations.

Firms that are not interested in obtaining an exemption may continue to perform the hamster potency test pending revision of the SRs.

III. POLICY

To obtain an exemption to use the ELISAs developed by CVB-L in place of the SR test(s) for serial release testing of Leptospira Bacterin:

A. Request an Exemption to the SR test

Under 9 CFR 113.4, the licensee or permittee must request an exemption to the applicable SR test for Leptospira Bacterin prescribed in the regulations in sections 113.101, 113.102, 113.103, and 113.104.

B. Submit Protocols

Simultaneous with the firms request for an exemption to use the CVB-L ELISAs, protocols for qualifying a reference bacterin(s) for use with each assay, should be submitted to CVB for review and comment. Protocols should address the following:

1. *Procedures for qualifying reference bacterins in host animals* - It is recommended that reference bacterins be monovalent, contain the minimum antigenic mass specified in the filed Outline of Production, and qualified using a vaccination-challenge study in each species of host animals for which the product is recommended. For not less than 4 weeks after challenge, animals used in the vaccination-challenge study should be monitored for:

a. Clinical signs including elevated temperature, behavioral changes, changes in appetite or water consumption, dehydration, icterus, oliguria, general condition, and

b. Complete blood count, circulating antibodies to the relevant serovars, urinalysis (pH, bloody urine, protein, urobilinogen, and microscopic examination of sediment), and blood and urine cultures for the presence or absence of leptospires.

2. *Procedures for correlating the host animal protective dose to its protective endpoint in hamsters* - This should be done by performing limiting dilution studies using the applicable codified test in 9 CFR sections 113.101-104.

C. Specify the CVB-L ELISA in the Outline of Production

The CVB-L ELISA procedure(s) for *Leptospira pomona, Leptospira icterohaemorrhagiae, Leptospira canicola,* or *Leptospira grippotyphosa*, as applicable, must be described in the Outline of Production.

D. Document Exemptions

If CVB grants an exemption from the Standard Requirement test, Part V of the Outline of Production must indicate the date the exemption was granted.

/s/ Andrea M. Morgan for

W. Ron DeHaven
Deputy Administrator
Veterinary Services

APHIS:VS:CVB-LPD:KWRuby:APMorgan:hm:734-8245:5-10-02:I:\...\OS\01-013102
ECMS #0205-ZFZJHA
