# Supplementary material for: Bridging the Gap Between Validation and Implementation of Non-Animal Veterinary Vaccine Potency Testing Methods
Source: Animals (Basel). 2011 Nov 29;1(4):414–32. doi: 10.3390/ani1040414 (PMC4513470; doi:10.3390/ani1040414)
Supplement: Supplementary File 1 [file animals-01-00414-s001.zip › supplementary materials/35 VMD TABST.pdf]

VETERINARY MEDICINES DIRECTORATE

VMD

ASSURING THE SAFETY, QUALITY AND EFFICACY OF VETERINARY MEDICINES

Alistair Currie
Senior Research and Campaigns Co-ordinator
PETA – Europe
PO Box 36668
London

Date: 16th October 2008

Dear Alistair,

Thank you for your enquiry concerning information related to batch safety testing of vaccines.

The VMD is the competent authority responsible for the authorisation and regulation of veterinary medicines within the UK, which includes vaccines which have received a marketing authorisation either nationally or through a European procedure and the release of batches of these vaccines. Products authorised nationally are regulated by the national competent authority. For products authorised through a European procedure, all member states concerned in the procedure are involved in regulating the product.

The VMD does not undertake animal testing and has been instrumental in Europe to retain the possibility to release products under an Official Batch Protocol Review (OBPR), which means that with the exception of a small number of high risk products (e.g. rabies vaccine, which are subject to special measures in the interests of animal and public health) batch release in the UK uses the manufacturers testing results and there is no re-testing.

As you are probably aware, since 2002 the European Pharmacopoeia (Ph.Eur.) (Version 4.6) General Monograph on Vaccines for Veterinary Use (0062) has included the possibility to remove the batch safety test provided certain criteria are met. The Committee for Veterinary Medicinal Products (CVMP) is a scientific body under the EMEA, which is composed of representatives from all member states, which has supported the Ph.Eur. position through the preparation of specific guidance (EMEA/CVMP/965/03/FINAL). The safety test performed by the manufacturer can be waived by the competent authority in the interests of animal welfare when a sufficient number of consecutive production batches have been produced and found to comply with the test, thus demonstrating consistency of the manufacturing process. In most cases this can be achieved through the production of 10 consecutive batches that are shown to be safe in the batch safety test.

The holders of marketing authorisations are responsible for applying to vary the terms of the authorisation, which are dealt with through a variations procedure for which there is a fee that is proportionate to the amount of assessment required to review the information. The VMD has highlighted the possibility to remove the batch safety test to manufacturers through its quarterly newsletter and as part of presentations to industry, and encourages such applications whenever possible. It is our experience that manufacturers see the benefits of the removal of the batch safety test for both ethical and financial reasons. It is also our experience that member states involved in European authorisations are also supportive of such applications. It should be noted that the rate at which applications are made will be dependent upon market usage and the time taken to accumulate data from 10 batches. The UK (represented by the VMD) have been involved in a number of applications to remove the batch safety test for products, all of which have been approved. In most cases this has meant that those products are no longer subject to any animal tests performed for batch release purposes.

Concerning the influence of non-EU requirements on this issue, since this falls outside our scope we do not have any information and therefore cannot offer an opinion.

I hope this information is helpful.

Yours sincerely

Steve Spencer PhD
Immunologicals Assessor
Direct dial 01932 338418
email:*s.spencer@vmd.defra.gsi.gov.uk*
