# Supplementary material for: Bridging the Gap Between Validation and Implementation of Non-Animal Veterinary Vaccine Potency Testing Methods
Source: Animals (Basel). 2011 Nov 29;1(4):414–32. doi: 10.3390/ani1040414 (PMC4513470; doi:10.3390/ani1040414)
Supplement: Supplementary File 1 [file animals-01-00414-s001.zip › supplementary materials/36 UK DfEFR TABST.pdf]

Nobel House
17 Smith Square
London SW1P 3JR

Telephone 08459 335577
Email helpline@defra.gsi.gov.uk
Website www.defra.gov.uk

defra
Department for Environment Food and Rural Affairs

Alistair Currie
Senior Research and Campaign Coordinator
PeTA
PO Box 36668
London
SE1 1WA

Our Ref: PO125985/LD

23rd April 2009

From Rt Hon Jane Kennedy MP
Minister of State for Farming and the Environment

Dear Mr Currie

Thank you for your letter of 13 March on target animal safety testing (TAST) of veterinary vaccines.

It is not possible to confirm that all vaccines on sale in the UK for which target animal safety testing could be waived now have such waivers. This is because the waiver of TAST is a permissive provision which depends on data supplied by the marketing authorisation holder before it can be applied. VMD require data on 10 production batches to support a waiver application. The VMD actively encourages applications to waive the TAST and has published advice to manufacturers:

(http://www.vmd.gov.uk/Publications/MAVIS/Full/mavis69.pdf).

The current TAST waiver arrangements apply to the release of batches of authorised products where there is data to show that waiving TAST will not present an unacceptable risk to animals treated with that batch. Such data would not be available for new product applications and it is a legal requirement, from EC Directives 2001/82 and 2009/09, that pharmaceutical companies carry out a limited number of animal tests to demonstrate that the product is safe for the treated animal and efficacious in order to obtain a marketing authorisation. Therefore, we cannot adopt your proposed policy in relation to the animal data for a marketing authorisation application.

Work is already progressing on reducing the target animal safety testing required for non-EU markets. The Veterinary International Cooperation on Harmonisation (VICH) Steering Committee has clearly stated (VICH/07/038) its ambition to minimise animal testing and specifically expressed its support for the 3Rs principle (http://www.vichsec.org/pdf/07038-fin.doc). VICH strives to eliminate repetitious and unnecessary testing through harmonisation of regulatory requirements for the registration of veterinary products. The

UK's link to VICH is through advice provided by the European Medicines Agency and the VMD will continue to use this link to press for a reduction in target animal safety testing.

With regard to fees for variations to marketing authorisations to apply the TAST waiver the VMD will continue to waive these fees.

Yours sincerely

Jane Kennedy

RT HON JANE KENNEDY MP
