# Supplementary material for: Bridging the Gap Between Validation and Implementation of Non-Animal Veterinary Vaccine Potency Testing Methods
Source: Animals (Basel). 2011 Nov 29;1(4):414–32. doi: 10.3390/ani1040414 (PMC4513470; doi:10.3390/ani1040414)
Supplement: Supplementary File 1 [file animals-01-00414-s001.zip › supplementary materials/37 HO TABST.pdf]

From: Walsh Martin [Martin.Walsh@homeoffice.gsi.gov.uk]
Sent: 04 February 2010 16:22
To: Alistair Currie
Cc: Colston Angie
Subject: Vaccine testing

Dear Alistair

I hope you won't mind if I respond to your letter of 16 December 2009 by e-mail in the first instance. I will provide a letterheaded version in due course if you prefer. I have focused on the actions suggested in your letter.

A target date should be set for inspectors to assess their licensees and establish whether they are conducting TABST that could be waived

We have identified a number of project licences under which target animal quality control testing (which would include TABST) has recently been reported in statistical returns and, in discussion with the relevant HO inspectors, are now identifying other licences under which no testing has been carried out but which may contain authority for such testing. We expect to complete this exercise shortly.

> A deadline should then be given to licence holders to identify whether any TABST they conduct could be waived, and to report back to the HO

Discussions are also in hand with relevant project licence holders. All are aware of the waiver and have been asked to provide information on the status of the products tested relevant to TABST. Again we expect to complete this exercise shortly.

A realistic deadline, dependent on the individual circumstances, should then be set for the ending of any testing which could be waived

Where a waiver appears to be a possibility, we will ensure that the project licence holder takes prompt action.

Infringement proceedings should be undertaken where there is evidence that licence holders did not take the necessary steps to ensure that they met their obligations under standard condition six.

It is our general policy that appropriate action should be taken where there is evidence of non-compliance, either with the 1986 Act or licence conditions.

…that there are grounds for reviewing the HO's internal procedures, regarding, firstly, the identification of circumstances in which licensed procedures no longer meet the

requirements of condition 6 (particularly under generic licences) and, secondly, the action to be taken in those circumstances.

We do not accept that the current issue is indicative of a wider problem. However, we will take account of any lessons learned from it and include it as an agenda item for our next regular policy and management team meeting.

I will provide a further update in a month's time, or when our actions are complete, if that is earlier.

Regards

Martin

Martin Walsh

Animals Scientific Procedures Division
Home Office
4th Floor, South West
Seacole Building
2, Marsham Street
London
SW1P 4DF

Tel: +44 (0)20 7035 0746
Mob: +44 (0)7818 562246
E-mail: Martin.Walsh@homeoffice.gsi.gov.uk

Following publication of the Hannigan Report, all designated establishments have been advised of the risks of exchanging classes of non-encrypted information with the Home Office. Hannigan accepted that some stakeholders may nevertheless wish to continue to send/receive their own sensitive information that has not been encrypted. If in light of the risk you do not wish to [continue to] exchange non-encrypted communications with the Home Office, please let me know immediately and we will make alternative arrangements for securing future correspondence.

Individuals at designated establishments who do not already have access to our recommended encryption system are strongly advised to go to
http://scienceandresearch.homeoffice.gov.uk/animal-research/aboutus/EncryptionofDocuments/
to learn how to register with the CJSM System which has been selected by the Home Office to provide secure transmission of encrypted data. Registering with CJSM enables users to automatically encrypt Emails and attachments for sending to and from Home Office staff. Alternatively, it is also possible to manually encrypt sensitive information being sent to, or received from, the Home Office by Email or on removable electronic media (e.g. CDs) using the Home Office Encryption Bureau. For further information on how to use this service please telephone 020 8633 6047 or go to:
http://scienceandresearch.homeoffice.gov.uk/animal-research/aboutus/EncryptionofDocuments/

****
This email and any files transmitted with it are private and intended solely for the use of the individual or entity to whom they are addressed.
If you have received this email in error please return it to the address

it came from telling them it is not for you and then delete it from your system.

This email message has been swept for computer viruses.

****

The original of this email was scanned for viruses by the Government Secure Intranet virus scanning service supplied by Cable&Wireless in partnership with MessageLabs. (CCTM Certificate Number 2009/09/0052.) On leaving the GSi this email was certified virus free.
Communications via the GSi may be automatically logged, monitored and/or recorded for legal purposes.
