# Supplementary material for: Bridging the Gap Between Validation and Implementation of Non-Animal Veterinary Vaccine Potency Testing Methods
Source: Animals (Basel). 2011 Nov 29;1(4):414–32. doi: 10.3390/ani1040414 (PMC4513470; doi:10.3390/ani1040414)
Supplement: Supplementary File 1 [file animals-01-00414-s001.zip › supplementary materials/38 HO TABST.pdf]

From: Walsh Martin [mailto:Martin.Walsh@homeoffice.gsi.gov.uk]
Sent: 09 June 2010 14:35
To: Alistair Currie
Cc: Colston Angie
Subject: Target Animal Batch Safety Testing

Alistair

Here is the report on progress I promised. I am sorry it is a few days late.

We completed the review of project licences authorising TABS testing just before pre-election 'purdah' began. This established that a number of licences were used for this purpose in 2008, but that there is now only one licence under which the great majority of the testing is carried out. The place involved confirmed that it already has waivers for some of its vaccines and will apply for waivers for the rest.

The review also confirmed that relevant contract research organisations are aware of the waiver and do check whether their clients have applied for it.

One CRO which has not carried out any TABST in the last year wishes to continue to offer this test to clients as part of their vaccine development testing. Each request for the TABST will be reviewed by the project licence holder with respect to whether the waiver is applicable.

The general experience is that variations for removal of batch safety testing are approved without major objections within the EU, but that the timeframe for approval in some countries is long, delaying implementation in those cases.

Unfortunately, several non-EU countries do not accept waiving of batch safety testing (e.g. Korea, Taiwan, Thailand, Philippines and Brazil) and, so far, they have not been receptive to suggestions that they adopt the EU position. I think you may already be aware of this problem. Consequently, the batch safety test still has to be continuously performed for vaccines destined for these countries.

Separately, draft guidelines for Home Office inspectors have been prepared and will be finalised shortly. We are also preparing standard provisions on this issue for future inclusion in relevant project licences.

Regards

Martin Walsh

Animals Scientific Procedures Division
Home Office
4th Floor, South West
Seacole Building
2, Marsham Street
London
SW1P 4DF

Tel: +44 (0)20 7035 0746
Mob: +44 (0)7818 562246
E-mail: Martin.Walsh@homeoffice.gsi.gov.uk

Following publication of the Hannigan Report, all designated establishments have been advised of the risks of exchanging classes of non-encrypted information with the Home Office. Hannigan accepted that some stakeholders may nevertheless wish to continue to send/receive their own sensitive information that has not been encrypted. If in light of the risk you do not wish to [continue to] exchange non-encrypted communications with the Home Office, please let me know immediately and we will make alternative arrangements for securing future correspondence.

Individuals at designated establishments who do not already have access to our recommended encryption system are strongly advised to go to
http://scienceandresearch.homeoffice.gov.uk/animal-research/aboutus/EncryptionofDocuments/
to learn how to register with the CJSM System which has been selected by the Home Office to provide secure transmission of encrypted data. Registering with CJSM enables users to automatically encrypt Emails and attachments for sending to and from Home Office staff. Alternatively, it is also possible to manually encrypt sensitive information being sent to, or received from, the Home Office by Email or on removable electronic media (e.g. CDs) using the Home Office Encryption Bureau. For further information on how to use this service please telephone 020 8633 6047 or go to:
http://scienceandresearch.homeoffice.gov.uk/animal-research/aboutus/EncryptionofDocuments/

****

This email and any files transmitted with it are private and intended

solely for the use of the individual or entity to whom they are addressed.

If you have received this email in error please return it to the address

it came from telling them it is not for you and then delete it from your system.

This email message has been swept for computer viruses.

****

The original of this email was scanned for viruses by the Government Secure Intranet virus scanning service supplied by Cable&Wireless Worldwide in partnership with MessageLabs. (CCTM Certificate Number 2009/09/0052.) On leaving the GSi this email was certified virus free.
Communications via the GSi may be automatically logged, monitored and/or recorded for legal purposes.
