# Supplementary material for: Bridging the Gap Between Validation and Implementation of Non-Animal Veterinary Vaccine Potency Testing Methods
Source: Animals (Basel). 2011 Nov 29;1(4):414–32. doi: 10.3390/ani1040414 (PMC4513470; doi:10.3390/ani1040414)
Supplement: Supplementary File 1 [file animals-01-00414-s001.zip › supplementary materials/39 USDA LPA erysipelas.pdf]

USDA

United States Department of Agriculture

Animal and Plant Health Inspection Service

Legislative and Public Affairs

Freedom of Information

4700 River Road
Unit 50
Riverdale, MD
20737-1232

August 15, 2011

Jeffery Brown
People for the Ethical Treatment of Animals
2892 Rowena Avenue, Suite 102
Los Angeles, California 90039

Dear Mr. Brown:

This is in response to your July 15, 2010, Freedom of Information Act (FOIA) request for all information relating to Fort Dodge Laboratories, Inc., Lot Release Protocol for the testing of USDA Code # 1541.10, Erysipelothrix Rhusiopath July 16, 2010, and assigned case number FOIA 10-557. We apologize for the delay of this response.

Agency employees conducted a thorough search of their files and located seven pages of records responsive to your request. The information you have requested is being withheld pursuant to FOIA Exemption 4, 5 U.S.C. § 552(b)(4). This exemption protects confidential business information from disclosure when release would cause substantial harm to the competitive position of an individual, a partnership or a corporation from whom the information was obtained. Specifically,
the outline of production has been withheld in accordance with the guidelines of this exemption.

You may appeal our denial of this information. If you choose to appeal, your appeal must be in writing and must be received within 45 days of the date of this letter to:

Administrator
Animal and Plant Health Inspection Service
Ag Box 3401
Washington, D.C. 20250-3401

Please refer to FOIA 10-557 in your appeal letter and add the words "FOIA Appeal" to the front of the envelope. To assist the Administrator in reviewing your appeal, provide specific reasons why you believe modification of the determination is warranted.

Due to our delay processing your request, the fee has been waived. If you have any questions, please contact Ms. Deborah L. Leilich of my staff at (301) 734-3623.

Sincerely,

Tonya G. Woods
Director
Freedom of Information & Privacy Act
Legislative and Public Affairs

Enclosure
