# Supplementary material for: Bridging the Gap Between Validation and Implementation of Non-Animal Veterinary Vaccine Potency Testing Methods
Source: Animals (Basel). 2011 Nov 29;1(4):414–32. doi: 10.3390/ani1040414 (PMC4513470; doi:10.3390/ani1040414)
Supplement: Supplementary File 1 [file 23 PETA erysipelas.pdf › supplementary materials/23 PETA erysipelas.pdf]

PeTA
PEOPLE FOR THE ETHICAL TREATMENT OF ANIMALS

HEADQUARTERS
501 FRONT STREET
NORFOLK, VA 23510
TEL 757-622-PETA
FAX 757-622-0457

August 19, 2009

Richard E. Hill, Jr., Director
Center for Veterinary Biologics
Animal and Plant Health Inspection Service
1920 Dayton Ave.
Ames, IA 50010

Via email (rick.e.hill@usda.gov) and fax (515-232-7120)

Dear Dr. Hill,

On behalf of PETA's more than two million members and supporters who are concerned about the suffering of animals in laboratory experiments, particularly in applications for which there are accepted replacements for animal methods, we would like to know if USDA currently accepts or plans to accept results from an ECVAM-validated *in vitro* assay in place of the painful *in vivo* vaccine challenge procedure for determining the batch potency of an inactivated erysipelas vaccine as described in Supplemental Assay Method (SAM) 606.

SAM 606 has been unchanged since 1982 and recommends administering virulent *Erysipelothrix rhusiopathiae* to vaccinated and unvaccinated pigs in order to determine the immunogenicity of a given vaccine batch. This challenge procedure, by definition, causes severe suffering in test animals. In accordance with 9 CFR 113.67, evidence of a "satisfactory challenge" in unvaccinated animals includes "acute illness with hyperemia of the abdomen and ears, possibly terminating in sudden death; moribundity, with or without metastatic skin lesions; depression with anorexia, stiffness and/or joint involvement; or any combination of these symptoms and lesions."

Fortunately, a replacement assay that does not require challenge has been developed. At its 2002 meeting, the European Centre for the Validation of Alternative Methods (ECVAM) and the ECVAM Scientific Advisory Committee (ESAC) unanimously endorsed the use of the ELISA method as a validated procedure for measuring the potency of inactivated swine erysipelas vaccines. This procedure has already been integrated into the current European Pharmacopoeia (Ph. Eur.) 6.0, and addresses the mechanism of protection by taking an immunochemical approach. It quantifies anti-erysipelas antibodies in pooled sera from vaccinated mice, thereby avoiding the pain and distress caused by the challenge procedure. Recognizing the superior accuracy, reproducibility and repeatability of ELISA, Ph. Eur. 6.0 now directly states that an immune challenge test for each batch of inactivated erysipelas vaccine is not necessary.

Considering the scientific and regulatory acceptance this assay has found in other settings, we would appreciate your providing us with the USDA's position on the use of results from an ELISA-based test of inactivated swine erysipelas batch potency. I have attached the international validation study for your review. Please do not hesitate to contact me directly at (323) 644-7382 extension 32 or via email at JeffreyB@peta.org regarding this important matter.

Sincerely,

Jeffrey Brown
Research Associate
Regulatory Testing Division
